# Appraising the causal role of smoking in multiple diseases: A systematic review and meta-analysis of Mendelian randomization studies

**DOI:** 10.1016/j.ebiom.2022.104154

**Published:** 2022-07-08

**Authors:** Susanna C. Larsson, Stephen Burgess

**Affiliations:** a Unit of Medical Epidemiology, Department of Surgical Sciences, Uppsala University, Uppsala, Sweden; b Unit of Cardiovascular and Nutritional Epidemiology, Institute of Environmental Medicine, Karolinska Institutet, Stockholm, Sweden; c Department of Public Health and Primary Care, University of Cambridge, Cambridge, UK; d MRC Biostatistics Unit, University of Cambridge, Cambridge, UK

**Keywords:** Cancer, Cardiovascular disease, Chronic diseases, Lifestyle factors, Mendelian randomization, Smoking

## Abstract

**Background:**

The causal association between cigarette smoking and several diseases remains equivocal. The purpose of this study was to appraise the causal role of smoking in a wide range of diseases by summarizing the evidence from Mendelian randomization (MR) studies.

**Methods:**

MR studies on genetic liability to smoking initiation or lifetime smoking (composite of smoking initiation, heaviness, duration, and cessation) in relation to circulatory system, digestive system, nervous system, musculoskeletal system, endocrine, metabolic, and eye diseases, and neoplasms published until February 15, 2022, were identified in PubMed. De novo MR analyses were performed using summary statistics data from genome-wide association studies. Meta-analysis was applied to combine study-specific estimates.

**Findings:**

Meta-analyses of findings of 29 published MR studies and 123 de novo MR analyses of 57 distinct primary outcomes showed that genetic liability to smoking (smoking initiation or lifetime smoking) was associated with increased risk of 13 circulatory system diseases, several digestive system diseases (including diverticular, gallstone, gastroesophageal reflux, and Crohn's disease, acute pancreatitis, and periodontitis), epilepsy, certain musculoskeletal system diseases (including fracture, osteoarthritis, and rheumatoid arthritis), endocrine (polycystic ovary syndrome), metabolic (type 2 diabetes) and eye diseases (including age-related macular degeneration and senile cataract) as well as cancers of the lung, head and neck, esophagus, pancreas, bladder, kidney, cervix, and ovaries, and myeloid leukemia. Smoking liability was associated with decreased risk of Parkinson's disease and prostate cancer.

**Interpretation:**

This study found robust evidence that cigarette smoking causes a wide range of diseases.

**Funding:**

This work was supported by research grants from the Swedish Cancer Society (Cancerfonden), the Swedish Heart Lung Foundation (Hjärt-Lungfonden, 20210351), the Swedish Research Council for Health, Working Life and Welfare (Forte, 2018-00123), and the Swedish Research Council (Vetenskapsrådet, 2019-00977). Stephen Burgess is supported by Sir Henry Dale Fellowship jointly funded by the Wellcome Trust and the Royal Society (204623/Z/16/Z) and the National Institute for Health Research Cambridge Biomedical Research Centre (BRC-1215-20014). The views expressed are those of the authors and not necessarily those of the National Institute for Health Research or the Department of Health and Social Care.

**Research in context**

**Evidence before this study**

It is well known that smoking causes lung diseases and certain circulatory system diseases, particularly coronary heart disease and stroke. Nevertheless, the causality of the association of smoking with other circulatory system diseases as well as digestive system, nervous system, musculoskeletal system, endocrine, metabolic and eye diseases and certain neoplasms is unestablished.

**Added value of this study**

In this meta-analysis of results of published Mendelian randomization studies and de novo Mendelian randomization analyses, genetic liability to smoking was associated with increased risk of all but one of the 14 studied circulatory system diseases, several digestive system diseases, epilepsy, certain musculoskeletal system diseases, endocrine, metabolic, and eye diseases as well as nine site-specific cancers.

**Implications of all the available evidence**

These findings support a causal effect of cigarette smoking in wide range of diseases.

Alt-text: Unlabelled box

## Introduction

Tobacco smoke comprises thousands of compounds that may have harmful effects on most organs of the body.^1^^,^^2^ It is well known that smoking causes lung cancer,^2^ chronic obstructive pulmonary disease, and some circulatory system diseases, particularly coronary heart disease and stroke.^1^^,^^3^ However, the causality of the association of smoking with other circulatory system diseases as well as digestive system, nervous system, musculoskeletal system, endocrine, metabolic and eye diseases and certain neoplasms remains unestablished. During the last few years, the potential causal association between smoking and risk of different diseases has been investigated using the Mendelian randomization (MR) approach.^4^, ^5^, ^6^, ^7^, ^8^, ^9^, ^10^, ^11^, ^12^, ^13^, ^14^, ^15^, ^16^, ^17^, ^18^, ^19^, ^20^, ^21^, ^22^, ^23^, ^24^, ^25^, ^26^, ^27^, ^28^, ^29^, ^30^, ^31^, ^32^ MR is a method that utilizes genetic variants associated with a difference in the exposure (e.g., smoking liability) as instrumental variable for the exposure to determine the causal role of the exposure in the development of disease.^33^ The MR design mitigates confounding because genetic alleles are randomly allocated when passed from parents to offspring and therefore usually not related to other risk factors. Furthermore, MR studies are protected against bias from reverse causation as genetic variants are not modified by the progression of disease.

To comprehensively appraise and summarize the evidence on the causal role of smoking in a wide range of diseases, we carried out a systematic review of published MR studies. Additionally, we conducted de novo MR analyses utilizing genome-wide association study (GWAS) summary statistics data from the FinnGen study and other publicly available GWAS. Finally, we performed meta-analyses to summarize the results of published and de novo MR analyses.

## Methods

### Literature search and inclusion criteria

PubMed was searched for pertinent MR studies published until February 15, 2022, using the search term “Mendelian randomization” combined with “smoking”. Original full-text articles that presented results for the associations of genetic liability to smoking initiation or lifetime smoking with risk of circulatory, digestive, nervous, and musculoskeletal system diseases, endocrine, metabolic, and eye diseases, and neoplasms. When more than one publication based on the same GWAS for the outcome (same participants), only the publication with the largest sample size for the outcome or the earliest published study (if the sample size was the same) was included. No restriction based on sample size was imposed. Studies using only a single or few (<10) genetic instruments for nicotine dependence or smoking behavior or quantity were excluded as those studies were few and estimates from those studies could not be meta-analyzed with other eligible studies due to different unit of the exposure. The review was not registered, and no protocol is available. The study was performed according to PRISMA and STROBE-MR guidelines.

### Data extraction

Data extracted from every study included family name of the first author and publication year; consortium or study from which the genetic variants for the smoking exposure was retrieved; consortium or study from which genetic association estimates for the disease were retrieved; sample size (i.e., number of cases and non-cases); and the relative risk estimate (odds ratio [OR]) with corresponding 95% confidence interval (CI) for the smoking-disease association from the main analysis based on the inverse-variance weighted method as well as from sensitivity analyses based on the weighted median and MR-Egger methods and multivariable MR analysis with adjustment for genetically predicted alcohol consumption. One investigator (SCL) extracted the data, which were checked by another investigator (SB).

### De novo MR analyses

De novo MR analyses using summary statistics genetic data from the FinnGen study^34^ were conducted wherever MR results from this study were unavailable. We used data from the R6 data release, which includes 260,405 Finnish individuals after omitting those with ambiguous sex, non-Finnish origin, genotype missingness over 5%, or excess heterozygosity (±4 standard deviations).^34^ The GWAS analyses were adjusted for age, sex and the first ten genetic principal components.^34^ Diseases were defined by the International Classification of Diseases codes (8th, 9th and 10th revisions) with information obtained from nationwide registries. Details about genotype data, methods, and quality control have been described elsewhere.^34^

De novo MR analyses were also conducted for lifetime smoking in relation site-specific cancer risk using the same outcome data as in a previous study on smoking initiation and cancer.^30^ Additionally, de novo MR analyses were performed for osteoarthritis, gout, and primary open-angle glaucoma using publicly available summary statistics data from GWAS meta-analyses of these outcomes.^35^, ^36^, ^37^ For all de novo MR analyses, independent genetic variants (low linkage disequilibrium, defined as R^2^<0.01) associated with smoking initiation or lifetime smoking at the genome-wide significance level (*P*<5 × 10^−8^) in previous smoking GWAS analyses^38^^,^^39^ were applied as instrumental variables.

The MR analyses based on FinnGen data included 297 and 124 SNPs as instrumental variables for smoking initiation and lifetime smoking, respectively (Table S1). The analyses were performed with the mrrobust command in Stata (StataCorp, College Station, Texas, USA) for analyses of FinnGen and the MR-Base platform^40^ for analyses of osteoarthritis, gout, and primary open-angle glaucoma. The inverse-variance weighted (multiplicative random-effects model) method was used as main analysis, whereas the weighted median and MR-Egger methods were used as sensitivity analyses.^41^ As smoking initiation has a modest genetic correlation with alcohol consumption (*r*_g_=0.34),^38^ we performed multivariable MR analysis with adjustment for genetically predicted alcohol consumption. Summary statistics data for genetically predicted alcohol consumption (drinks per week) were obtained from the same GWAS from which the smoking initiation instruments were obtained from.^38^ The GWAS analysis of alcohol consumption included 941,280 European descent individuals.^38^ The FinnGen biobanks and cohorts were not included in the smoking and alcohol GWAS analyses.

### Meta-analysis

When two or more MR estimates were available for the same outcome based on non-overlapping samples, a combined estimate was obtained via meta-analysis using the metan command in Stata. Figures were created in RStudio. All reported estimates were expressed per standard deviation increase in genetic liability to smoking initiation (prevalence of ever smoked regularly) and lifetime smoking index. Sensitivity analyses were conducted using estimates derived from the weighted median and MR-Egger methods and multivariable MR analysis adjusted for genetically predicted alcohol consumption. In addition, consortia that included individuals of non-European ancestries were removed in sensitivity analyses.

### Role of the funding source

The funders had no role in study design; in the collection, analysis, and interpretation of data; in the writing of the report; and in the decision to submit the paper for publication.

### Ethics

The MR analyses of FinnGen data were approved by the Swedish Ethical Review Board (no. 2019-02793). All published studies included in the meta-analyses were approved by a relevant ethical review board, and participants had provided informed consent.

### Findings

#### Literature search, study selection, and de novo MR analyses

The literature search resulted in 385 hits of which 54 original articles presented findings for genetic liability to smoking initiation or lifetime smoking in relation to one or more of the contemplated outcomes. After exclusion of studies using overlapping or same outcome data, 29 articles based on non-overlapping populations were eligible for inclusion in one or more meta-analyses. The number of studies included in each outcome category was seven for circulatory system diseases,^4^, ^5^, ^6^, ^7^, ^8^, ^9^, ^10^ six for digestive system diseases,^11^, ^12^, ^13^, ^14^, ^15^, ^16^ six for nervous system diseases,^17^, ^18^, ^19^, ^20^, ^21^ three for musculoskeletal system outcomes,^22^, ^23^, ^24^ two for endocrine and metabolic diseases,^25^^,^^26^ two for eye diseases,^27^^,^^28^ and four for neoplasms.^29^, ^30^, ^31^, ^32^ In addition to the included studies, 123 de novo MR analyses (*n*=51 for smoking initiation, *n* = 72 for lifetime smoking) were conducted. A total of 57 distinct outcomes were analyzed. Figure S1 summarizes the study selection and study design.

#### Study description

All but one study of smoking initiation used genetic variants from the GWAS and Sequencing Consortium of Alcohol and Nicotine consortium, which encompassed 1 232 091 individuals of European ancestry in the analysis of smoking initiation, defined as ever smoked regularly.^38^ One study^17^ used genetic variants for smoking initiation obtained from a GWAS of 518 633 individuals in the Tobacco and Genetics Consortium and UK Biobank.^42^ That GWAS identified 172 genome-wide-significant conditional associations.^42^ The number of genetic variants used as instrumental variables for smoking initiation ranged from 74 to 378 and explained up to 2.4% of the phenotypic variance. The genetic variants for lifetime smoking were in all studies obtained from a GWAS of 462 690 individuals in the UK Biobank where lifetime smoking was defined as a composite of smoking initiation, heaviness, duration, and cessation.^39^ The 126 genetic variants for lifetime smoking explained around 0.3% of the phenotypic variance.^39^ All MR studies used outcome data from a large GWAS meta-analysis (consortium), UK Biobank, FinnGen study, Million Veteran Program, or two or more of these data sources. All studies provided results for smoking initiation based on the main inverse-variance weighted method, and most studies presented results based on the weighted median and MR-Egger methods. Estimates for smoking initiation adjusted for alcohol consumption were reported in some studies.^4^, ^5^, ^6^^,^^8^^,^^23^^,^^27^^,^^29^^,^^30^ Studies included in the main MR analyses and corresponding results are shown in Table S3 for smoking initiation and Table S4 for lifetime smoking.

#### Circulatory system disease

**Figure 1:**
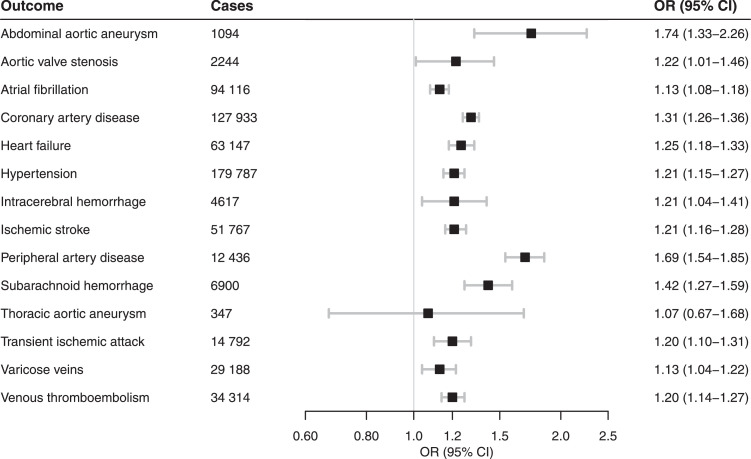
Meta-analysis results of the association between genetic liability to smoking initiation and risk of circulatory system diseases. Estimates are odds ratios (OR) with 95% confidence intervals (CI) for a one standard deviation increase in genetic liability to smoking initiation.

Genetic liability to smoking initiation was associated with elevated risk of all but one (thoracic aortic aneurysm) of the 14 distinct circulatory system diseases (Figure 1; Table S2). Among subtypes of venous thromboembolism, the association was similar for deep vein thrombosis and pulmonary embolism (Table S2). Genetic liability to lifetime smoking was associated with increased risk of the 13 circulatory system disease (Table S3).

#### Digestive system diseases

**Figure 2:**
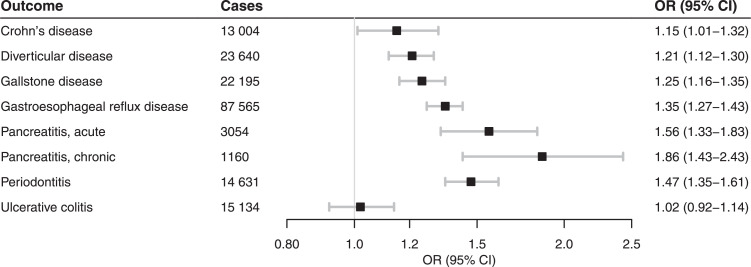
Meta-analysis results of the association between genetic liability to smoking initiation and risk of digestive system diseases. Estimates are odds ratios (OR) with 95% confidence intervals (CI) for a one standard deviation increase in genetic liability to smoking initiation.

Genetic liability to smoking initiation was associated with increased risk of diverticular, gallstone, gastroesophageal reflux, and Crohn's disease as well as pancreatitis (both acute and chronic) and periodontitis, but not with ulcerative colitis (Figure 2; Table S2). Results were consistent for lifetime smoking (Table S3).

#### Nervous system diseases

**Figure 3:**
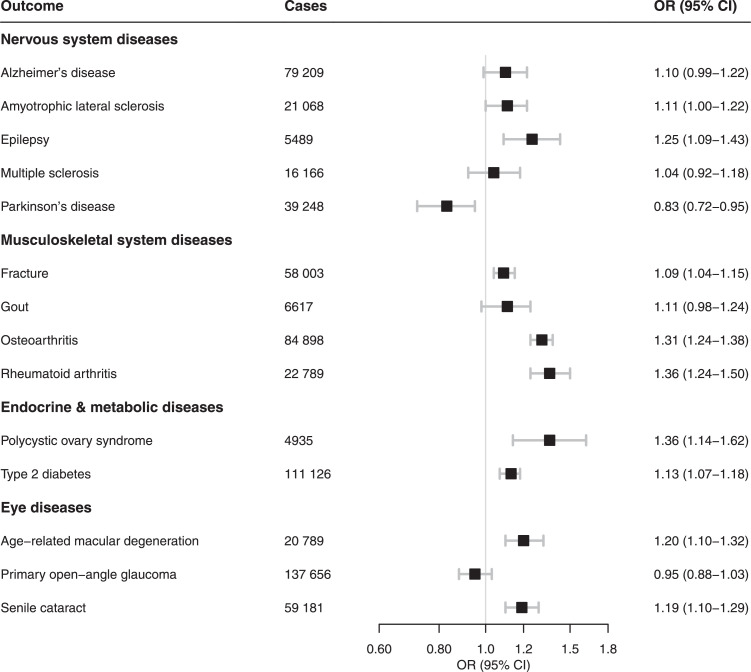
Meta-analysis results of the association between genetic liability to smoking initiation and risk of nervous system, musculoskeletal system, endocrine and metabolic, and eye diseases. Estimates are odds ratios (OR) with 95% confidence intervals (CI) for a one standard deviation increase in genetic liability to smoking initiation.

Among the nervous system diseases, a consistent association with genetic liability to smoking was observed for epilepsy (increased risk) and Parkinson's disease (decreased risk) (Figure 3, Table S2). No strong and coherent evidence of association was observed for smoking and risk of amyotrophic lateral sclerosis, Alzheimer's disease, or multiple sclerosis (Figure 3, Table S2, Table S3).

#### Musculoskeletal system diseases

Genetic liability to smoking initiation was associated with higher risk of fracture, osteoarthritis and rheumatoid arthritis, but not gout (Figure 3; Table S2). Data on the two main types of rheumatoid arthritis were available in the FinnGen study. De novo MR analysis based on data from this study showed that genetic liability to smoking initiation had a similar association with seropositive (OR 1.31, 95% CI 1.14-1.52, *n =*5867 cases) and seronegative (OR 1.27, 95% CI 1.03-1.56, *n =*2484 cases) rheumatoid arthritis. Results for lifetime smoking were consistent with those for smoking initiation (Table S3).

#### Endocrine and metabolic diseases

Genetic liability to smoking initiation was strongly associated with a higher risk of polycystic ovary syndrome and weakly associated with a higher risk of type 2 diabetes (Figure 3; Table S2). Lifetime smoking was weakly associated with polycystic ovary syndrome and type 2 diabetes (Table S3).

#### Eye diseases

Genetic liability to smoking initiation was related to increased risk of age-related macular degeneration and senile cataract but not primary open-angle glaucoma (Figure 3; Table S2). The lifetime smoking instrument was also associated with age-related macular degeneration but had only a weak and non-significant positive association with senile cataract (Table S3).

#### Neoplasms

**Figure 4:**
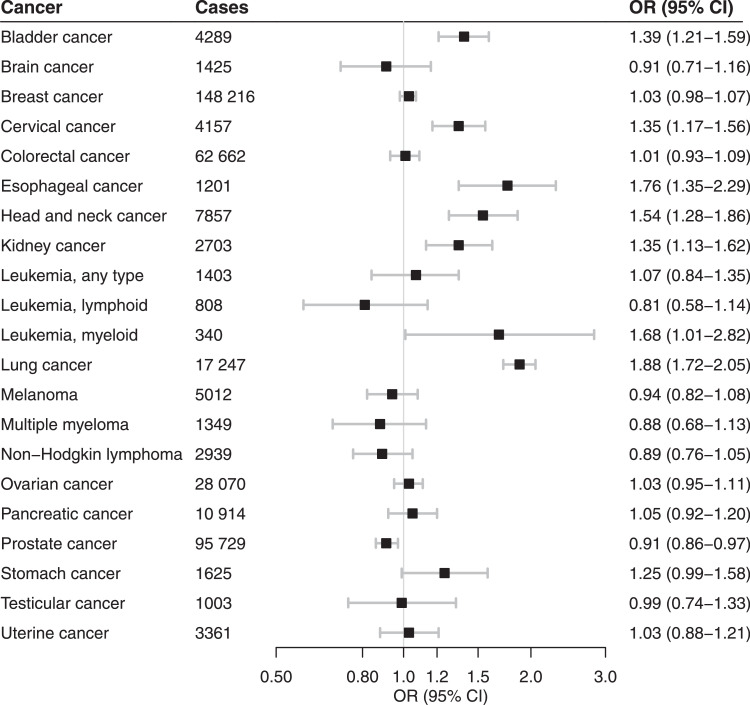
Meta-analysis results of the association between genetic liability to smoking initiation and risk of neoplasms. Estimates are odds ratios (OR) with 95% confidence intervals (CI) for a one standard deviation increase in genetic liability to smoking initiation.

Genetic liability to smoking initiation was associated with increased risk of cancers of the lung, head and neck, esophagus, bladder, kidney, and cervix as well as myeloid leukemia, but with a decreased risk of prostate cancer (Figure 4; Table S2). The genetic instrument for lifetime smoking was also associated with increased risk of cancers of the lung, head and neck, esophagus, bladder, kidney, and cervix and non-significantly associated with a reduced risk of prostate cancer (Table S3). This instrument was additionally associated with increased risk of pancreatic and ovarian cancers (Table S3).

#### Sensitivity analyses

Results based on the weighted median and MR-Egger methods were broadly consistent with the primary findings, albeit with low to very low (in some MR analyses) precision, and there was limited evidence from the MR-Egger test that he observed associations may be biased by directional pleiotropy (Table S4 and Table S5). An exception was for the association between genetic liability to lifetime smoking and gallstone disease, which was inverse in the MR-Egger analysis (Table S5). Removing two studies that included non-European ancestry individuals did not change the results materially; the OR for the association between smoking initiation and atrial fibrillation and coronary artery disease was 1.23 (95% CI 1.10-1.36) and 1.33 (95% CI 1.27-1.40), respectively. Finally, most of the observed associations between genetic liability to smoking initiation and disease risk persisted after adjustment for genetically predicted alcohol consumption; the exception was the association with chronic pancreatitis, which was attenuated close to the null (Table S6).

## Discussion

This systematic review with meta-analyses of published and de novo MR analysis results found genetic evidence that smoking liability is related to increased risk of most circulatory system diseases, several diseases of the digestive system (including diverticular, gallstone, gastroesophageal reflux, and Crohn's disease, acute pancreatitis, and periodontitis), epilepsy, certain musculoskeletal system diseases (including fracture, osteoarthritis, and rheumatoid arthritis), and some endocrine (polycystic ovary syndrome), metabolic (type 2 diabetes) and eye diseases (including age-related macular degeneration and senile cataract). Among cancers, genetic liability to smoking initiation or lifetime smoking was associated with an increased risk of cancers of the lung, head and neck, esophagus, pancreas, bladder, kidney, cervix, and ovaries as well as myeloid leukemia. In contrast, there was evidence of inverse associations between smoking liability and risk of Parkinson's disease and prostate cancer.

Our findings from meta-analyses of MR studies are in line with observational studies, which have found that smoking is associated with increased risk of several circulatory^1^^,^^3^^,^^43^ and digestive system diseases^44^, ^45^, ^46^, ^47^, ^48^ as well as some (e.g., fracture^49^ and rheumatoid arthritis^50^) but not all (e.g., osteoarthritis)^51^ musculoskeletal system diseases. Smoking could increase the risk of these diseases through several biological mechanisms of which a central plausible mechanism is via systemic inflammation. The burning of cigarettes generates reactive oxygen species that can activate epithelial cell intracellular signaling cascades that lead to inflammatory gene activation, such as tumor necrosis factor.^52^ Systemic inflammation and related inflammatory markers (e.g., tumor necrosis factor) are associated with increased risk of several circulatory system diseases, inflammatory bowel disease, and rheumatoid arthritis.^53^ Smoking may also increase the risk of these diseases by promoting endothelial dysfunction and thrombogenesis and via effects on immune responses and microbial composition.^54^^,^^55^ Furthermore, nicotine exposure may cause acute cardiovascular events through increased myocardial contractility and vasoconstriction, with resulting increased myocardial work and oxygen requirement and reduced coronary and cerebral blood flow.^55^

Among the inflammatory bowel diseases, genetic liability to smoking was associated with an increased risk of Crohn's disease but not ulcerative colitis; the former disease can occur wherever in the digestive tract, whereas the latter solely affects the colon. This differential effect of smoking on two inflammatory bowel diseases has been found also in observational studies^45^ and might be related to that the hazardous compounds in smoke more readily reach the upper digestive tract and there has a local detrimental effect. Nevertheless, the CIs largely overlapped for the associations of smoking with these two diseases. Thus, low power could possibly explain the disparate findings.

No coherent association between smoking and risk of different nervous system diseases has been reported by observational studies.^56^, ^57^, ^58^, ^59^, ^60^ The present meta-analysis of MR studies found evidence in support of a possible causal relationship between smoking and reduced risk of Parkinson's disease and increased epilepsy risk, but no clear and consistent evidence of a role of smoking in Alzheimer's disease and multiple sclerosis. Smoking has been consistently associated with a decreased risk of Parkinson's disease in observational studies, but it was unclear whether this association is causal.^56^ A potential mechanism behind the protective association is the stimulating effects of cigarette smoke on dopamine release.^56^ However, it cannot be ruled out that any bias explains the observed association. In contrast to Parkinson's disease, no coherent and strong association has been observed for smoking and risk of Alzheimer's disease^57^ and multiple sclerosis^58^ in observational studies, which is in line with our MR findings. If anything, previous studies have suggested a positive association between smoking and risk of Alzheimer's disease^57^ and multiple sclerosis.^58^ Observational data on smoking and epilepsy risk are scarce. A statistically significant association between smoking and incidence of late-onset epilepsy was observed in one cohort study.^59^ Another cohort study with only 55 incident late-onset cases of epilepsy showed a nearly 2-fold non-significant increased risk of epilepsy in current smokers compared with never smokers.^60^

There was a paucity of observational studies on smoking in relation to polycystic ovary syndrome. In this study, the association between genetic liability to smoking initiation and increased risk of polycystic ovary syndrome was consistent across the two MR studies and in sensitivity analyses. No association was found for lifetime smoking, but this analysis was only based on FinnGen, which included few cases, and the precision of the result was low and should be interpreted with caution. A detrimental effect of smoking on polycystic ovary syndrome may be related to effects on pituitary, thyroid, adrenal, testicular, and ovarian function as well as changes in the levels of androgens and other hormones.^61^^,^^62^

A meta-analysis of observational prospective studies found a 37% and 14% higher risk of type 2 diabetes in current and former smokers, respectively, compared with never smokers.^63^ Our findings based on MR studies support a weak association between smoking and type 2 diabetes, an association that might be related to reduced insulin sensitivity among smokers.^61^

Smoking has been associated with an increased risk of any form of age-related macular degeneration^64^^,^^65^ and age-related (senile) cataract^66^ in observational studies, associations that were corroborated in the present meta-analysis of MR studies. The mechanism behind the detrimental effect of smoking on these eye diseases is not fully understood but may be explained by oxidative stress and the generation of reactive oxygen species that can damage the eye.

Tobacco smoke contains many compounds with established carcinogenic potency and is therefore expected to cause cancer in humans.^2^ The present study extends the evidence from observational studies (case-control and cohort studies) which have found that smoking is associated with an increased risk of a plurality of cancers, including cancers of the lung, head and neck, esophagus, pancreas, bladder, kidney, cervix, and ovaries as well as myeloid leukemia. Observational data have additionally indicated that heavy smoking may increase the risk of colorectal cancer (both colon and rectal cancers).^2^^,^^67^ A non-significant positive association was observed between the genetic instrument for lifetime smoking and colorectal cancer risk in three individual MR studies and in the present meta-analysis of those studies. It is possible that the MR studies could not capture heavy smoking and therefore was unable to confirm the observational association between smoking and colorectal cancer. Whether the observed inverse association between smoking liability and prostate cancer risk is causal or due to bias is unclear. The association may be a result of detection bias as prostate cancer is often undiagnosed until an individual has another disease.

An important strength of MR studies is that common biases inherent in observational studies, including confounding and reverse causation, are diminished because the exposure (e.g., smoking) of interest is proxied by genetic variants that are usually unrelated to other risk factors and are not changed by the development of disease. Nevertheless, the validity of MR results depends on three principal assumptions. First, the genetic instruments should be strongly associated with the exposure of interest (assumption 1, relevance). Both in our de novo MR analyses and the studies included in the meta-analyses, the selected SNPs were associated with genetic liability to smoking at the genome-wide significance threshold and the instrument strength was good as indicated by F-statistic above 10 for the used SNPs (Table S1). Second, the instrumental variables should not be associated with potential confounding factors of the exposure-outcome relationship (assumption 2, independence). Genetic liability to smoking initiation is modestly associated with genetically predicted alcohol consumption. However, the observed associations of genetic liability to smoking initiation with disease risk remained, except for chronic pancreatitis, after adjustment for genetically predicted alcohol consumption. In addition, findings were broadly consistent for smoking initiation and lifetime smoking which were based on different genetic instruments. The only exception was for polycystic ovary syndrome as well as pancreatic and ovarian cancers for which the two genetic instruments generated discordant results. Third, the genetic instruments should influence the outcome only via the exposure, not via other alternative pathways (assumption 3, exclusion restriction). We could not completely exclude the possibility that the findings might be biased by pleiotropy. Nonetheless, most studies presented results based on the weighted median and MR-Egger methods, which are more robust to pleiotropy, and found consistent results in these sensitivity analyses. Several estimates from the MR-Egger analyses had low to very low precision (as indicated by broad 95% CIs) and should be interpreted cautiously.

As in any MR study of a harmful exposure and a late-onset disease, competing risk bias (a form of survival bias) might have influenced some of the findings. This bias generally increases with higher age at study inclusion.^68^ Selection bias could also affect MR results but this bias is likely to have a less impact compared with other biases.^69^ Selection bias can influence MR findings when selection into the study depends on a collider between the genetic variant and confounders of the exposure–outcome association.^69^ Another potential bias in MR investigations is population stratification bias. In this meta-analysis, nearly all included studies comprised individuals of European-descent only and residual stratification bias was adjusted for by genetic principal components. Moreover, the observed associations remained when multi-ethnic GWAS were excluded.

A limitation of our study is that the magnitude of the OR estimates cannot be compared with the relative risk estimates from observational studies. In the MR analyses, the ORs are expressed per standard deviation increase in genetic liability to smoking initiation (prevalence of ever smoked regularly) and for lifetime smoking, which is a composite index that captures smoking duration, heaviness, and cessation.^70^ In observational studies, relative risk estimates were generally reported for current or ever smokers versus never smokers or for the number of cigarettes smoked during a certain period in life or the number of pack-years of smoking. Nevertheless, the MR findings for each genetic instrument for smoking can be compared across diseases. Genetic liability to smoking initiation had a particularly strong and reliable association with certain circulatory system diseases (especially abdominal aortic aneurysm, peripheral artery disease, and subarachnoid hemorrhage), acute pancreatitis, periodontitis, rheumatoid arthritis, polycystic ovary syndrome, and certain cancers (especially cancers of the lung, esophagus, head and neck, and bladder), with OR estimates above 1.35.

Besides the diseases studied in the present study, previous MR studies have shown that smoking liability is associated with increased risk of COVID-19 and other respiratory infections (e.g., pneumonia and lower and upper respiratory infections)^71^ and pregnancy loss^72^ as well as decreased longevity.^73^ In addition, some MR studies have found bi-directional associations between smoking liability and mental disorders, such as anxiety,^74^ depression,^70^^,^^74^ bipolar disorders,^74^^,^^75^ and schizophrenia.^70^^,^^74^

In conclusion, this study provided genetic evidence to support that smoking is a causal risk factor for a wide range of diseases. Thus, reducing smoking initiation is a key public health priority.

## Contributors

S.C.L. conducted the literature search, data extraction, and statistical analyses, and drafted the manuscript. S.B. reviewed the data extraction and revised the manuscript for intellectual content. Both authors contributed to data interpretation and approved the final version of the manuscript. S.C.L. had full access to all the data in the study and takes responsibility for the integrity of the study. All authors read and approved the manuscript.

## Data sharing statement

All the data supporting the conclusions of this article are included within the article and its supplementary files.

## Declaration of interests

The authors declare no competing interests.

## Appendix Supplementary materials

Image, application 1
