# Supplementary material for: Appraising the causal role of smoking in multiple diseases: A systematic review and meta-analysis of Mendelian randomization studies
Source: eBioMedicine. 2022 Jul 8;82:104154. doi: 10.1016/j.ebiom.2022.104154 (PMC9278068; doi:10.1016/j.ebiom.2022.104154)
Supplement: Supplementary file 1 [file mmc1.docx]

**Supplementary material to Appraising the causal role of smoking in multiple diseases: A systematic review and meta-analysis**

**of Mendelian randomization studies**

| **Supplemental figure/table** | **Page** |
| --- | --- |
| **Figure S1.** Literature review and study design | 2 |
| **Table S1.** Single-nucleotide polymorphisms used as instrumental variables in the de novo Mendelian randomization analyses of FinnGen data | 3 |
| **Table S2.** Mendelian randomization studies included in the meta-analyses of genetic liability to smoking initiation in relation to circulatory system, digestive system, nervous system, musculoskeletal system, endocrine, metabolic and eye diseases, and neoplasms | 18 |
| **Table S3.** Mendelian randomization studies included in the meta-analyses of genetic liability to lifetime smoking in relation to circulatory system, digestive system, nervous system, musculoskeletal system, endocrine, metabolic and eye diseases, and neoplasms | 25 |
| **Table S4.** Sensitivity analysis results for genetic liability to smoking initiation and studied outcomes based on the weighted median and MR-Egger methods | 31 |
| **Table S5.** Sensitivity analysis results for genetic liability to lifetime smoking and the studied outcomes based on the weighted median and MR-Egger methods | 38 |
| **Table S6.** Sensitivity analysis results for genetic liability to smoking initiation and disease risk adjusted for genetically predicted  alcohol consumption | 44 |

**Figure S1.** Literature review and study design

*Inclusion criteria: Original full-text article that presents results for the associations of genetic liability to smoking initiation or lifetime smoking with risk of one or more circulatory, digestive, nervous, and musculoskeletal system diseases, endocrine, metabolic, and eye diseases, or neoplasms. Exclusion criteria: Duplicate publication based on the same or overlapping study sample, and studies using only a single or few (<10) instrumental variables for nicotine dependence or smoking behavior or quantity.

**Table S1.** Single-nucleotide polymorphisms used as instrumental variables in the de novo

Mendelian randomization analyses of FinnGen data

| **Phenotype** | **SNP** | **Chr** | **EA** | **OA** | **Beta** | **SE** | ***P* value** | **F-statistic** |
| --- | --- | --- | --- | --- | --- | --- | --- | --- |
| Smoking initiation | rs2155646 | 11 | C | T | 0,0378 | 0,0026 | 9,44E-48 | 211 |
| Smoking initiation | rs11783093 | 8 | C | T | 0,0471 | 0,0035 | 2,07E-41 | 181 |
| Smoking initiation | rs6756212 | 2 | C | T | 0,0339 | 0,0026 | 3,49E-40 | 170 |
| Smoking initiation | rs1901477 | 2 | G | A | 0,0304 | 0,0026 | 2,07E-31 | 137 |
| Smoking initiation | rs118202 | 6 | G | T | 0,0367 | 0,0033 | 1,90E-29 | 124 |
| Smoking initiation | rs951740 | 1 | A | G | 0,0295 | 0,0026 | 3,82E-29 | 129 |
| Smoking initiation | rs1004787 | 2 | A | G | 0,0284 | 0,0026 | 1,11E-28 | 119 |
| Smoking initiation | rs10233018 | 7 | G | A | 0,0246 | 0,0025 | 4,77E-22 | 97 |
| Smoking initiation | rs13110073 | 4 | T | C | 0,0246 | 0,0026 | 3,24E-21 | 90 |
| Smoking initiation | rs12474587 | 2 | T | G | 0,0242 | 0,0026 | 4,83E-21 | 87 |
| Smoking initiation | rs6731872 | 2 | G | T | 0,0316 | 0,0034 | 5,35E-21 | 86 |
| Smoking initiation | rs1549979 | 3 | C | T | 0,0245 | 0,0026 | 8,80E-21 | 89 |
| Smoking initiation | rs13237637 | 7 | G | C | 0,0237 | 0,0025 | 1,54E-20 | 90 |
| Smoking initiation | rs281296 | 15 | A | G | 0,0247 | 0,0027 | 1,59E-20 | 84 |
| Smoking initiation | rs7921378 | 10 | G | C | 0,0233 | 0,0025 | 6,10E-20 | 87 |
| Smoking initiation | rs11594623 | 10 | C | T | 0,0274 | 0,0030 | 7,45E-20 | 83 |
| Smoking initiation | rs6265 | 11 | C | T | 0,0293 | 0,0033 | 2,81E-19 | 79 |
| Smoking initiation | rs10789369 | 1 | A | G | 0,0234 | 0,0026 | 3,39E-19 | 81 |
| Smoking initiation | rs12195240 | 6 | A | G | 0,0249 | 0,0028 | 1,08E-18 | 79 |
| Smoking initiation | rs1008078 | 1 | T | C | 0,0228 | 0,0026 | 1,63E-18 | 77 |
| Smoking initiation | rs6968380 | 7 | G | A | 0,0234 | 0,0027 | 1,05E-17 | 75 |
| Smoking initiation | rs12740789 | 1 | G | A | 0,0285 | 0,0033 | 1,18E-17 | 75 |
| Smoking initiation | rs4044321 | 5 | A | G | 0,0226 | 0,0027 | 1,75E-17 | 70 |
| Smoking initiation | rs2173019 | 5 | A | T | 0,0282 | 0,0033 | 2,98E-17 | 73 |
| Smoking initiation | rs12022778 | 1 | C | A | 0,0268 | 0,0032 | 3,18E-17 | 70 |
| Smoking initiation | rs359247 | 2 | T | A | 0,0220 | 0,0027 | 9,89E-17 | 66 |
| Smoking initiation | rs3909281 | 5 | G | T | 0,0211 | 0,0026 | 1,62E-16 | 66 |
| Smoking initiation | rs13109980 | 4 | G | A | 0,0222 | 0,0027 | 3,37E-16 | 68 |
| Smoking initiation | rs1445649 | 2 | C | T | 0,0206 | 0,0026 | 8,48E-16 | 63 |
| Smoking initiation | rs11128203 | 3 | A | T | 0,0204 | 0,0026 | 1,29E-15 | 62 |
| Smoking initiation | rs1139897 | 16 | G | A | 0,0241 | 0,0030 | 1,77E-15 | 65 |
| Smoking initiation | rs1937443 | 1 | G | C | 0,0204 | 0,0026 | 1,79E-15 | 62 |
| Smoking initiation | rs6986430 | 8 | T | C | 0,0243 | 0,0031 | 1,99E-15 | 61 |
| Smoking initiation | rs6497840 | 16 | A | G | 0,0228 | 0,0029 | 2,01E-15 | 62 |
| Smoking initiation | rs9987376 | 8 | T | G | 0,0205 | 0,0026 | 2,01E-15 | 62 |
| Smoking initiation | rs7809303 | 7 | G | A | 0,0214 | 0,0027 | 3,48E-15 | 63 |
| Smoking initiation | rs1373178 | 18 | T | G | 0,0203 | 0,0026 | 4,16E-15 | 61 |
| Smoking initiation | rs13261666 | 8 | G | T | 0,0200 | 0,0025 | 4,36E-15 | 64 |
| Smoking initiation | rs7072776 | 10 | A | G | 0,0220 | 0,0028 | 5,66E-15 | 62 |
| Smoking initiation | rs7188873 | 16 | G | A | 0,0203 | 0,0026 | 8,46E-15 | 61 |
| Smoking initiation | rs4818005 | 21 | G | A | 0,0204 | 0,0026 | 1,09E-14 | 62 |
| Smoking initiation | rs13437771 | 7 | A | G | 0,0271 | 0,0035 | 1,39E-14 | 60 |
| Smoking initiation | rs1108130 | 13 | A | T | 0,0239 | 0,0031 | 1,57E-14 | 59 |
| Smoking initiation | rs112725451 | 4 | T | C | 0,0261 | 0,0034 | 1,65E-14 | 59 |
| Smoking initiation | rs72780746 | 5 | T | C | 0,0258 | 0,0034 | 2,05E-14 | 58 |
| Smoking initiation | rs62106258 | 2 | T | C | 0,0455 | 0,0060 | 3,33E-14 | 58 |
| Smoking initiation | rs7743165 | 6 | G | T | 0,0193 | 0,0025 | 4,15E-14 | 60 |
| Smoking initiation | rs4674993 | 2 | A | G | 0,0240 | 0,0032 | 4,85E-14 | 56 |
| Smoking initiation | rs58400863 | 4 | G | A | 0,0202 | 0,0027 | 4,89E-14 | 56 |
| Smoking initiation | rs6438436 | 3 | T | C | 0,0247 | 0,0033 | 5,33E-14 | 56 |
| Smoking initiation | rs7333559 | 13 | G | A | 0,0232 | 0,0031 | 5,94E-14 | 56 |
| Smoking initiation | rs3850736 | 8 | G | C | 0,0191 | 0,0026 | 6,43E-14 | 54 |
| Smoking initiation | rs2063976 | 8 | C | T | 0,0202 | 0,0027 | 7,45E-14 | 56 |
| Smoking initiation | rs1514176 | 1 | G | A | 0,0193 | 0,0026 | 7,67E-14 | 55 |
| Smoking initiation | rs3820277 | 1 | G | T | 0,0188 | 0,0026 | 1,57E-13 | 52 |
| Smoking initiation | rs6890961 | 5 | C | T | 0,0193 | 0,0026 | 2,13E-13 | 55 |
| Smoking initiation | rs3847244 | 9 | T | C | 0,0187 | 0,0026 | 2,60E-13 | 52 |
| Smoking initiation | rs67777803 | 17 | G | T | 0,0246 | 0,0034 | 3,18E-13 | 52 |
| Smoking initiation | rs9302604 | 16 | G | A | 0,0187 | 0,0026 | 3,29E-13 | 52 |
| Smoking initiation | rs75919030 | 17 | T | C | 0,0210 | 0,0029 | 3,35E-13 | 52 |
| Smoking initiation | rs3172494 | 3 | G | T | 0,0291 | 0,0040 | 3,40E-13 | 53 |
| Smoking initiation | rs71592686 | 5 | C | T | 0,0207 | 0,0029 | 3,85E-13 | 51 |
| Smoking initiation | rs6936160 | 6 | T | C | 0,0201 | 0,0028 | 4,20E-13 | 52 |
| Smoking initiation | rs17692129 | 17 | T | C | 0,0196 | 0,0027 | 4,57E-13 | 53 |
| Smoking initiation | rs59537158 | 4 | T | C | 0,0225 | 0,0031 | 4,62E-13 | 53 |
| Smoking initiation | rs10279261 | 7 | G | A | 0,0189 | 0,0026 | 6,05E-13 | 53 |
| Smoking initiation | rs7657022 | 4 | G | A | 0,0183 | 0,0025 | 7,34E-13 | 54 |
| Smoking initiation | rs1150668 | 6 | T | G | 0,0185 | 0,0026 | 8,54E-13 | 51 |
| Smoking initiation | rs1931431 | 9 | C | G | 0,0182 | 0,0026 | 8,56E-13 | 49 |
| Smoking initiation | rs6050446 | 20 | G | A | 0,0544 | 0,0076 | 8,80E-13 | 51 |
| Smoking initiation | rs10966092 | 9 | T | C | 0,0205 | 0,0029 | 1,12E-12 | 50 |
| Smoking initiation | rs10060196 | 5 | A | C | 0,0183 | 0,0026 | 1,29E-12 | 50 |
| Smoking initiation | rs1381287 | 14 | T | C | 0,0180 | 0,0026 | 1,81E-12 | 48 |
| Smoking initiation | rs301807 | 1 | G | A | 0,0180 | 0,0026 | 2,50E-12 | 48 |
| Smoking initiation | rs9423279 | 10 | C | G | 0,0186 | 0,0027 | 3,06E-12 | 47 |
| Smoking initiation | rs12633090 | 3 | G | C | 0,0230 | 0,0033 | 3,16E-12 | 49 |
| Smoking initiation | rs2710634 | 2 | T | C | 0,0178 | 0,0026 | 3,36E-12 | 47 |
| Smoking initiation | rs9540731 | 13 | C | T | 0,0177 | 0,0025 | 3,42E-12 | 50 |
| Smoking initiation | rs9922607 | 16 | C | T | 0,0222 | 0,0032 | 3,42E-12 | 48 |
| Smoking initiation | rs9941217 | 16 | C | G | 0,0186 | 0,0027 | 3,50E-12 | 47 |
| Smoking initiation | rs72898831 | 18 | A | G | 0,0244 | 0,0035 | 4,14E-12 | 49 |
| Smoking initiation | rs12739243 | 1 | T | C | 0,0213 | 0,0031 | 4,45E-12 | 47 |
| Smoking initiation | rs72789626 | 5 | T | A | 0,0256 | 0,0037 | 5,13E-12 | 48 |
| Smoking initiation | rs4326350 | 8 | C | G | 0,0176 | 0,0026 | 5,16E-12 | 46 |
| Smoking initiation | rs540860 | 11 | G | A | 0,0176 | 0,0026 | 5,75E-12 | 46 |
| Smoking initiation | rs61886926 | 11 | C | T | 0,0179 | 0,0026 | 7,30E-12 | 47 |
| Smoking initiation | rs4790874 | 17 | T | C | 0,0174 | 0,0026 | 8,43E-12 | 45 |
| Smoking initiation | rs6750529 | 2 | T | C | 0,0199 | 0,0029 | 9,26E-12 | 47 |
| Smoking initiation | rs3218116 | 6 | C | T | 0,0198 | 0,0029 | 1,05E-11 | 47 |
| Smoking initiation | rs4543050 | 3 | T | A | 0,0222 | 0,0033 | 1,45E-11 | 45 |
| Smoking initiation | rs2901785 | 1 | G | A | 0,0173 | 0,0026 | 1,47E-11 | 44 |
| Smoking initiation | rs3811038 | 2 | C | T | 0,0191 | 0,0028 | 1,58E-11 | 47 |
| Smoking initiation | rs12878369 | 14 | A | C | 0,0174 | 0,0026 | 1,60E-11 | 45 |
| Smoking initiation | rs9538162 | 13 | C | T | 0,0174 | 0,0026 | 1,76E-11 | 45 |
| Smoking initiation | rs11766326 | 7 | T | C | 0,0175 | 0,0026 | 1,79E-11 | 45 |
| Smoking initiation | rs10805858 | 5 | T | A | 0,0181 | 0,0027 | 1,88E-11 | 45 |
| Smoking initiation | rs4877285 | 9 | G | A | 0,0181 | 0,0027 | 2,10E-11 | 45 |
| Smoking initiation | rs62098013 | 18 | A | G | 0,0177 | 0,0026 | 2,24E-11 | 46 |
| Smoking initiation | rs67050670 | 18 | A | G | 0,0203 | 0,0030 | 2,34E-11 | 46 |
| Smoking initiation | rs61533748 | 2 | C | T | 0,0174 | 0,0026 | 2,82E-11 | 45 |
| Smoking initiation | rs4674916 | 2 | C | A | 0,0180 | 0,0027 | 3,06E-11 | 44 |
| Smoking initiation | rs12918191 | 16 | A | G | 0,0197 | 0,0030 | 3,14E-11 | 43 |
| Smoking initiation | rs35375873 | 5 | G | C | 0,0270 | 0,0041 | 3,29E-11 | 43 |
| Smoking initiation | rs7192140 | 16 | T | C | 0,0169 | 0,0025 | 3,40E-11 | 46 |
| Smoking initiation | rs12130857 | 1 | G | A | 0,0180 | 0,0027 | 3,65E-11 | 44 |
| Smoking initiation | rs238896 | 11 | G | A | 0,0169 | 0,0025 | 3,65E-11 | 46 |
| Smoking initiation | rs10885480 | 10 | T | C | 0,0187 | 0,0028 | 3,83E-11 | 45 |
| Smoking initiation | rs10490159 | 2 | T | C | 0,0172 | 0,0026 | 3,86E-11 | 44 |
| Smoking initiation | rs7631379 | 3 | C | T | 0,0208 | 0,0032 | 3,94E-11 | 42 |
| Smoking initiation | rs76460663 | 11 | C | G | 0,0424 | 0,0064 | 4,15E-11 | 44 |
| Smoking initiation | rs7505855 | 18 | C | T | 0,0170 | 0,0026 | 5,31E-11 | 43 |
| Smoking initiation | rs1713676 | 11 | A | G | 0,0167 | 0,0026 | 5,38E-11 | 41 |
| Smoking initiation | rs876793 | 1 | T | C | 0,0179 | 0,0027 | 5,69E-11 | 44 |
| Smoking initiation | rs4785187 | 16 | A | G | 0,0200 | 0,0031 | 6,55E-11 | 42 |
| Smoking initiation | rs748832 | 3 | G | A | 0,0172 | 0,0026 | 6,60E-11 | 44 |
| Smoking initiation | rs61959481 | 13 | G | A | 0,0203 | 0,0031 | 7,95E-11 | 43 |
| Smoking initiation | rs2319545 | 3 | A | C | 0,0232 | 0,0036 | 8,30E-11 | 42 |
| Smoking initiation | rs71367544 | 18 | T | C | 0,0206 | 0,0032 | 8,54E-11 | 41 |
| Smoking initiation | rs1106363 | 11 | T | C | 0,0174 | 0,0027 | 9,20E-11 | 42 |
| Smoking initiation | rs113230003 | 19 | G | A | 0,0189 | 0,0029 | 1,05E-10 | 42 |
| Smoking initiation | rs12563365 | 1 | A | G | 0,0166 | 0,0026 | 1,05E-10 | 41 |
| Smoking initiation | rs3934797 | 4 | G | A | 0,0213 | 0,0033 | 1,12E-10 | 42 |
| Smoking initiation | rs11076962 | 16 | C | T | 0,0183 | 0,0028 | 1,20E-10 | 43 |
| Smoking initiation | rs28441558 | 17 | T | C | 0,0356 | 0,0055 | 1,24E-10 | 42 |
| Smoking initiation | rs34399632 | 2 | G | A | 0,0194 | 0,0030 | 1,46E-10 | 42 |
| Smoking initiation | rs62052916 | 16 | A | T | 0,0319 | 0,0050 | 1,62E-10 | 41 |
| Smoking initiation | rs6568832 | 6 | A | G | 0,0189 | 0,0030 | 1,74E-10 | 40 |
| Smoking initiation | rs8050598 | 16 | T | C | 0,0187 | 0,0029 | 1,76E-10 | 42 |
| Smoking initiation | rs1109480 | 12 | G | A | 0,0167 | 0,0026 | 1,84E-10 | 41 |
| Smoking initiation | rs2276825 | 3 | C | T | 0,0189 | 0,0030 | 1,89E-10 | 40 |
| Smoking initiation | rs2306866 | 3 | A | T | 0,0167 | 0,0026 | 1,89E-10 | 41 |
| Smoking initiation | rs13392222 | 2 | A | C | 0,0234 | 0,0037 | 1,93E-10 | 40 |
| Smoking initiation | rs2539706 | 2 | A | G | 0,0162 | 0,0026 | 1,95E-10 | 39 |
| Smoking initiation | rs329124 | 5 | A | G | 0,0164 | 0,0026 | 1,96E-10 | 40 |
| Smoking initiation | rs147052174 | 1 | T | G | 0,0623 | 0,0098 | 2,30E-10 | 40 |
| Smoking initiation | rs2010921 | 11 | A | G | 0,0174 | 0,0028 | 2,47E-10 | 39 |
| Smoking initiation | rs7929518 | 11 | G | A | 0,0192 | 0,0030 | 2,55E-10 | 41 |
| Smoking initiation | rs1799068 | 7 | T | G | 0,0166 | 0,0026 | 2,59E-10 | 41 |
| Smoking initiation | rs45444697 | 1 | G | C | 0,0197 | 0,0031 | 2,72E-10 | 40 |
| Smoking initiation | rs4822102 | 22 | C | T | 0,0165 | 0,0026 | 2,78E-10 | 40 |
| Smoking initiation | rs9545155 | 13 | T | C | 0,0161 | 0,0026 | 3,04E-10 | 38 |
| Smoking initiation | rs1714521 | 3 | A | C | 0,0163 | 0,0026 | 3,07E-10 | 39 |
| Smoking initiation | rs8005334 | 14 | G | T | 0,0167 | 0,0027 | 3,44E-10 | 38 |
| Smoking initiation | rs7943721 | 11 | G | A | 0,0212 | 0,0034 | 3,58E-10 | 39 |
| Smoking initiation | rs10945141 | 6 | A | G | 0,0181 | 0,0029 | 3,59E-10 | 39 |
| Smoking initiation | rs2925128 | 14 | T | C | 0,0168 | 0,0027 | 3,67E-10 | 39 |
| Smoking initiation | rs1385108 | 5 | T | C | 0,0187 | 0,0030 | 3,84E-10 | 39 |
| Smoking initiation | rs62180324 | 2 | G | A | 0,0195 | 0,0031 | 3,91E-10 | 40 |
| Smoking initiation | rs986714 | 5 | A | T | 0,0160 | 0,0026 | 4,13E-10 | 38 |
| Smoking initiation | rs4752018 | 10 | A | C | 0,0189 | 0,0030 | 4,42E-10 | 40 |
| Smoking initiation | rs10446419 | 3 | A | G | 0,0196 | 0,0031 | 5,05E-10 | 40 |
| Smoking initiation | rs2028269 | 5 | A | G | 0,0162 | 0,0026 | 5,19E-10 | 39 |
| Smoking initiation | rs1772572 | 13 | C | A | 0,0169 | 0,0027 | 5,62E-10 | 39 |
| Smoking initiation | rs4476253 | 18 | G | A | 0,0185 | 0,0030 | 5,78E-10 | 38 |
| Smoking initiation | rs11192347 | 10 | G | A | 0,0265 | 0,0043 | 6,15E-10 | 38 |
| Smoking initiation | rs1632941 | 6 | T | C | 0,0158 | 0,0026 | 6,67E-10 | 37 |
| Smoking initiation | rs2939756 | 11 | G | A | 0,0157 | 0,0026 | 7,45E-10 | 36 |
| Smoking initiation | rs62007780 | 15 | G | T | 0,0159 | 0,0026 | 7,48E-10 | 37 |
| Smoking initiation | rs4310804 | 15 | C | G | 0,0182 | 0,0030 | 7,55E-10 | 37 |
| Smoking initiation | rs42417 | 5 | T | C | 0,0169 | 0,0028 | 8,27E-10 | 36 |
| Smoking initiation | rs4886207 | 13 | T | C | 0,0162 | 0,0026 | 8,78E-10 | 39 |
| Smoking initiation | rs11057005 | 12 | A | G | 0,0157 | 0,0026 | 9,12E-10 | 36 |
| Smoking initiation | rs1173461 | 5 | T | C | 0,0166 | 0,0027 | 9,51E-10 | 38 |
| Smoking initiation | rs17165769 | 5 | G | A | 0,0159 | 0,0026 | 9,56E-10 | 37 |
| Smoking initiation | rs2959084 | 11 | A | G | 0,0171 | 0,0028 | 9,82E-10 | 37 |
| Smoking initiation | rs7802996 | 7 | C | T | 0,0209 | 0,0034 | 1,06E-09 | 38 |
| Smoking initiation | rs34553878 | 9 | G | A | 0,0247 | 0,0041 | 1,17E-09 | 36 |
| Smoking initiation | rs10858334 | 9 | G | C | 0,0229 | 0,0038 | 1,18E-09 | 36 |
| Smoking initiation | rs12855717 | 13 | T | C | 0,0155 | 0,0026 | 1,22E-09 | 36 |
| Smoking initiation | rs7920501 | 10 | T | A | 0,0155 | 0,0026 | 1,25E-09 | 36 |
| Smoking initiation | rs17229285 | 2 | C | T | 0,0155 | 0,0025 | 1,27E-09 | 38 |
| Smoking initiation | rs62137126 | 2 | A | G | 0,0237 | 0,0039 | 1,31E-09 | 37 |
| Smoking initiation | rs9288999 | 3 | A | G | 0,0174 | 0,0029 | 1,50E-09 | 36 |
| Smoking initiation | rs7969559 | 12 | A | G | 0,0170 | 0,0028 | 1,53E-09 | 37 |
| Smoking initiation | rs3810496 | 20 | C | T | 0,0159 | 0,0026 | 1,54E-09 | 37 |
| Smoking initiation | rs1759433 | 9 | A | G | 0,0154 | 0,0026 | 1,69E-09 | 35 |
| Smoking initiation | rs1449012 | 3 | C | T | 0,0154 | 0,0026 | 1,77E-09 | 35 |
| Smoking initiation | rs6874731 | 5 | G | T | 0,0153 | 0,0025 | 1,83E-09 | 37 |
| Smoking initiation | rs160631 | 6 | T | G | 0,0173 | 0,0029 | 1,87E-09 | 36 |
| Smoking initiation | rs8027457 | 15 | C | T | 0,0153 | 0,0025 | 1,88E-09 | 37 |
| Smoking initiation | rs12517438 | 5 | G | T | 0,0154 | 0,0026 | 1,89E-09 | 35 |
| Smoking initiation | rs13066050 | 3 | T | C | 0,0188 | 0,0031 | 1,93E-09 | 37 |
| Smoking initiation | rs13906 | 12 | C | T | 0,0245 | 0,0041 | 1,98E-09 | 36 |
| Smoking initiation | rs2289791 | 15 | G | T | 0,0177 | 0,0030 | 2,01E-09 | 35 |
| Smoking initiation | rs4837631 | 9 | C | T | 0,0154 | 0,0026 | 2,03E-09 | 35 |
| Smoking initiation | rs11611651 | 12 | A | G | 0,0271 | 0,0045 | 2,05E-09 | 36 |
| Smoking initiation | rs1160685 | 4 | G | C | 0,0153 | 0,0026 | 2,31E-09 | 35 |
| Smoking initiation | rs1863161 | 2 | A | G | 0,0153 | 0,0026 | 2,34E-09 | 35 |
| Smoking initiation | rs10698713 | 6 | G | A | 0,0335 | 0,0056 | 2,38E-09 | 36 |
| Smoking initiation | rs9627272 | 22 | G | C | 0,0155 | 0,0026 | 2,42E-09 | 36 |
| Smoking initiation | rs75674569 | 13 | G | A | 0,0253 | 0,0043 | 2,58E-09 | 35 |
| Smoking initiation | rs2378662 | 9 | A | G | 0,0152 | 0,0026 | 2,67E-09 | 34 |
| Smoking initiation | rs7026534 | 9 | T | G | 0,0166 | 0,0028 | 2,68E-09 | 35 |
| Smoking initiation | rs34940743 | 14 | G | A | 0,0159 | 0,0027 | 2,80E-09 | 35 |
| Smoking initiation | rs6011779 | 20 | C | T | 0,0192 | 0,0032 | 2,83E-09 | 36 |
| Smoking initiation | rs62246017 | 3 | G | A | 0,0162 | 0,0027 | 3,03E-09 | 36 |
| Smoking initiation | rs8103660 | 19 | C | T | 0,0158 | 0,0027 | 3,03E-09 | 34 |
| Smoking initiation | rs117657830 | 16 | A | G | 0,0378 | 0,0064 | 3,18E-09 | 35 |
| Smoking initiation | rs72790288 | 2 | G | A | 0,0455 | 0,0077 | 3,28E-09 | 35 |
| Smoking initiation | rs11642231 | 16 | G | A | 0,0156 | 0,0026 | 3,44E-09 | 36 |
| Smoking initiation | rs3800227 | 6 | G | A | 0,0172 | 0,0029 | 3,64E-09 | 35 |
| Smoking initiation | rs12714017 | 2 | C | T | 0,0154 | 0,0026 | 3,65E-09 | 35 |
| Smoking initiation | rs56902655 | 15 | T | G | 0,0219 | 0,0037 | 4,09E-09 | 35 |
| Smoking initiation | rs1889571 | 1 | G | T | 0,0222 | 0,0038 | 4,19E-09 | 34 |
| Smoking initiation | rs76608582 | 19 | C | A | 0,0345 | 0,0059 | 4,88E-09 | 34 |
| Smoking initiation | rs4788676 | 16 | T | C | 0,0177 | 0,0030 | 4,92E-09 | 35 |
| Smoking initiation | rs11162019 | 1 | C | T | 0,0155 | 0,0026 | 5,06E-09 | 36 |
| Smoking initiation | rs1737329 | 6 | G | C | 0,0170 | 0,0029 | 5,08E-09 | 34 |
| Smoking initiation | rs11872397 | 18 | G | A | 0,0171 | 0,0029 | 5,20E-09 | 35 |
| Smoking initiation | rs357304 | 2 | C | T | 0,0167 | 0,0029 | 5,40E-09 | 33 |
| Smoking initiation | rs3843905 | 5 | C | T | 0,0151 | 0,0026 | 5,41E-09 | 34 |
| Smoking initiation | rs73831818 | 3 | G | A | 0,0320 | 0,0055 | 5,46E-09 | 34 |
| Smoking initiation | rs1435479 | 4 | T | G | 0,0164 | 0,0028 | 5,68E-09 | 34 |
| Smoking initiation | rs10914684 | 1 | G | A | 0,0158 | 0,0027 | 6,32E-09 | 34 |
| Smoking initiation | rs4759229 | 12 | G | A | 0,0156 | 0,0027 | 6,53E-09 | 33 |
| Smoking initiation | rs1733760 | 10 | C | T | 0,0148 | 0,0025 | 6,70E-09 | 35 |
| Smoking initiation | rs7696257 | 4 | A | G | 0,0153 | 0,0026 | 6,78E-09 | 35 |
| Smoking initiation | rs4264267 | 13 | T | C | 0,0148 | 0,0026 | 6,82E-09 | 32 |
| Smoking initiation | rs1930371 | 9 | C | T | 0,0172 | 0,0030 | 7,09E-09 | 33 |
| Smoking initiation | rs586699 | 11 | G | A | 0,0148 | 0,0026 | 7,29E-09 | 32 |
| Smoking initiation | rs1050847 | 16 | C | T | 0,0148 | 0,0026 | 7,37E-09 | 32 |
| Smoking initiation | rs7598402 | 2 | C | G | 0,0147 | 0,0025 | 7,38E-09 | 35 |
| Smoking initiation | rs11956866 | 5 | T | G | 0,0148 | 0,0026 | 7,82E-09 | 32 |
| Smoking initiation | rs910912 | 20 | T | C | 0,0168 | 0,0029 | 7,82E-09 | 34 |
| Smoking initiation | rs2344976 | 17 | T | C | 0,0151 | 0,0026 | 7,98E-09 | 34 |
| Smoking initiation | rs2587507 | 17 | T | C | 0,0147 | 0,0025 | 8,69E-09 | 35 |
| Smoking initiation | rs16826827 | 2 | T | C | 0,0222 | 0,0039 | 9,17E-09 | 32 |
| Smoking initiation | rs290601 | 8 | T | C | 0,0163 | 0,0029 | 1,14E-08 | 32 |
| Smoking initiation | rs3740977 | 11 | C | T | 0,0195 | 0,0034 | 1,17E-08 | 33 |
| Smoking initiation | rs9841807 | 3 | T | C | 0,0163 | 0,0029 | 1,35E-08 | 32 |
| Smoking initiation | rs1291821 | 10 | G | A | 0,0145 | 0,0026 | 1,39E-08 | 31 |
| Smoking initiation | rs221988 | 3 | A | C | 0,0149 | 0,0026 | 1,43E-08 | 33 |
| Smoking initiation | rs6782116 | 3 | C | T | 0,0147 | 0,0026 | 1,46E-08 | 32 |
| Smoking initiation | rs2796793 | 10 | A | G | 0,0145 | 0,0026 | 1,55E-08 | 31 |
| Smoking initiation | rs9850597 | 3 | G | A | 0,0186 | 0,0033 | 1,65E-08 | 32 |
| Smoking initiation | rs1022376 | 2 | T | C | 0,0147 | 0,0026 | 1,66E-08 | 32 |
| Smoking initiation | rs7600835 | 2 | G | A | 0,0151 | 0,0027 | 1,80E-08 | 31 |
| Smoking initiation | rs10969352 | 9 | A | T | 0,0143 | 0,0025 | 1,82E-08 | 33 |
| Smoking initiation | rs16828799 | 3 | T | G | 0,0198 | 0,0035 | 1,83E-08 | 32 |
| Smoking initiation | rs55786907 | 13 | G | A | 0,0194 | 0,0035 | 1,84E-08 | 31 |
| Smoking initiation | rs7024924 | 9 | C | T | 0,0189 | 0,0034 | 1,90E-08 | 31 |
| Smoking initiation | rs12755632 | 1 | A | G | 0,0154 | 0,0027 | 1,93E-08 | 33 |
| Smoking initiation | rs62193862 | 2 | A | G | 0,0238 | 0,0042 | 1,99E-08 | 32 |
| Smoking initiation | rs2279829 | 3 | C | T | 0,0174 | 0,0031 | 2,05E-08 | 32 |
| Smoking initiation | rs17197663 | 13 | G | A | 0,0216 | 0,0039 | 2,06E-08 | 31 |
| Smoking initiation | rs2734390 | 3 | G | A | 0,0148 | 0,0026 | 2,09E-08 | 32 |
| Smoking initiation | rs62618693 | 11 | C | T | 0,0353 | 0,0063 | 2,09E-08 | 31 |
| Smoking initiation | rs17616642 | 2 | A | G | 0,0166 | 0,0030 | 2,10E-08 | 31 |
| Smoking initiation | rs71602617 | 4 | C | T | 0,0178 | 0,0032 | 2,10E-08 | 31 |
| Smoking initiation | rs34342129 | 18 | T | C | 0,0143 | 0,0025 | 2,13E-08 | 33 |
| Smoking initiation | rs1030015 | 7 | T | G | 0,0143 | 0,0026 | 2,15E-08 | 30 |
| Smoking initiation | rs1116690 | 4 | G | A | 0,0163 | 0,0029 | 2,16E-08 | 32 |
| Smoking initiation | rs35656245 | 1 | A | G | 0,0159 | 0,0029 | 2,23E-08 | 30 |
| Smoking initiation | rs60833441 | 15 | A | G | 0,0143 | 0,0026 | 2,28E-08 | 30 |
| Smoking initiation | rs13007361 | 2 | A | G | 0,0175 | 0,0031 | 2,29E-08 | 32 |
| Smoking initiation | rs61884449 | 11 | T | C | 0,0200 | 0,0036 | 2,32E-08 | 31 |
| Smoking initiation | rs10905461 | 10 | T | C | 0,0164 | 0,0029 | 2,36E-08 | 32 |
| Smoking initiation | rs6073075 | 20 | T | A | 0,0187 | 0,0034 | 2,44E-08 | 30 |
| Smoking initiation | rs73008357 | 6 | A | C | 0,0223 | 0,0040 | 2,44E-08 | 31 |
| Smoking initiation | rs9323328 | 14 | A | G | 0,0142 | 0,0026 | 2,55E-08 | 30 |
| Smoking initiation | rs6750107 | 2 | A | G | 0,0146 | 0,0026 | 2,60E-08 | 32 |
| Smoking initiation | rs8096225 | 18 | C | A | 0,0155 | 0,0028 | 2,63E-08 | 31 |
| Smoking initiation | rs56208390 | 2 | G | A | 0,0216 | 0,0039 | 2,68E-08 | 31 |
| Smoking initiation | rs1187820 | 3 | C | T | 0,0143 | 0,0026 | 2,69E-08 | 30 |
| Smoking initiation | rs11258417 | 10 | C | T | 0,0145 | 0,0026 | 2,71E-08 | 31 |
| Smoking initiation | rs7867822 | 9 | A | G | 0,0151 | 0,0027 | 2,76E-08 | 31 |
| Smoking initiation | rs1381775 | 11 | T | C | 0,0156 | 0,0028 | 2,79E-08 | 31 |
| Smoking initiation | rs3115418 | 2 | T | C | 0,0142 | 0,0026 | 2,79E-08 | 30 |
| Smoking initiation | rs10873871 | 1 | G | A | 0,0175 | 0,0031 | 2,82E-08 | 32 |
| Smoking initiation | rs1126757 | 19 | T | C | 0,0142 | 0,0026 | 2,92E-08 | 30 |
| Smoking initiation | rs4912332 | 1 | T | C | 0,0141 | 0,0025 | 2,94E-08 | 32 |
| Smoking initiation | rs925524 | 1 | G | A | 0,0156 | 0,0028 | 2,94E-08 | 31 |
| Smoking initiation | rs10935779 | 3 | C | T | 0,0143 | 0,0026 | 2,95E-08 | 30 |
| Smoking initiation | rs4727189 | 7 | C | T | 0,0149 | 0,0027 | 3,00E-08 | 30 |
| Smoking initiation | rs1927901 | 9 | T | C | 0,0142 | 0,0026 | 3,10E-08 | 30 |
| Smoking initiation | rs619087 | 6 | G | A | 0,0143 | 0,0026 | 3,10E-08 | 30 |
| Smoking initiation | rs17554906 | 6 | C | G | 0,0142 | 0,0026 | 3,14E-08 | 30 |
| Smoking initiation | rs11713899 | 3 | C | A | 0,0187 | 0,0034 | 3,15E-08 | 30 |
| Smoking initiation | rs359431 | 5 | C | T | 0,0142 | 0,0026 | 3,16E-08 | 30 |
| Smoking initiation | rs55913542 | 14 | T | G | 0,0186 | 0,0034 | 3,25E-08 | 30 |
| Smoking initiation | rs76841737 | 7 | C | G | 0,0231 | 0,0042 | 3,26E-08 | 30 |
| Smoking initiation | rs7640107 | 3 | C | T | 0,0142 | 0,0026 | 3,46E-08 | 30 |
| Smoking initiation | rs10953957 | 7 | A | G | 0,0144 | 0,0026 | 3,66E-08 | 31 |
| Smoking initiation | rs644740 | 11 | C | T | 0,0141 | 0,0026 | 3,67E-08 | 29 |
| Smoking initiation | rs11651955 | 17 | G | A | 0,0140 | 0,0025 | 3,74E-08 | 31 |
| Smoking initiation | rs6437769 | 3 | T | C | 0,0142 | 0,0026 | 3,74E-08 | 30 |
| Smoking initiation | rs1435672 | 15 | C | T | 0,0141 | 0,0026 | 3,82E-08 | 29 |
| Smoking initiation | rs9826984 | 3 | G | A | 0,0141 | 0,0026 | 3,87E-08 | 29 |
| Smoking initiation | rs9331343 | 6 | T | C | 0,0141 | 0,0026 | 3,90E-08 | 29 |
| Smoking initiation | rs77283305 | 7 | G | A | 0,0152 | 0,0028 | 3,91E-08 | 29 |
| Smoking initiation | rs11791671 | 9 | T | C | 0,0279 | 0,0051 | 4,24E-08 | 30 |
| Smoking initiation | rs7134009 | 12 | T | C | 0,0158 | 0,0029 | 4,30E-08 | 30 |
| Smoking initiation | rs62340589 | 4 | C | G | 0,0174 | 0,0032 | 4,31E-08 | 30 |
| Smoking initiation | rs7836565 | 8 | C | T | 0,0155 | 0,0028 | 4,36E-08 | 31 |
| Smoking initiation | rs11692435 | 2 | A | G | 0,0251 | 0,0046 | 4,47E-08 | 30 |
| Smoking initiation | rs10853981 | 19 | A | G | 0,0148 | 0,0027 | 4,88E-08 | 30 |
| Smoking initiation | rs4140932 | 4 | T | A | 0,0140 | 0,0026 | 4,89E-08 | 29 |
| Lifetime smoking | rs8042849 | 15 | C | T | 0,0280 | 0,0020 | 1,80E-39 | 196 |
| Lifetime smoking | rs113382419 | 9 | A | C | 0,0410 | 0,0030 | 3,00E-37 | 187 |
| Lifetime smoking | rs6011779 | 20 | C | T | 0,0280 | 0,0030 | 2,30E-27 | 87 |
| Lifetime smoking | rs9919670 | 11 | A | G | 0,0220 | 0,0020 | 7,60E-27 | 121 |
| Lifetime smoking | rs2890772 | 2 | T | G | 0,0200 | 0,0020 | 2,10E-22 | 100 |
| Lifetime smoking | rs35175834 | 15 | A | G | 0,0240 | 0,0020 | 4,60E-22 | 144 |
| Lifetime smoking | rs11783093 | 8 | C | T | 0,0230 | 0,0030 | 1,20E-16 | 59 |
| Lifetime smoking | rs11210229 | 1 | A | G | 0,0170 | 0,0020 | 2,00E-16 | 72 |
| Lifetime smoking | rs62155874 | 2 | G | A | 0,0240 | 0,0030 | 5,20E-16 | 64 |
| Lifetime smoking | rs10226228 | 7 | G | A | 0,0160 | 0,0020 | 2,00E-15 | 64 |
| Lifetime smoking | rs6119897 | 20 | A | G | 0,0180 | 0,0020 | 3,60E-15 | 81 |
| Lifetime smoking | rs2867112 | 2 | T | G | 0,0210 | 0,0030 | 4,80E-15 | 49 |
| Lifetime smoking | rs986391 | 5 | G | A | 0,0160 | 0,0020 | 9,40E-15 | 64 |
| Lifetime smoking | rs3742365 | 14 | C | T | 0,0160 | 0,0020 | 2,50E-14 | 64 |
| Lifetime smoking | rs2401924 | 7 | G | C | 0,0150 | 0,0020 | 2,70E-14 | 56 |
| Lifetime smoking | rs7807019 | 7 | G | A | 0,0150 | 0,0020 | 6,70E-14 | 56 |
| Lifetime smoking | rs549845 | 1 | G | A | 0,0160 | 0,0020 | 8,30E-14 | 64 |
| Lifetime smoking | rs10922907 | 1 | A | T | 0,0150 | 0,0020 | 3,00E-13 | 56 |
| Lifetime smoking | rs7569203 | 2 | C | A | 0,0160 | 0,0020 | 7,40E-13 | 64 |
| Lifetime smoking | rs17309874 | 11 | A | G | 0,0160 | 0,0020 | 9,70E-13 | 64 |
| Lifetime smoking | rs6778080 | 3 | T | C | 0,0160 | 0,0020 | 1,30E-12 | 64 |
| Lifetime smoking | rs8042134 | 15 | G | T | 0,0140 | 0,0020 | 1,30E-12 | 49 |
| Lifetime smoking | rs17576594 | 4 | G | A | 0,0160 | 0,0020 | 1,70E-12 | 64 |
| Lifetime smoking | rs7766610 | 6 | C | A | 0,0180 | 0,0030 | 2,20E-12 | 36 |
| Lifetime smoking | rs1922018 | 7 | C | T | 0,0140 | 0,0020 | 3,00E-12 | 49 |
| Lifetime smoking | rs7553348 | 1 | G | A | 0,0140 | 0,0020 | 5,20E-12 | 49 |
| Lifetime smoking | rs7528604 | 1 | G | A | 0,0140 | 0,0020 | 5,70E-12 | 49 |
| Lifetime smoking | rs329120 | 5 | C | T | 0,0140 | 0,0020 | 6,30E-12 | 49 |
| Lifetime smoking | rs12623702 | 2 | G | A | 0,0140 | 0,0020 | 7,70E-12 | 49 |
| Lifetime smoking | rs13296519 | 9 | T | G | 0,0140 | 0,0020 | 8,10E-12 | 49 |
| Lifetime smoking | rs6935954 | 6 | A | G | 0,0140 | 0,0020 | 8,20E-12 | 49 |
| Lifetime smoking | rs3896224 | 10 | A | G | 0,0140 | 0,0020 | 1,10E-11 | 49 |
| Lifetime smoking | rs4671357 | 2 | C | T | 0,0140 | 0,0020 | 1,10E-11 | 49 |
| Lifetime smoking | rs326341 | 3 | G | A | 0,0140 | 0,0020 | 1,20E-11 | 49 |
| Lifetime smoking | rs4391802 | 11 | A | G | 0,0150 | 0,0020 | 1,40E-11 | 56 |
| Lifetime smoking | rs72678864 | 4 | G | A | 0,0180 | 0,0030 | 1,60E-11 | 36 |
| Lifetime smoking | rs112282219 | 11 | A | G | 0,0330 | 0,0050 | 3,80E-11 | 44 |
| Lifetime smoking | rs10879871 | 12 | G | T | 0,0140 | 0,0020 | 5,00E-11 | 49 |
| Lifetime smoking | rs889398 | 16 | C | T | 0,0130 | 0,0020 | 6,30E-11 | 42 |
| Lifetime smoking | rs4473348 | 2 | T | A | 0,0150 | 0,0020 | 6,40E-11 | 56 |
| Lifetime smoking | rs1221148 | 9 | C | G | 0,0130 | 0,0020 | 7,30E-11 | 42 |
| Lifetime smoking | rs317021 | 4 | A | T | 0,0170 | 0,0030 | 1,10E-10 | 32 |
| Lifetime smoking | rs1933270 | 1 | T | G | 0,0130 | 0,0020 | 1,50E-10 | 42 |
| Lifetime smoking | rs8614 | 17 | A | C | 0,0170 | 0,0030 | 1,80E-10 | 32 |
| Lifetime smoking | rs11255908 | 10 | G | T | 0,0150 | 0,0020 | 2,30E-10 | 56 |
| Lifetime smoking | rs13153393 | 5 | G | A | 0,0200 | 0,0030 | 2,50E-10 | 44 |
| Lifetime smoking | rs2678670 | 2 | A | T | 0,0130 | 0,0020 | 3,10E-10 | 42 |
| Lifetime smoking | rs7333559 | 13 | G | A | 0,0150 | 0,0020 | 3,20E-10 | 56 |
| Lifetime smoking | rs76608582 | 19 | C | A | 0,0310 | 0,0050 | 3,20E-10 | 38 |
| Lifetime smoking | rs421983 | 3 | T | C | 0,0130 | 0,0020 | 3,30E-10 | 42 |
| Lifetime smoking | rs4543592 | 9 | C | T | 0,0120 | 0,0020 | 4,50E-10 | 36 |
| Lifetime smoking | rs11948770 | 5 | C | T | 0,0150 | 0,0020 | 4,90E-10 | 56 |
| Lifetime smoking | rs7039819 | 9 | G | A | 0,0130 | 0,0020 | 5,10E-10 | 42 |
| Lifetime smoking | rs10282292 | 7 | C | T | 0,0130 | 0,0020 | 5,90E-10 | 42 |
| Lifetime smoking | rs2838834 | 21 | T | C | 0,0130 | 0,0020 | 6,30E-10 | 42 |
| Lifetime smoking | rs624833 | 4 | T | G | 0,0130 | 0,0020 | 6,60E-10 | 42 |
| Lifetime smoking | rs62135536 | 2 | C | T | 0,0350 | 0,0060 | 8,00E-10 | 34 |
| Lifetime smoking | rs3811038 | 2 | C | T | 0,0140 | 0,0020 | 8,90E-10 | 49 |
| Lifetime smoking | rs359243 | 2 | C | T | 0,0130 | 0,0020 | 9,50E-10 | 42 |
| Lifetime smoking | rs35169606 | 8 | T | G | 0,0130 | 0,0020 | 1,20E-09 | 42 |
| Lifetime smoking | rs67596067 | 17 | A | G | 0,0130 | 0,0020 | 1,20E-09 | 42 |
| Lifetime smoking | rs6779302 | 3 | T | G | 0,0130 | 0,0020 | 1,20E-09 | 42 |
| Lifetime smoking | rs2675638 | 10 | G | A | 0,0120 | 0,0020 | 1,30E-09 | 36 |
| Lifetime smoking | rs75742406 | 11 | G | A | 0,0140 | 0,0020 | 1,30E-09 | 49 |
| Lifetime smoking | rs71367545 | 18 | A | G | 0,0150 | 0,0020 | 1,40E-09 | 56 |
| Lifetime smoking | rs71627581 | 5 | G | A | 0,0190 | 0,0030 | 1,60E-09 | 40 |
| Lifetime smoking | rs13016665 | 2 | A | C | 0,0120 | 0,0020 | 1,80E-09 | 36 |
| Lifetime smoking | rs369230 | 16 | T | G | 0,0130 | 0,0020 | 1,80E-09 | 42 |
| Lifetime smoking | rs10052591 | 5 | T | C | 0,0120 | 0,0020 | 2,10E-09 | 36 |
| Lifetime smoking | rs3769949 | 2 | A | T | 0,0120 | 0,0020 | 2,50E-09 | 36 |
| Lifetime smoking | rs7155595 | 14 | C | A | 0,0130 | 0,0020 | 2,50E-09 | 42 |
| Lifetime smoking | rs7077678 | 10 | C | T | 0,0120 | 0,0020 | 2,60E-09 | 36 |
| Lifetime smoking | rs860326 | 14 | C | T | 0,0120 | 0,0020 | 2,70E-09 | 36 |
| Lifetime smoking | rs12202536 | 6 | G | A | 0,0120 | 0,0020 | 2,80E-09 | 36 |
| Lifetime smoking | rs147412694 | 21 | A | G | 0,0170 | 0,0030 | 2,90E-09 | 32 |
| Lifetime smoking | rs4814873 | 20 | C | T | 0,0140 | 0,0020 | 2,90E-09 | 49 |
| Lifetime smoking | rs9842947 | 3 | T | C | 0,0130 | 0,0020 | 3,10E-09 | 42 |
| Lifetime smoking | rs12708665 | 16 | G | A | 0,0130 | 0,0020 | 3,50E-09 | 42 |
| Lifetime smoking | rs2894808 | 6 | A | T | 0,0220 | 0,0040 | 3,50E-09 | 30 |
| Lifetime smoking | rs202645 | 22 | G | A | 0,0150 | 0,0020 | 3,90E-09 | 56 |
| Lifetime smoking | rs62098013 | 18 | A | G | 0,0120 | 0,0020 | 4,10E-09 | 36 |
| Lifetime smoking | rs1246265 | 9 | C | T | 0,0130 | 0,0020 | 4,20E-09 | 42 |
| Lifetime smoking | rs4957528 | 5 | C | A | 0,0150 | 0,0020 | 4,20E-09 | 56 |
| Lifetime smoking | rs6598539 | 15 | C | T | 0,0120 | 0,0020 | 4,50E-09 | 36 |
| Lifetime smoking | rs13009008 | 2 | A | G | 0,0120 | 0,0020 | 4,60E-09 | 36 |
| Lifetime smoking | rs17553262 | 10 | C | A | 0,0180 | 0,0030 | 5,30E-09 | 36 |
| Lifetime smoking | rs7297175 | 12 | C | T | 0,0120 | 0,0020 | 6,60E-09 | 36 |
| Lifetime smoking | rs245774 | 5 | G | A | 0,0130 | 0,0020 | 7,40E-09 | 42 |
| Lifetime smoking | rs12481282 | 20 | C | G | 0,0130 | 0,0020 | 7,80E-09 | 42 |
| Lifetime smoking | rs6962772 | 7 | A | G | 0,0160 | 0,0030 | 7,80E-09 | 28 |
| Lifetime smoking | rs35343344 | 19 | C | A | 0,0130 | 0,0020 | 8,80E-09 | 42 |
| Lifetime smoking | rs6562474 | 13 | C | G | 0,0120 | 0,0020 | 1,00E-08 | 36 |
| Lifetime smoking | rs2062882 | 8 | A | G | 0,0120 | 0,0020 | 1,10E-08 | 36 |
| Lifetime smoking | rs775758 | 3 | A | T | 0,0120 | 0,0020 | 1,10E-08 | 36 |
| Lifetime smoking | rs34866095 | 11 | G | A | 0,0120 | 0,0020 | 1,20E-08 | 36 |
| Lifetime smoking | rs7519626 | 1 | C | T | 0,0120 | 0,0020 | 1,20E-08 | 36 |
| Lifetime smoking | rs9435340 | 1 | T | A | 0,0120 | 0,0020 | 1,20E-08 | 36 |
| Lifetime smoking | rs348809 | 20 | G | A | 0,0120 | 0,0020 | 1,30E-08 | 36 |
| Lifetime smoking | rs1050847 | 16 | C | T | 0,0110 | 0,0020 | 1,40E-08 | 30 |
| Lifetime smoking | rs4571506 | 5 | C | T | 0,0110 | 0,0020 | 1,50E-08 | 30 |
| Lifetime smoking | rs732083 | 17 | G | A | 0,0120 | 0,0020 | 1,50E-08 | 36 |
| Lifetime smoking | rs73220544 | 3 | C | A | 0,0160 | 0,0030 | 1,50E-08 | 28 |
| Lifetime smoking | rs6741228 | 2 | T | C | 0,0110 | 0,0020 | 1,60E-08 | 30 |
| Lifetime smoking | rs4949465 | 1 | C | T | 0,0170 | 0,0030 | 1,70E-08 | 32 |
| Lifetime smoking | rs62175972 | 2 | T | C | 0,0310 | 0,0060 | 1,70E-08 | 27 |
| Lifetime smoking | rs136233 | 22 | G | A | 0,0140 | 0,0030 | 1,80E-08 | 22 |
| Lifetime smoking | rs12831617 | 12 | T | C | 0,0130 | 0,0020 | 1,90E-08 | 42 |
| Lifetime smoking | rs11861214 | 16 | G | T | 0,0140 | 0,0020 | 2,00E-08 | 49 |
| Lifetime smoking | rs10823968 | 10 | A | T | 0,0120 | 0,0020 | 2,10E-08 | 36 |
| Lifetime smoking | rs10918701 | 1 | G | A | 0,0120 | 0,0020 | 2,10E-08 | 36 |
| Lifetime smoking | rs74086911 | 12 | G | A | 0,0210 | 0,0040 | 2,10E-08 | 28 |
| Lifetime smoking | rs28485305 | 15 | C | T | 0,0120 | 0,0020 | 2,60E-08 | 36 |
| Lifetime smoking | rs4731925 | 7 | T | C | 0,0120 | 0,0020 | 2,60E-08 | 36 |
| Lifetime smoking | rs1193237 | 1 | C | G | 0,0110 | 0,0020 | 2,80E-08 | 30 |
| Lifetime smoking | rs60952428 | 16 | T | C | 0,0190 | 0,0030 | 3,00E-08 | 40 |
| Lifetime smoking | rs12967855 | 18 | A | G | 0,0120 | 0,0020 | 3,10E-08 | 36 |
| Lifetime smoking | rs9904288 | 17 | T | C | 0,0120 | 0,0020 | 3,10E-08 | 36 |
| Lifetime smoking | rs2254710 | 6 | C | A | 0,0130 | 0,0020 | 3,50E-08 | 42 |
| Lifetime smoking | rs72674867 | 8 | A | T | 0,0130 | 0,0020 | 3,80E-08 | 42 |
| Lifetime smoking | rs1931263 | 1 | T | G | 0,0110 | 0,0020 | 4,00E-08 | 30 |
| Lifetime smoking | rs57611503 | 16 | G | A | 0,0110 | 0,0020 | 4,00E-08 | 30 |
| Lifetime smoking | rs61796681 | 4 | T | A | 0,0190 | 0,0040 | 4,20E-08 | 23 |
| Lifetime smoking | rs6957896 | 7 | T | C | 0,0110 | 0,0020 | 4,50E-08 | 30 |
| Lifetime smoking | rs2080870 | 5 | A | T | 0,0120 | 0,0020 | 4,90E-08 | 36 |

EA, effect allele; OA, other allele; SE, standard error; SNP, single-nucleotide polymorphism.

**Table S2.** Mendelian randomization studies included in the meta-analyses of genetic liability to **smoking initiation** in relation to circulatory system, digestive system, nervous system, musculoskeletal system, endocrine, metabolic and eye diseases, and neoplasms

| **Disease** | **Study** | **Cases** | **Noncases** | **SNPs** | **OR** | **LB** | **UB** | **First author, year** |
| --- | --- | --- | --- | --- | --- | --- | --- | --- |
| **CIRCULATORY SYSTEM** |  |  |  |  |  |  |  |  |
| Abdominal aortic aneurysm | UK Biobank | 1094 | 366549 | 361 | 1.74 | 1.33 | 2.26 | Larsson et al, 2020 |
| Aortic valve stenosis | UK Biobank | 2244 | 365399 | 361 | 1.22 | 1.01 | 1.46 | Larsson et al, 2020 |
| Atrial fibrillation | GWAS meta-analysis | 65446 | 522744 | 374 | 1.11 | 1.06 | 1.16 | Lu et al, 2021 |
| Atrial fibrillation | FinnGen | 28670 | 135821 | 297 | 1.23 | 1.10 | 1.36 | De novo MR analysis, 2022 |
| **Atrial fibrillation** | **Meta-analysis** | **94116** | **658565** |  | **1.13** | **1.08** | **1.18** |  |
| Coronary artery disease | UK Biobank | 29278 | 338365 | 361 | 1.36 | 1.27 | 1.45 | Larsson et al, 2020 |
| Coronary artery disease | CARDIoGRAMplusC4D | 60801 | 123504 | 361 | 1.26 | 1.18 | 1.35 | Larsson et al, 2020 |
| Coronary artery disease | FinnGen | 37854 | 222551 | 297 | 1.30 | 1.21 | 1.40 | De novo MR analysis, 2022 |
| **Coronary artery disease** | **Meta-analysis** | **127933** | **684420** |  | **1.31** | **1.26** | **1.36** |  |
| Deep vein thrombosis | UK Biobank | 9454 | 358189 | 361 | 1.29 | 1.17 | 1.42 | Larsson et al, 2020 |
| Deep vein thrombosis | FinnGen | 5632 | 225735 | 297 | 1.25 | 1.08 | 1.44 | De novo MR analysis, 2022 |
| **Deep vein thrombosis** | **Meta-analysis** | **15086** | **583924** |  | **1.28** | **1.18** | **1.38** |  |
| Heart failure | HERMES consortium | 47309 | 930014 | 359 | 1.28 | 1.18 | 1.38 | van Oort et al, 2020 |
| Heart failure | FinnGen | 15838 | 229946 | 297 | 1.21 | 1.11 | 1.33 | De novo MR analysis, 2022 |
| **Heart failure** | **Meta-analysis** | **63147** | **1159960** |  | **1.25** | **1.18** | **1.33** |  |
| Hypertension | UK Biobank | 125846 | 241797 | 361 | 1.21 | 1.14 | 1.28 | Larsson et al, 2020 |
| Hypertension | FinnGen | 53941 | 192355 | 297 | 1.20 | 1.10 | 1.30 | De novo MR analysis, 2022 |
| **Hypertension** | **Meta-analysis** | **179787** | **434152** |  | **1.21** | **1.15** | **1.27** |  |
| Intracerebral hemorrhage | UK Biobank | 1064 | 366579 | 361 | 1.19 | 0.91 | 1.55 | Larsson et al, 2020 |
| Intracerebral hemorrhage | ISGC | 1545 | 1481 | 326 | 1.13 | 0.81 | 1.58 | Larsson et al, 2019 |
| Intracerebral hemorrhage | FinnGen | 2008 | 238850 | 297 | 1.26 | 1.01 | 1.57 | De novo MR analysis, 2022 |
| **Intracerebral hemorrhage** | **Meta-analysis** | **4617** | **606910** |  | **1.21** | **1.04** | **1.41** |  |
| Ischemic stroke | UK Biobank | 4602 | 363041 | 361 | 1.30 | 1.15 | 1.48 | Larsson et al, 2020 |
| Ischemic stroke | MEGASTROKE | 34217 | 404630 | 372 | 1.24 | 1.17 | 1.33 | Larsson et al, 2019 |
| Ischemic stroke | FinnGen | 12948 | 240124 | 297 | 1.12 | 1.02 | 1.24 | De novo MR analysis, 2022 |
| **Ischemic stroke** | **Meta-analysis** | **51767** | **1007795** |  | **1.21** | **1.16** | **1.28** |  |
| Peripheral artery disease | UK Biobank | 3415 | 364228 | 361 | 1.81 | 1.55 | 2.11 | Larsson et al, 2020 |
| Peripheral artery disease | FinnGen | 9021 | 244907 | 297 | 1.63 | 1.45 | 1.82 | De novo MR analysis, 2022 |
| **Peripheral artery disease** | **Meta-analysis** | **12436** | **609135** |  | **1.69** | **1.54** | **1.85** |  |
| Pulmonary embolism | UK Biobank | 6148 | 361495 | 361 | 1.32 | 1.17 | 1.49 | Larsson et al, 2020 |
| Pulmonary embolism | FinnGen | 5130 | 254771 | 297 | 1.13 | 0.99 | 1.30 | De novo MR analysis, 2022 |
| **Pulmonary embolism** | **Meta-analysis** | **11278** | **616266** |  | **1.23** | **1.13** | **1.35** |  |
| Subarachnoid hemorrhage | UK Biobank | 1084 | 366559 | 361 | 1.42 | 1.09 | 1.45 | Larsson et al, 2020 |
| Subarachnoid hemorrhage | ISGC | 4196 | 59544 | 235 | 1.62 | 1.24 | 2.13 | Karhunen et al, 2021 |
| Subarachnoid hemorrhage | FinnGen | 1620 | 238926 | 297 | 1.26 | 0.98 | 1.63 | De novo MR analysis, 2022 |
| **Subarachnoid hemorrhage** | **Meta-analysis** | **6900** | **665029** |  | **1.42** | **1.27** | **1.59** |  |
| Thoracic aortic aneurysm | UK Biobank | 347 | 367296 | 361 | 1.07 | 0.67 | 1.68 | Larsson et al, 2020 |
| Transient ischemic attack | UK Biobank | 3962 | 363681 | 361 | 1.30 | 1.13 | 1.51 | Larsson et al, 2020 |
| Transient ischemic attack | FinnGen | 10830 | 240124 | 297 | 1.15 | 1.03 | 1.28 | De novo MR analysis, 2022 |
| **Transient ischemic attack** | **Meta-analysis** | **14792** | **603805** |  | **1.20** | **1.10** | **1.31** |  |
| Varicose veins | UK Biobank | 8763 | 352431 | 312 | 1.15 | 1.02 | 1.29 | Yuan et al, 2021 |
| Varicose veins | FinnGen | 20425 | 225735 | 297 | 1.11 | 0.99 | 1.23 | Yuan et al, 2021 |
| **Varicose veins** | **Meta-analysis** | **29188** | **578166** |  | **1.13** | **1.04** | **1.22** |  |
| Venous thromboembolism | UK Biobank | 14097 | 353546 | 361 | 1.29 | 1.18 | 1.40 | Larsson et al, 2020 |
| Venous thromboembolism | Million Veteran Program | 8929 | 181337 | 361 | 1.09 | 0.98 | 1.21 | Larsson et al, 2020 |
| Venous thromboembolism | FinnGen | 11288 | 249117 | 297 | 1.19 | 1.08 | 1.32 | De novo MR analysis, 2022 |
| **Venous thromboembolism** | **Meta-analysis** | **34314** | **784000** |  | **1.20** | **1.14** | **1.27** |  |
| **DIGESTIVE SYSTEM** |  |  |  |  |  |  |  |  |
| Crohn's disease | GWAS meta-analysis | 12194 | 25042 | 325 | 1.14 | 0.99 | 1.31 | Jones et al, 2020 |
| Crohn's disease | FinnGen | 810 | 249705 | 297 | 1.26 | 0.87 | 1.82 | De novo MR analysis, 2022 |
| **Crohn's disease** | **Meta-analysis** | **13004** | **274747** |  | **1.15** | **1.01** | **1.32** |  |
| Diverticular disease | UK Biobank | 12662 | 348532 | 312 | 1.27 | 1.15 | 1.40 | Yuan & Larsson, 2021 |
| Diverticular disease | FinnGen | 10978 | 149001 | 297 | 1.14 | 1.02 | 1.27 | Yuan & Larsson, 2021 |
| **Diverticular disease** | **Meta-analysis** | **23640** | **497533** |  | **1.21** | **1.12** | **1.30** |  |
| Gallstone disease | UK Biobank | 10520 | 350674 | 309 | 1.40 | 1.27 | 1.55 | Yuan et al, 2021 |
| Gallstone disease | FinnGen | 11675 | 121348 | 295 | 1.09 | 0.98 | 1.22 | Yuan et al, 2021 |
| **Gallstone disease** | **Meta-analysis** | **22195** | **472022** |  | **1.25** | **1.16** | **1.35** |  |
| GERD | UK Biobank & Qskin Sun & Health | 71522 | 261079 | 202 | 1.41 | 1.31 | 1.52 | Yuan & Larsson 2022 |
| GERD | FinnGen | 16043 | 225008 | 297 | 1.26 | 1.14 | 1.38 | De novo MR analysis, 2022 |
| **GERD** | **Meta-analysis** | **87565** | **486087** |  | **1.35** | **1.27** | **1.43** |  |
| Pancreatitis, acute | UK Biobank | 1292 | 359902 | 366 | 1.42 | 1.10 | 1.82 | Yuan et al, 2021 |
| Pancreatitis, acute | FinnGen | 1762 | 121348 | 352 | 1.66 | 1.35 | 2.05 | Yuan et al, 2021 |
| **Pancreatitis, acute** | **Meta-analysis** | **3054** | **481250** |  | **1.56** | **1.33** | **1.83** |  |
| Pancreatitis, chronic | UK Biobank | 246 | 360948 | 366 | 2.04 | 1.19 | 3.5 | Yuan et al, 2021 |
| Pancreatitis, chronic | FinnGen | 914 | 121348 | 352 | 1.81 | 1.34 | 2.45 | Yuan et al, 2021 |
| **Pancreatitis, chronic** | **Meta-analysis** | **1160** | **482296** |  | **1.86** | **1.43** | **2.43** |  |
| **Periodontitis, chronic** | **FinnGen** | **14631** | **245774** | **297** | **1.47** | **1.35** | **1.61** | **De novo MR analysis, 2022** |
| Ulcerative colitis | GWAS meta-analysis | 12366 | 25042 | 325 | 0.98 | 0.86 | 1.11 | Jones et al, 2020 |
| Ulcerative colitis | FinnGen | 2768 | 249705 | 297 | 1.13 | 0.92 | 1.38 | De novo MR analysis, 2022 |
| **Ulcerative colitis** | **Meta-analysis** | **15134** | **274747** |  | **1.02** | **0.92** | **1.14** |  |
| **NERVOUS SYSTEM** |  |  |  |  |  |  |  |  |
| Alzheimer's disease | PGC | 71880 | 383378 | 74 | 0.90 | 0.76 | 1.06 | Yang et al, 2020 |
| Alzheimer's disease | FinnGen | 7329 | 252879 | 297 | 1.25 | 1.09 | 1.43 | De novo MR analysis, 2022 |
| **Alzheimer's disease** | **Meta-analysis** | **79209** | **636257** |  | **1.10** | **0.99** | **1.22** |  |
| ALS | GWAS meta-analysis | 20806 | 59804 | 301 | 1.10 | 1.00 | 1.23 | Opie-Martin et al, 2020 |
| ALS | FinnGen | 262 | 131274 | 297 | 1.28 | 0.71 | 2.30 | De novo MR analysis, 2022 |
| **ALS** | **Meta-analysis** | **21068** | **191078** |  | **1.11** | **1.00** | **1.22** |  |
| Epilepsy | UK Biobank | 901 | 395209 | 295 | 1.48 | 1.12 | 1.97 | Yuan et al, 2021 |
| Epilepsy | FinnGen | 4588 | 144780 | 295 | 1.19 | 1.02 | 1.38 | Yuan et al, 2021 |
| **Epilepsy** | **Meta-analysis** | **5489** | **539989** |  | **1.25** | **1.09** | **1.43** |  |
| Multiple sclerosis | IMSGC | 14802 | 26703 | 297 | 1.06 | 0.92 | 1.21 | Vandebergh & Goris 2020 |
| Multiple sclerosis | FinnGen | 1364 | 258340 | 297 | 0.95 | 0.71 | 1.27 | De novo MR analysis, 2022 |
| **Multiple sclerosis** | **Meta-analysis** | **16166** | **285043** |  | **1.04** | **0.92** | **1.18** |  |
| Parkinson's disease | IPDGC | 36752 | 929806 | 365 | 0.75 | 0.62 | 0.91 | Sieurin et al, 2021 |
| Parkinson's disease | FinnGen | 2496 | 257909 | 297 | 0.92 | 0.75 | 1.12 | De novo MR analysis, 2022 |
| **Parkinson's disease** | **Meta-analysis** | **39248** | **1187715** |  | **0.83** | **0.72** | **0.95** |  |
| **MUSCULOSKELETAL SYSTEM** |  |  |  |  |  |  |  |  |
| Fracture | GEFOS | 53184 | 373611 | 377 | 1.09 | 1.04 | 1.15 | Yuan et al, 2019 |
| Fracture | FinnGen | 4819 | 251409 | 297 | 1.12 | 0.96 | 1.31 | De novo MR analysis, 2022 |
| **Fracture** | **Meta-analysis** | **58003** | **625020** |  | **1.09** | **1.04** | **1.15** |  |
| Gout | GUGC | 2115 | 67259 | 259 | 1.10 | 0.92 | 1.32 | De novo MR analysis, 2022 |
| Gout | FinnGen | 4502 | 241230 | 297 | 1.11 | 0.95 | 1.29 | De novo MR analysis, 2022 |
| **Gout** | **Meta-analysis** | **6617** | **308489** |  | **1.11** | **0.98** | **1.24** |  |
| Osteoarthritis | UK Biobank | 39427 | 378169 | 314 | 1.30 | 1.21 | 1.39 | De novo MR analysis, 2022 |
| Osteoarthritis | FinnGen | 45467 | 173414 | 297 | 1.32 | 1.22 | 1.42 | De novo MR analysis, 2022 |
| **Osteoarthritis** | **Meta-analysis** | **84894** | **551583** |  | **1.31** | **1.24** | **1.38** |  |
| Rheumatoid arthritis | GWAS meta-analysis | 14361 | 43923 | 367 | 1.32 | 1.15 | 1.52 | Qian et al, 2020 |
| Rheumatoid arthritis | FinnGen | 8428 | 251977 | 297 | 1.40 | 1.23 | 1.60 | De novo MR analysis, 2022 |
| **Rheumatoid arthritis** | **Meta-analysis** | **22789** | **295900** |  | **1.36** | **1.24** | **1.50** |  |
| **ENDOCRINE & METABOLIC** |  |  |  |  |  |  |  |  |
| Polycystic ovary syndrome | GWAS meta-analysis | 4138 | 20129 | 360 | 1.38 | 1.12 | 1.69 | Tao et al, 2021 |
| Polycystic ovary syndrome | FinnGen | 797 | 140558 | 297 | 1.29 | 0.91 | 1.82 | De novo MR analysis, 2022 |
| **Polycystic ovary syndrome** | **Meta-analysis** | **4935** | **160687** |  | **1.36** | **1.14** | **1.62** |  |
| Type 2 diabetes | DIAGRAM consortium | 74124 | 824006 | 377 | 1.08 | 1.02 | 1.15 | Yuan & Larsson, 2020 |
| Type 2 diabetes | FinnGen | 37002 | 215160 | 297 | 1.23 | 1.13 | 1.34 | De novo MR analysis, 2022 |
| **Type 2 diabetes** | **Meta-analysis** | **111126** | **1039166** |  | **1.13** | **1.07** | **1.18** |  |
| **EYE DISEASES** |  |  |  |  |  |  |  |  |
| AMD | IAMDGC | 16144 | 17832 | 336 | 1.26 | 1.13 | 1.40 | Kuan et al, 2021 |
| AMD | FinnGen | 4645 | 243951 | 297 | 1.09 | 0.93 | 1.28 | De novo MR analysis, 2022 |
| **AMD** | **Meta-analysis** | **20789** | **261783** |  | **1.20** | **1.10** | **1.32** |  |
| Primary OAG | GWAS meta-analysis | 133492 | 90939 | 314 | 0.93 | 0.86 | 1.02 | De novo MR analysis, 2022 |
| Primary OAG | FinnGen | 4164 | 249920 | 297 | 1.04 | 0.88 | 1.22 | De novo MR analysis, 2022 |
| **Primary OAG** | **Meta-analysis** | **137656** | **340859** |  | **0.95** | **0.88** | **1.03** |  |
| Senile cataract | UK Biobank | 26489 | 509767 | 312 | 1.20 | 1.06 | 1.37 | Yuan et al, 2022 |
| Senile cataract | FinnGen | 32692 | 224812 | 297 | 1.18 | 1.06 | 1.31 | Yuan et al, 2022 |
| **Senile cataract** | **Meta-analysis** | **59181** | **734579** |  | **1.19** | **1.10** | **1.29** |  |
| **NEOPLASMS** |  |  |  |  |  |  |  |  |
| Bladder cancer | UK Biobank | 2588 | 365055 | 361 | 1.40 | 1.18 | 1.66 | Larsson et al, 2020 |
| Bladder cancer | FinnGen | 1701 | 258704 | 297 | 1.36 | 1.07 | 1.72 | De novo MR analysis, 2022 |
| **Bladder cancer** | **Meta-analysis** | **4289** | **623759** |  | **1.39** | **1.21** | **1.59** |  |
| Brain cancer | UK Biobank | 810 | 366833 | 361 | 0.99 | 0.72 | 1.35 | Larsson et al, 2020 |
| Brain cancer | FinnGen | 615 | 259790 | 297 | 0.80 | 0.54 | 1.18 | De novo MR analysis, 2022 |
| **Brain cancer** | **Meta-analysis** | **1425** | **626623** |  | **0.91** | **0.71** | **1.16** |  |
| Breast cancer | UK Biobank | 13666 | 185172 | 361 | 0.97 | 0.90 | 1.06 | Larsson et al, 2020 |
| Breast cancer | BCAC | 122977 | 105974 | 351 | 1.05 | 0.99 | 1.12 | Larsson et al, 2020 |
| Breast cancer | FinnGen | 11573 | 135488 | 297 | 1.05 | 0.94 | 1.17 | De novo MR analysis, 2022 |
| **Breast cancer** | **Meta-analysis** | **148216** | **426634** |  | **1.03** | **0.98** | **1.07** |  |
| Cervical cancer | UK Biobank | 1928 | 196910 | 361 | 1.55 | 1.27 | 1.88 | Larsson et al, 2020 |
| Cervical cancer | FinnGen | 2229 | 144832 | 297 | 1.14 | 0.92 | 1.42 | De novo MR analysis, 2022 |
| **Cervical cancer** | **Meta-analysis** | **4157** | **341742** |  | **1.35** | **1.17** | **1.56** |  |
| Colorectal cancer | UK Biobank | 5486 | 362157 | 361 | 1.10 | 0.97 | 1.26 | Larsson et al, 2020 |
| Colorectal cancer | CORECT and GECCO | 52775 | 45940 | 112 | 0.97 | 0.86 | 1.10 | Dimou et al, 2021 |
| Colorectal cancer | FinnGen | 4401 | 256004 | 297 | 0.94 | 0.80 | 1.10 | De novo MR analysis, 2022 |
| **Colorectal cancer** | **Meta-analysis** | **62662** | **664101** |  | **1.01** | **0.93** | **1.09** |  |
| Esophageal cancer | UK Biobank | 843 | 366800 | 361 | 1.83 | 1.34 | 2.49 | Larsson et al, 2020 |
| Esophageal cancer | FinnGen | 358 | 260047 | 190 | 1.59 | 0.97 | 2.62 | De novo MR analysis, 2022 |
| **Esophageal cancer** | **Meta-analysis** | **1201** | **626847** |  | **1.76** | **1.35** | **2.29** |  |
| Head/neck cancer | UK Biobank | 1615 | 366028 | 361 | 1.40 | 1.13 | 1.74 | Larsson et al, 2020 |
| Head/neck cancer | GAME-ON GWAS | 6034 | 6585 | 176 | 2.50 | 1.59 | 3.91 | Gormley et al, 2020 |
| Head/neck cancer | FinnGen | 208 | 260197 | 297 | 1.37 | 0.71 | 2.64 | De novo MR analysis, 2022 |
| **Head/neck cancer** | **Meta-analysis** | **7857** | **632810** |  | **1.54** | **1.28** | **1.86** |  |
| Kidney cancer | UK Biobank | 1310 | 366333 | 361 | 1.28 | 0.99 | 1.66 | Larsson et al, 2020 |
| Kidney cancer | FinnGen | 1393 | 259012 | 297 | 1.43 | 1.11 | 1.85 | De novo MR analysis, 2022 |
| **Kidney cancer** | **Meta-analysis** | **2703** | **625345** |  | **1.35** | **1.13** | **1.62** |  |
| Leukemia | UK Biobank | 1403 | 366240 | 361 | 1.07 | 0.84 | 1.35 | Larsson et al, 2020 |
| Leukemia, lymphoid | FinnGen | 808 | 259597 | 297 | 0.81 | 0.58 | 1.14 | De novo MR analysis, 2022 |
| Leukemia, myeloid | FinnGen | 340 | 260065 | 297 | 1.68 | 1.01 | 2.82 | De novo MR analysis, 2022 |
| Lung cancer | UK Biobank | 2838 | 364805 | 361 | 2.26 | 1.92 | 2.65 | Larsson et al, 2020 |
| Lung cancer | ILCCO | 11348 | 15861 | 346 | 1.80 | 1.59 | 2.03 | Larsson et al, 2020 |
| Lung cancer | FinnGen | 3061 | 257344 | 297 | 1.59 | 1.30 | 1.94 | De novo MR analysis, 2022 |
| **Lung cancer** | **Meta-analysis** | **17247** | **638010** |  | **1.88** | **1.72** | **2.05** |  |
| Melanoma | UK Biobank | 4869 | 362774 | 361 | 0.92 | 0.80 | 1.06 | Larsson et al, 2020 |
| Melanoma | FinnGen | 143 | 260262 | 297 | 1.64 | 0.73 | 3.68 | De novo MR analysis, 2022 |
| **Melanoma** | **Meta-analysis** | **5012** | **623036** |  | **0.94** | **0.82** | **1.08** |  |
| Multiple myeloma | UK Biobank | 656 | 366987 | 361 | 0.99 | 0.70 | 1.40 | Larsson et al, 2020 |
| Multiple myeloma | FinnGen | 693 | 259712 | 297 | 0.77 | 0.54 | 1.11 | De novo MR analysis, 2022 |
| **Multiple myeloma** | **Meta-analysis** | **1349** | **626699** |  | **0.88** | **0.68** | **1.13** |  |
| Non-Hodgkin lymphoma | UK Biobank | 2296 | 365347 | 361 | 0.87 | 0.73 | 1.05 | Larsson et al, 2020 |
| Non-Hodgkin lymphoma | FinnGen | 643 | 259762 | 297 | 0.99 | 0.68 | 1.46 | De novo MR analysis, 2022 |
| **Non-Hodgkin lymphoma** | **Meta-analysis** | **2939** | **625109** |  | **0.89** | **0.76** | **1.05** |  |
| Ovarian cancer | UK Biobank | 1520 | 197318 | 361 | 1.06 | 0.85 | 1.32 | Larsson et al, 2020 |
| Ovarian cancer | OCAC | 25509 | 40941 | 351 | 1.04 | 0.95 | 1.14 | Larsson et al, 2020 |
| Ovarian cancer | FinnGen | 1041 | 146020 | 297 | 0.86 | 0.64 | 1.15 | De novo MR analysis, 2022 |
| **Ovarian cancer** | **Meta-analysis** | **28070** | **384279** |  | **1.03** | **0.95** | **1.11** |  |
| Pancreatic cancer | UK Biobank | 1264 | 366379 | 361 | 1.15 | 0.90 | 1.47 | Larsson et al, 2020 |
| Pancreatic cancer | PanScan & PanC4 | 8769 | 7055 | 188 | 1.04 | 0.87 | 1.25 | Lu et al, 2020 |
| Pancreatic cancer | FinnGen | 881 | 259524 | 297 | 0.92 | 0.67 | 1.27 | De novo MR analysis, 2022 |
| **Pancreatic cancer** | **Meta-analysis** | **10914** | **632958** |  | **1.05** | **0.92** | **1.20** |  |
| Prostate cancer | UK Biobank | 7872 | 160876 | 361 | 0.90 | 0.80 | 1.02 | Larsson et al, 2020 |
| Prostate cancer | PRACTICAL | 79148 | 61106 | 350 | 0.90 | 0.83 | 0.98 | Larsson et al, 2020 |
| Prostate cancer | FinnGen | 8709 | 104635 | 297 | 0.95 | 0.83 | 1.08 | De novo MR analysis, 2022 |
| **Prostate cancer** | **Meta-analysis** | **95729** | **326617** |  | **0.91** | **0.86** | **0.97** |  |
| Stomach cancer | UK Biobank | 736 | 366907 | 361 | 1.46 | 1.05 | 2.03 | Larsson et al, 2020 |
| Stomach cancer | FinnGen | 889 | 259516 | 297 | 1.08 | 0.78 | 1.49 | De novo MR analysis, 2022 |
| **Stomach cancer** | **Meta-analysis** | **1625** | **626423** |  | **1.25** | **0.99** | **1.58** |  |
| Testicular cancer | UK Biobank | 735 | 168013 | 361 | 1.09 | 0.77 | 1.54 | Larsson et al, 2020 |
| Testicular cancer | FinnGen | 268 | 113076 | 297 | 0.76 | 0.43 | 1.35 | De novo MR analysis, 2022 |
| **Testicular cancer** | **Meta-analysis** | **1003** | **281089** |  | **0.99** | **0.74** | **1.33** |  |
| Uterine cancer | UK Biobank | 1931 | 196907 | 361 | 1.11 | 0.91 | 1.37 | Larsson et al, 2020 |
| Uterine cancer | FinnGen | 1430 | 145631 | 297 | 0.91 | 0.70 | 1.19 | De novo MR analysis, 2022 |
| **Uterine cancer** | **Meta-analysis** | **3361** | **342538** |  | **1.03** | **0.88** | **1.21** |  |

ALS, amyotrophic lateral sclerosis; BCAC, Breast Cancer Association Consortium; CARDIoGRAMplusC4D, Coronary ARtery DIsease Genome-wide Replication and Meta-analysis plus The Coronary Artery Disease Genetics consortium; CORECT, Colorectal Transdisciplary Study; DIAGRAM, DIAbetes Genetics Replication And Meta-analysis Consortium; GAME-ON, Genetic Associations and Mechanisms in Oncology; GECCO, Genetics and Epidemiology of Colorectal Cancer Consortium; GEFOS, GEnetic Factors for OSteoporosis Consortium; GERD, gastroesophageal reflux disease; GUGC, Global Urate Genetics Consortium; GWAS, genome-wide association; study; HERMES, Heart failure molecular epidemiology for therapeutic targets; ILCCO, International Lung Cancer Consortium; IMSGC, International Multiple Sclerosis Genetics Consortium; IPDGC, International Parkinson Disease Genomics Consortium; IAMDGC, International Age-related Macular Degeneration Genomics Consortium; ISGC, International Stroke Genetics Consortium; LB, lower bound of the 95% confidence interval; OCAC, Ovarian Cancer Association Consortium; OR, odds ratio; PGC, Psychiatric Genomics Consortium; PRACTICAL, Prostate Cancer Association Group to Investigate Cancer Associated Alterations in the Genome; UB, upper bound of the 95% confidence interval.

**Table S3.** Mendelian randomization studies included in the meta-analyses of genetic liability to **lifetime smoking** in relation to circulatory system, digestive system, nervous system, musculoskeletal system, endocrine, metabolic and eye diseases, and neoplasms

| **Disease** | **Study** | **Cases** | **Noncases** | **SNPs** | **OR** | **LB** | **UB** | **First author, year** |
| --- | --- | --- | --- | --- | --- | --- | --- | --- |
| **CIRCULATORY SYSTEM** |  |  |  |  |  |  |  |  |
| Abdominal aortic aneurysm | UK Biobank | 1094 | 366549 | 126 | 5.51 | 3.14 | 9.68 | Larsson et al, 2020 |
| Aortic valve stenosis | UK Biobank | 2244 | 365399 | 126 | 1.64 | 1.06 | 2.54 | Larsson et al, 2020 |
| Atrial fibrillation | UK Biobank | 16945 | 350698 | 126 | 1.47 | 1.23 | 1.74 | Larsson et al, 2020 |
| Atrial fibrillation | AF Consortium | 22346 | 132086 | 126 | 1.04 | 0.86 | 1.27 | Larsson et al, 2020 |
| Atrial fibrillation | FinnGen | 28670 | 135821 | 124 | 1.53 | 1.23 | 1.92 | De novo MR analysis, 2022 |
| **Atrial fibrillation** | **Meta-analysis** | **67961** | **618605** |  | **1.33** | **1.18** | **1.48** |  |
| Coronary artery disease | UK Biobank | 29278 | 338365 | 126 | 1.71 | 1.49 | 1.98 | Larsson et al, 2020 |
| Coronary artery disease | CARDIoGRAMplusC4D | 60801 | 123504 | 126 | 1.55 | 1.33 | 1.81 | Larsson et al, 2020 |
| Coronary artery disease | FinnGen | 37854 | 222551 | 124 | 1.39 | 1.20 | 1.61 | De novo MR analysis, 2022 |
| **Coronary artery disease** | **Meta-analysis** | **127933** | **684420** |  | **1.55** | **1.42** | **1.69** |  |
| Deep vein thrombosis | UK Biobank | 9454 | 358189 | 126 | 1.35 | 1.1 | 1.65 | Larsson et al, 2020 |
| Deep vein thrombosis | FinnGen | 5632 | 225735 | 124 | 1.51 | 1.15 | 1.97 | De novo MR analysis, 2022 |
| **Deep vein thrombosis** | **Meta-analysis** | **15086** | **583924** |  | **1.41** | **1.20** | **1.65** |  |
| Heart failure | UK Biobank | 6712 | 360931 | 126 | 2.58 | 1.99 | 3.34 | Larsson et al, 2020 |
| Heart failure | FinnGen | 15838 | 229946 | 124 | 1.41 | 1.17 | 1.71 | De novo MR analysis, 2022 |
| **Heart failure** | **Meta-analysis** | **22550** | **590877** |  | **1.74** | **1.49** | **2.03** |  |
| Hypertension | UK Biobank | 125846 | 241797 | 126 | 1.44 | 1.28 | 1.63 | Larsson et al, 2020 |
| Hypertension | FinnGen | 53941 | 192355 | 124 | 1.48 | 1.23 | 1.76 | De novo MR analysis, 2022 |
| **Hypertension** | **Meta-analysis** | **179787** | **434152** |  | **1.45** | **1.31** | **1.61** |  |
| Intracerebral hemorrhage | UK Biobank | 1064 | 366579 | 126 | 1.32 | 0.74 | 2.33 | Larsson et al, 2020 |
| Intracerebral hemorrhage | ISGC | 1545 | 1481 | 109 | 1.28 | 0.57 | 2.88 | Larsson et al, 2019 |
| Intracerebral hemorrhage | FinnGen | 2008 | 238850 | 124 | 1.69 | 1.09 | 2.61 | De novo MR analysis, 2022 |
| **Intracerebral hemorrhage** | **Meta-analysis** | **4617** | **606910** |  | **1.50** | **1.09** | **2.06** |  |
| Ischemic stroke | UK Biobank | 4602 | 363041 | 126 | 1.72 | 1.30 | 2.26 | Larsson et al, 2020 |
| Ischemic stroke | MEGASTROKE | 34217 | 404630 | 126 | 1.21 | 1.06 | 1.39 | Larsson et al, 2019 |
| Ischemic stroke | FinnGen | 12948 | 240124 | 124 | 1.24 | 1.02 | 1.52 | De novo MR analysis, 2022 |
| **Ischemic stroke** | **Meta-analysis** | **51767** | **1007795** |  | **1.28** | **1.15** | **1.42** |  |
| Peripheral artery disease | UK Biobank | 3415 | 364228 | 126 | 4.61 | 3.30 | 6.45 | Larsson et al, 2020 |
| Peripheral artery disease | Million Veteran Program | 24009 | 150983 | 105 | 2.13 | 1.78 | 2.56 | Levin et al, 2020 |
| Peripheral artery disease | FinnGen | 9021 | 244907 | 124 | 2.52 | 1.95 | 3.26 | De novo MR analysis, 2022 |
| **Peripheral artery disease** | **Meta-analysis** | **36445** | **760118** |  | **2.53** | **2.21** | **2.90** |  |
| Pulmonary embolism | UK Biobank | 6148 | 361495 | 126 | 1.66 | 1.27 | 2.17 | Larsson et al, 2020 |
| Pulmonary embolism | FinnGen | 5130 | 254771 | 124 | 1.49 | 1.12 | 1.97 | De novo MR analysis, 2022 |
| **Pulmonary embolism** | **Meta-analysis** | **11278** | **616266** |  | **1.58** | **1.30** | **1.92** |  |
| Subarachnoid hemorrhage | UK Biobank | 1084 | 366559 | 126 | 1.77 | 1.01 | 3.12 | Larsson et al, 2020 |
| Subarachnoid hemorrhage | ISGC | 4196 | 59544 | 85 | 2.99 | 1.55 | 5.74 | Karhunen et al, 2021 |
| Subarachnoid hemorrhage | FinnGen | 1620 | 238926 | 124 | 1.29 | 0.78 | 2.14 | De novo MR analysis, 2022 |
| **Subarachnoid hemorrhage** | **Meta-analysis** | **6900** | **665029** |  | **1.77** | **1.28** | **2.45** |  |
| Thoracic aortic aneurysm | UK Biobank | 347 | 367296 | 126 | 1.74 | .64 | 4.73 | Larsson et al, 2020 |
| Transient ischemic attack | UK Biobank | 3962 | 363681 | 126 | 1.53 | 1.14 | 2.06 | Larsson et al, 2020 |
| Transient ischemic attack | FinnGen | 10830 | 240124 | 124 | 1.37 | 1.10 | 1.70 | De novo MR analysis, 2022 |
| **Transient ischemic attack** | **Meta-analysis** | **14792** | **603805** |  | **1.42** | **1.20** | **1.70** |  |
| Varicose veins | UK Biobank | 8763 | 352432 | 126 | 1.52 | 1.16 | 2.00 | Yuan et al, 2021 |
| Varicose veins | FinnGen | 13928 | 153951 | 126 | 1.16 | 0.91 | 1.48 | Yuan et al, 2021 |
| **Varicose veins** | **Meta-analysis** | **22691** | **506383** |  | **1.31** | **1.09** | **1.57** |  |
| Venous thromboembolism | UK Biobank | 14097 | 353546 | 126 | 1.46 | 1.23 | 1.75 | Larsson et al, 2020 |
| Venous thromboembolism | Million Veteran Program | 8929 | 181337 | 126 | 1.28 | 1.00 | 1.63 | Larsson et al, 2020 |
| Venous thromboembolism | FinnGen | 11288 | 249117 | 124 | 1.42 | 1.14 | 1.75 | De novo MR analysis, 2022 |
| **Venous thromboembolism** | **Meta-analysis** | **34314** | **784000** |  | **1.40** | **1.25** | **1.58** |  |
| **DIGESTIVE SYSTEM** |  |  |  |  |  |  |  |  |
| Crohn's disease | GWAS meta-analysis | 12194 | 25042 | 107 | 1.31 | 0.82 | 2.11 | Jones et al, 2020 |
| Crohn's disease | FinnGen | 810 | 249705 | 124 | 0.95 | 0.46 | 1.97 | De novo MR analysis, 2022 |
| **Crohn's disease** | **Meta-analysis** | **13004** | **274747** |  | **1.19** | **0.80** | **1.77** |  |
| GERD | FinnGen | 16043 | 225008 | 124 | 1.27 | 1.06 | 1.52 | De novo MR analysis, 2022 |
| Gallstone disease | UK Biobank | 10520 | 350674 | 126 | 1.64 | 1.33 | 2.02 | Yuan et al, 2021 |
| Gallstone disease | FinnGen | 11675 | 121348 | 126 | 1.23 | 0.97 | 1.57 | Yuan et al, 2021 |
| **Gallstone disease** | **Meta-analysis** | **22195** | **472022** |  | **1.45** | **1.24** | **1.70** |  |
| Periodontitis | GLIDE consortium | 12289 | 22326 | 109 | 1.26 | 1.04 | 1.53 | Baumeister et al, 2021 |
| Periodontitis | FinnGen | 14631 | 245774 | 124 | 1.81 | 1.51 | 2.16 | De novo MR analysis, 2022 |
| **Periodontitis** | **Meta-analysis** | **26920** | **268100** |  | **1.53** | **1.34** | **1.75** |  |
| Ulcerative colitis | GWAS meta-analysis | 12366 | 25042 | 107 | 0.78 | 0.58 | 1.05 | Jones et al, 2020 |
| Ulcerative colitis | FinnGen | 2768 | 249705 | 124 | 0.76 | 0.49 | 1.16 | De novo MR analysis, 2022 |
| **Ulcerative colitis** | **Meta-analysis** | **15134** | **274747** |  | **0.77** | **0.61** | **0.99** |  |
| **NERVOUS SYSTEM** |  |  |  |  |  |  |  |  |
| ALS | GWAS meta-analysis | 20806 | 59804 | 115 | 0.94 | 0.74 | 1.19 | Opie-Martin et al, 2020 |
| ALS | FinnGen | 262 | 131274 | 124 | 0.71 | 0.22 | 2.30 | De novo MR analysis, 2022 |
| **ALS** | **Meta-analysis** | **21068** | **191078** |  | **0.93** | **0.74** | **1.17** |  |
| Alzheimer's disease | FinnGen | 7329 | 252879 | 124 | 1.03 | 0.79 | 1.33 | De novo MR analysis, 2022 |
| Multiple sclerosis | IMSGC | 14802 | 26703 | 111 | 1.06 | 0.82 | 1.38 | Vandebergh & Goris 2020 |
| Multiple sclerosis | FinnGen | 1364 | 258340 | 124 | 1.25 | .74 | 2.11 | De novo MR analysis, 2022 |
| **Multiple sclerosis** | **Meta-analysis** | **16166** | **285043** |  | **1.10** | **0.87** | **1.38** |  |
| Parkinson's disease | FinnGen | 2496 | 257909 | 124 | 0.68 | 0.46 | 1.01 | De novo MR analysis, 2022 |
| **MUSCULOSKELETAL SYSTEM** |  |  |  |  |  |  |  |  |
| Fracture | FinnGen | 4819 | 251409 | 124 | 1.20 | 0.89 | 1.63 | De novo MR analysis, 2022 |
| Gout | GUGC | 3576 | 203546 | 91 | 1.42 | 0.86 | 2.35 | De novo MR analysis, 2022 |
| Gout | FinnGen | 4502 | 241230 | 124 | 1.04 | 0.77 | 1.41 | De novo MR analysis, 2022 |
| **Gout** | **Meta-analysis** | **8078** | **444776** |  | **1.13** | **0.87** | **1.46** |  |
| Osteoarthritis | UK Biobank & arcOGEN | 60800 | 328251 | 126 | 2.23 | 1.85 | 2.68 | Gill et al, 2021 |
| Osteoarthritis | FinnGen | 45467 | 173414 | 124 | 1.62 | 1.37 | 1.92 | De novo MR analysis, 2022 |
| **Osteoarthritis** | **Meta-analysis** | **106267** | **501665** |  | **1.87** | **1.65** | **2.12** |  |
| Rheumatoid arthritis | GWAS meta-analysis | 14361 | 43923 | 367 | 1.55 | 1.13 | 2.14 | Qian et al, 2020 |
| Rheumatoid arthritis | FinnGen | 8428 | 251977 | 124 | 1.34 | 1.02 | 1.75 | De novo MR analysis, 2022 |
| **Rheumatoid arthritis** | **Meta-analysis** | **22789** | **295900** |  | **1.42** | **1.16** | **1.75** |  |
| **ENDOCRINE & METABOLIC** |  |  |  |  |  |  |  |  |
| Polycystic ovary syndrome | FinnGen | 797 | 140558 | 124 | **0.73** | **0.35** | **1.53** | De novo MR analysis, 2022 |
| Type 2 diabetes | FinnGen | 11006 | 82655 | 124 | 1.19 | 0.93 | 1.51 | Yuan & Larsson, 2020 |
| Type 2 diabetes | DIAGRAM consortium | 74124 | 223213 | 126 | 1.18 | 0.98 | 1.41 | Yuan & Larsson, 2020 |
| **Type 2 diabetes** | **Meta-analysis** | **85130** | **305868** |  | **1.18** | **1.02** | **1.37** |  |
| **EYE DISEASES** |  |  |  |  |  |  |  |  |
| AMD | IAMDGC | 16144 | 17832 | 119 | 1.32 | 1.09 | 1.59 | Kuan et al, 2021 |
| AMD | FinnGen | 4645 | 243951 | 124 | 1.25 | 0.88 | 1.78 | De novo MR analysis, 2022 |
| **AMD** | **Meta-analysis** | **20789** | **261783** |  | **1.30** | **1.10** | **1.54** |  |
| Primary OAG | GWAS meta-analysis | 133492 | 90939 | 108 | 0.80 | 0.64 | 1.01 | De novo MR analysis, 2022 |
| Primary OAG | FinnGen | 4164 | 249920 | 124 | 1.05 | 0.74 | 1.49 | De novo MR analysis, 2022 |
| **Primary OAG** | **Meta-analysis** | **137656** | **340859** |  | **0.87** | **0.72** | **1.05** |  |
| Senile cataract | FinnGen | 26758 | 189604 | 124 | **1.09** | **0.93** | **1.29** | De novo MR analysis, 2022 |
| **NEOPLASMS** |  |  |  |  |  |  |  |  |
| Bladder cancer | UK Biobank | 2588 | 365055 | 126 | 2.00 | 1.39 | 2.90 | De novo MR analysis, 2022 |
| Bladder cancer | FinnGen | 1701 | 258704 | 124 | 1.75 | 1.07 | 2.88 | De novo MR analysis, 2022 |
| **Bladder cancer** | **Meta-analysis** | **4289** | **623759** |  | **1.91** | **1.42** | **2.56** |  |
| Brain cancer | UK Biobank | 810 | 366833 | 126 | 1.06 | 0.55 | 2.05 | De novo MR analysis, 2022 |
| Brain cancer | FinnGen | 615 | 259790 | 124 | 1.35 | 0.60 | 3.03 | De novo MR analysis, 2022 |
| **Brain cancer** | **Meta-analysis** | **1425** | **626623** |  | **1.17** | **0.70** | **1.94** |  |
| Breast cancer | UK Biobank | 13666 | 185172 | 126 | 0.91 | 0.75 | 1.09 | De novo MR analysis, 2022 |
| Breast cancer | BCAC | 122977 | 105974 | 122 | 1.12 | 1.00 | 1.25 | De novo MR analysis, 2022 |
| Breast cancer | FinnGen | 11573 | 135488 | 124 | 0.87 | 0.71 | 1.07 | De novo MR analysis, 2022 |
| **Breast cancer** | **Meta-analysis** | **148216** | **426634** |  | **1.02** | **0.94** | **1.12** |  |
| Cervical cancer | UK Biobank | 1928 | 196910 | 126 | 2.34 | 1.53 | 3.59 | De novo MR analysis, 2022 |
| Cervical cancer | FinnGen | 2229 | 144832 | 124 | 1.73 | 1.09 | 2.73 | De novo MR analysis, 2022 |
| **Cervical cancer** | **Meta-analysis** | **4157** | **341742** |  | **2.03** | **1.49** | **2.78** |  |
| Colorectal cancer | UK Biobank | 5486 | 362157 | 126 | 1.06 | 0.79 | 1.42 | De novo MR analysis, 2022 |
| Colorectal cancer | CORECT and GECCO | 52775 | 45940 | 126 | 1.21 | 1.04 | 1.40 | Dimou et al, 2021 |
| Colorectal cancer | FinnGen | 4401 | 256004 | 124 | 1.17 | 0.84 | 1.63 | De novo MR analysis, 2022 |
| **Colorectal cancer** | **Meta-analysis** | **62662** | **664101** |  | **1.18** | **1.04** | **1.33** |  |
| Esophageal cancer | UK Biobank | 843 | 366800 | 126 | 2.67 | 1.4 | 5.08 | De novo MR analysis, 2022 |
| Esophageal cancer | FinnGen | 358 | 260047 | 124 | 2.01 | 0.71 | 5.75 | De novo MR analysis, 2022 |
| **Esophageal cancer** | **Meta-analysis** | **1201** | **626847** |  | **2.47** | **1.43** | **4.27** |  |
| Head/neck cancer | UK Biobank | 1615 | 366028 | 126 | 3.66 | 2.27 | 5.9 | De novo MR analysis, 2022 |
| Head/neck cancer | GAME-ON GWAS | 6034 | 6585 | 108 | 3.47 | 2.39 | 5.03 | Gormley et al, 2020 |
| Head/neck cancer | FinnGen | 208 | 260197 | 124 | 3.59 | 0.95 | 13.66 | De novo MR analysis, 2022 |
| **Head/neck cancer** | **Meta-analysis** | **1823** | **626225** |  | **3.54** | **2.66** | **4.72** |  |
| Kidney cancer | UK Biobank | 1310 | 366333 | 126 | 1.68 | 1.00 | 2.81 | De novo MR analysis, 2022 |
| Kidney cancer | FinnGen | 1393 | 259012 | 124 | 1.32 | 0.77 | 2.26 | De novo MR analysis, 2022 |
| **Kidney cancer** | **Meta-analysis** | **2703** | **625345** |  | **1.50** | **1.03** | **2.17** |  |
| Leukemia, any type | UK Biobank | 1403 | 366240 | 126 | 0.99 | 0.60 | 1.62 | De novo MR analysis, 2022 |
| Leukemia, lymphoid | FinnGen | 808 | 259597 | 124 | 1.17 | 0.59 | 2.31 | De novo MR analysis, 2022 |
| Leukemia, myeloid | FinnGen | 340 | 260065 | 124 | 1.13 | 0.40 | 3.17 | De novo MR analysis, 2022 |
| Lung cancer | UK Biobank | 2838 | 364805 | 126 | 7.16 | 4.91 | 10.43 | De novo MR analysis, 2022 |
| Lung cancer | FinnGen | 1681 | 217111 | 124 | 4.77 | 2.61 | 8.73 | De novo MR analysis, 2022 |
| **Lung cancer** | **Meta-analysis** | **4519** | **581916** |  | **6.39** | **4.64** | **8.80** |  |
| Melanoma | UK Biobank | 4869 | 362774 | 126 | 0.74 | 0.50 | 1.10 | De novo MR analysis, 2022 |
| Melanoma | FinnGen | 143 | 260262 | 124 | 4.84 | 0.98 | 23.83 | De novo MR analysis, 2022 |
| **Melanoma** | **Meta-analysis** | **5012** | **623036** |  | **0.82** | **0.56** | **1.21** |  |
| Multiple myeloma | UK Biobank | 656 | 366987 | 126 | 0.93 | 0.44 | 1.97 | De novo MR analysis, 2022 |
| Multiple myeloma | FinnGen | 693 | 259712 | 124 | 0.60 | 0.29 | 1.24 | De novo MR analysis, 2022 |
| **Multiple myeloma** | **Meta-analysis** | **1349** | **626699** |  | **0.74** | **0.44** | **1.25** |  |
| Non-Hodgkin lymphoma | UK Biobank | 2296 | 365347 | 126 | 1.12 | 0.76 | 1.65 | De novo MR analysis, 2022 |
| Non-Hodgkin lymphoma | FinnGen | 643 | 259762 | 124 | 1.55 | 0.73 | 3.30 | De novo MR analysis, 2022 |
| **Non-Hodgkin lymphoma** | **Meta-analysis** | **2939** | **625109** |  | **1.20** | **0.85** | **1.69** |  |
| Ovarian cancer | UK Biobank | 1520 | 197318 | 126 | 1.06 | 0.65 | 1.71 | De novo MR analysis, 2022 |
| Ovarian cancer | OCAC | 25509 | 40941 | 122 | 1.23 | 1.03 | 1.47 | De novo MR analysis, 2022 |
| Ovarian cancer | FinnGen | 1041 | 146020 | 124 | 1.15 | 0.63 | 2.08 | De novo MR analysis, 2022 |
| **Ovarian cancer** | **Meta-analysis** | **28070** | **384279** |  | **1.20** | **1.03** | **1.41** |  |
| Pancreatic cancer | UK Biobank | 1264 | 366379 | 126 | 1.82 | 1.05 | 3.15 | De novo MR analysis, 2022 |
| Pancreatic cancer | FinnGen | 881 | 259524 | 124 | 1.50 | 0.76 | 2.95 | De novo MR analysis, 2022 |
| **Pancreatic cancer** | **Meta-analysis** | **2145** | **625903** |  | **1.69** | **1.10** | **2.58** |  |
| Prostate cancer | UK Biobank | 7872 | 160876 | 126 | 0.92 | 0.70 | 1.21 | De novo MR analysis, 2022 |
| Prostate cancer | PRACTICAL | 79148 | 61106 | 122 | 0.84 | 0.71 | 0.98 | De novo MR analysis, 2022 |
| Prostate cancer | FinnGen | 8709 | 104635 | 124 | 1.02 | 0.80 | 1.30 | De novo MR analysis, 2022 |
| **Prostate cancer** | **Meta-analysis** | **95729** | **326617** |  | **0.90** | **0.80** | **1.01** |  |
| Stomach cancer | UK Biobank | 736 | 366907 | 126 | 2.49 | 1.23 | 5.02 | De novo MR analysis, 2022 |
| Stomach cancer | FinnGen | 889 | 259516 | 124 | 0.81 | 0.42 | 1.55 | De novo MR analysis, 2022 |
| **Stomach cancer** | **Meta-analysis** | **1625** | **626423** |  | **1.36** | **0.84** | **2.20** |  |
| Testicular cancer | UK Biobank | 735 | 168013 | 126 | 0.92 | 0.43 | 2.00 | De novo MR analysis, 2022 |
| Testicular cancer | FinnGen | 268 | 113076 | 124 | 0.51 | 0.16 | 1.62 | De novo MR analysis, 2022 |
| **Testicular cancer** | **Meta-analysis** | **1003** | **281089** |  | **0.77** | **0.41** | **1.46** |  |
| Uterine cancer | UK Biobank | 1931 | 196907 | 126 | 0.87 | 0.57 | 1.33 | De novo MR analysis, 2022 |
| Uterine cancer | FinnGen | 1430 | 145631 | 124 | 0.83 | 0.47 | 1.45 | De novo MR analysis, 2022 |
| **Uterine cancer** | **Meta-analysis** | **3361** | **342538** |  | **0.86** | **0.61** | **1.20** |  |

ALS, amyotrophic lateral sclerosis; BCAC, Breast Cancer Association Consortium; CARDIoGRAMplusC4D, Coronary ARtery DIsease Genome-wide Replication and Meta-analysis plus The Coronary Artery Disease Genetics consortium; CORECT, Colorectal Transdisciplary Study; DIAGRAM, DIAbetes Genetics Replication And Meta-analysis Consortium; GAME-ON, Genetic Associations and Mechanisms in Oncology; GECCO, Genetics and Epidemiology of Colorectal Cancer Consortium; GEFOS, GEnetic Factors for OSteoporosis Consortium; GERD, gastroesophageal reflux disease; GLIDE, Gene-Lifestyle Interactions in Dental Endpoints; GUGC, Global Urate Genetics Consortium; GWAS, genome-wide association; study; HERMES, Heart failure molecular epidemiology for therapeutic targets; ILCCO, International Lung Cancer Consortium; IMSGC, International Multiple Sclerosis Genetics Consortium; IPDGC, International Parkinson Disease Genomics Consortium; IAMDGC, International Age-related Macular Degeneration Genomics Consortium; ISGC, International Stroke Genetics Consortium; LB, lower bound of the 95% confidence interval; OCAC, Ovarian Cancer Association Consortium; OR, odds ratio; PGC, Psychiatric Genomics Consortium; PRACTICAL, Prostate Cancer Association Group to Investigate Cancer Associated Alterations in Genome; UB, upper bound of the 95% confidence interval.

**Table S4.** Sensitivity analysis results for genetic liability to **smoking initiation** and studied outcomes based on the weighted median

and MR-Egger methods

| **Disease** | **Study** | **Cases** | **Noncases** | **SNPs** | **Weighted median** | | |  | **MR- Egger** | | | ***P-*value for MR-Egger intercept*** | **First author, year** |
| --- | --- | --- | --- | --- | --- | --- | --- | --- | --- | --- | --- | --- | --- |
| **CIRCULATORY SYSTEM** |  |  |  |  | **OR** | **LB** | **UB** |  | **OR** | **LB** | **UB** |  |  |
| Abdominal aortic aneurysm | UK Biobank | 1094 | 366549 | 361 | 1.79 | 1.21 | 2.64 |  | 0.96 | 0.32 | 2.89 | 0.28 | Larsson et al, 2020 |
| Aortic valve stenosis | UK Biobank | 2244 | 365399 | 361 | 1.40 | 1.07 | 1.43 |  | 1.59 | 0.73 | 3.45 | 0.49 | Larsson et al, 2020 |
| Atrial fibrillation | GWAS meta-analysis | 65446 | 522744 | 374 | 1.09 | 1.01 | 1.17 |  | NA | NA | NA | NA | Lu et al, 2021 |
| Atrial fibrillation | FinnGen | 28670 | 135821 | 297 | 1.28 | 1.12 | 1.47 |  | 1.74 | 1.12 | 2.71 | 0.112 | De novo MR analysis, 2022 |
| **Atrial fibrillation** | **Meta-analysis** | **94116** | **658565** |  | **1.13** | **1.06** | **1.21** |  | **1.74** | **1.12** | **2.71** |  |  |
| Coronary artery disease | UK Biobank | 29278 | 338365 | 361 | 1.33 | 1.22 | 1.45 |  | 1.30 | 0.98 | 1.73 | 0.78 | Larsson et al, 2020 |
| Coronary artery disease | CARDIoGRAMplusC4D | 60801 | 123504 | 361 | 1.24 | 1.13 | 1.36 |  | 1.34 | 0.99 | 1.80 | 0.69 | Larsson et al, 2020 |
| Coronary artery disease | FinnGen | 37854 | 222551 | 297 | 1.33 | 1.20 | 1.47 |  | 1.60 | 1.17 | 2.18 | 0.181 | De novo MR analysis, 2022 |
| **Coronary artery disease** | **Meta-analysis** | **127933** | **684420** |  | **1.30** | **1.23** | **1.37** |  | **1.40** | **1.18** | **1.66** |  |  |
| Deep vein thrombosis | UK Biobank | 9454 | 358189 | 361 | 1.33 | 1.16 | 1.52 |  | 1.69 | 1.13 | 2.53 | 0.18 | Larsson et al, 2020 |
| Deep vein thrombosis | FinnGen | 5632 | 225735 | 297 | 1.27 | 1.03 | 1.55 |  | 1.35 | 0.73 | 2.49 | 0.791 | De novo MR analysis, 2022 |
| **Deep vein thrombosis** | **Meta-analysis** | **15086** | **583924** |  | **1.31** | **1.17** | **1.47** |  | **1.58** | **1.13** | **2.21** |  |  |
| Heart failure | HERMES consortium | 47309 | 930014 | 359 | 1.51 | 1.29 | 1.76 |  | 1.20 | 0.75 | 1.92 | 0.30 | van Oort et al, 2020 |
| Heart failure | FinnGen | 15838 | 229946 | 297 | 1.20 | 1.05 | 1.37 |  | 1.72 | 1.19 | 2.49 | 0.059 | De novo MR analysis, 2022 |
| **Heart failure** | **Meta-analysis** | **63147** | **1159960** |  | **1.32** | **1.20** | **1.46** |  | **1.50** | **1.12** | **2.00** |  |  |
| Hypertension | UK Biobank | 125846 | 241797 | 361 | 1.15 | 1.10 | 1.21 |  | 1.16 | 0.92 | 1.45 | 0.70 | Larsson et al, 2020 |
| Hypertension | FinnGen | 53941 | 192355 | 297 | 1.11 | 1.01 | 1.23 |  | 1.25 | 0.88 | 1.77 | 0.827 | De novo MR analysis, 2022 |
| **Hypertension** | **Meta-analysis** | **179787** | **434152** |  | **1.14** | **1.09** | **1.19** |  | **1.19** | **0.98** | **1.44** |  |  |
| Intracerebral hemorrhage | UK Biobank | 1064 | 366579 | 361 | 1.19 | 0.80 | 1.78 |  | 0.55 | 0.18 | 1.67 | 0.16 | Larsson et al, 2020 |
| Intracerebral hemorrhage | ISGC | 1545 | 1481 | 326 | 0.83 | 0.51 | 1.34 |  | 0.49 | 0.11 | 2.01 | 0.24 | Larsson et al, 2019 |
| Intracerebral hemorrhage | FinnGen | 2008 | 238850 | 297 | 1.19 | 0.86 | 1.65 |  | 2.64 | 1.05 | 6.64 | 0.104 | De novo MR analysis, 2022 |
| **Intracerebral hemorrhage** | **Meta-analysis** | **4617** | **606910** |  | **1.10** | **0.88** | **1.38** |  | **1.14** | **0.60** | **2.16** |  |  |
| Ischemic stroke | UK Biobank | 4602 | 363041 | 361 | 1.36 | 1.13 | 1.65 |  | 1.07 | 0.63 | 1.82 | 0.45 | Larsson et al, 2020 |
| Ischemic stroke | MEGASTROKE | 34217 | 404630 | 372 | 1.22 | 1.12 | 1.34 |  | 1.06 | 0.80 | 1.40 | 0.25 | Larsson et al, 2019 |
| Ischemic stroke | FinnGen | 12948 | 240124 | 297 | 1.02 | 0.89 | 1.17 |  | 0.87 | 0.58 | 1.31 | 0.207 | De novo MR analysis, 2022 |
| **Ischemic stroke** | **Meta-analysis** | **51767** | **1007795** |  | **1.18** | **1.10** | **1.27** |  | **1.01** | **0.82** | **1.24** |  |  |
| Peripheral artery disease | UK Biobank | 3415 | 364228 | 361 | 1.81 | 1.44 | 2.29 |  | 1.80 | 0.94 | 3.45 | 0.99 | Larsson et al, 2020 |
| Peripheral artery disease | FinnGen | 9021 | 244907 | 297 | 1.53 | 1.29 | 1.80 |  | 1.59 | 0.98 | 2.56 | 0.916 | De novo MR analysis, 2022 |
| **Peripheral artery disease** | **Meta-analysis** | **12436** | **609135** |  | **1.62** | **1.42** | **1.85** |  | **1.66** | **1.13** | **2.44** |  |  |
| Pulmonary embolism | UK Biobank | 6148 | 361495 | 361 | 1.29 | 1.09 | 1.53 |  | 1.52 | 0.91 | 2.52 | 0.58 | Larsson et al, 2020 |
| Pulmonary embolism | FinnGen | 5130 | 254771 | 297 | 1.12 | 0.91 | 1.38 |  | 0.95 | 0.53 | 1.71 | 0.553 | De novo MR analysis, 2022 |
| **Pulmonary embolism** | **Meta-analysis** | **11278** | **616266** |  | **1.22** | **1.07** | **1.39** |  | **1.24** | **0.85** | **1.82** |  |  |
| Subarachnoid hemorrhage | UK Biobank | 1084 | 366559 | 361 | 1.26 | 0.85 | 1.89 |  | 0.77 | 0.25 | 2.32 | 0.26 | Larsson et al, 2020 |
| Subarachnoid hemorrhage | ISGC | 4196 | 59544 | 235 | 2.17 | 1.50 | 3.13 |  | 5.13 | 1.71 | 15.39 | 0.03 | Karhunen et al, 2021 |
| Subarachnoid hemorrhage | FinnGen | 1620 | 238926 | 297 | 1.15 | 0.80 | 1.66 |  | 1.48 | 0.50 | 4.34 | 0.770 | De novo MR analysis, 2022 |
| **Subarachnoid hemorrhage** | **Meta-analysis** | **6900** | **665029** |  | **1.47** | **1.19** | **1.83** |  | **1.81** | **0.96** | **3.41** |  |  |
| Thoracic aortic aneurysm | UK Biobank | 347 | 367296 | 361 | 1.22 | 0.63 | 2.34 |  | 1.32 | 0.19 | 8.99 | 0.82 | Larsson et al, 2020 |
| Transient ischemic attack | UK Biobank | 3962 | 363681 | 361 | 1.29 | 1.05 | 1.59 |  | 0.85 | 0.47 | 1.57 | 0.15 | Larsson et al, 2020 |
| Transient ischemic attack | FinnGen | 10830 | 240124 | 297 | 1.14 | 0.98 | 1.32 |  | 1.07 | 0.68 | 1.69 | 0.760 | De novo MR analysis, 2022 |
| **Transient ischemic attack** | **Meta-analysis** | **14792** | **603805** |  | **1.19** | **1.05** | **1.34** |  | **0.98** | **0.68** | **1.42** |  |  |
| Varicose veins | UK Biobank | 8763 | 352431 | 312 | 1.14 | 0.98 | 1.33 |  | 1.82 | 1.12 | 2.95 | 0.057 | Yuan et al, 2021 |
| Varicose veins | FinnGen | 20425 | 225735 | 297 | 1.15 | 0.99 | 1.34 |  | 1.74 | 1.10 | 2.73 | 0.046 | Yuan et al, 2021 |
| **Varicose veins** | **Meta-analysis** | **29188** | **578166** |  | **1.15** | **1.03** | **1.28** |  | **1.77** | **1.27** | **2.47** |  |  |
| Venous thromboembolism | UK Biobank | 14097 | 353546 | 361 | 1.35 | 1.20 | 1.51 |  | 1.58 | 1.10 | 2.27 | 0.01 | Larsson et al, 2020 |
| Venous thromboembolism | Million Veteran Program | 8929 | 181337 | 361 | 1.13 | 0.97 | 1.30 |  | 1.59 | 1.01 | 2.51 | 0.09 | Larsson et al, 2020 |
| Venous thromboembolism | FinnGen | 11288 | 249117 | 297 | 1.16 | 1.01 | 1.35 |  | 1.34 | 0.87 | 2.07 | 0.592 | De novo MR analysis, 2022 |
| **Venous thromboembolism** | **Meta-analysis** | **34314** | **784000** |  | **1.23** | **1.14** | **1.33** |  | **1.51** | **1.19** | **1.91** |  |  |
| **DIGESTIVE SYSTEM** |  |  |  |  |  |  |  |  |  |  |  |  |  |
| Crohn's disease | GWAS meta-analysis | 12194 | 25042 | 325 | 1.09 | 0.93 | 1.28 |  | 0.78 | 0.38 | 1.61 | 0.31 | Jones et al, 2020 |
| Crohn's disease | FinnGen | 810 | 249705 | 297 | 1.47 | 0.88 | 2.45 |  | 1.68 | 0.36 | 7.91 | 0.705 | De novo MR analysis, 2022 |
| **Crohn's disease** | **Meta-analysis** | **13004** | **274747** |  | **1.12** | **0.96** | **1.30** |  | **0.89** | **0.47** | **1.72** |  |  |
| Diverticular disease | UK Biobank | 12662 | 348532 | 312 | 1.25 | 1.09 | 1.43 |  | 1.12 | 0.74 | 1.68 | NA | Yuan & Larsson, 2021 |
| Diverticular disease | FinnGen | 10978 | 149001 | 297 | 1.11 | 0.95 | 1.29 |  | 1.09 | 0.68 | 1.75 | NA | Yuan & Larsson, 2021 |
| **Diverticular disease** | **Meta-analysis** | **23640** | **497533** |  | **1.19** | **1.07** | **1.31** |  | **1.11** | **0.81** | **1.51** |  |  |
| Gallstone disease | UK Biobank | 10520 | 350674 | 309 | 1.30 | 1.14 | 1.48 |  | 1.10 | 0.72 | 1.69 | 0.253 | Yuan et al, 2021 |
| Gallstone disease | FinnGen | 11675 | 121348 | 295 | 1.11 | 0.96 | 1.29 |  | 0.83 | 0.53 | 1.30 | 0.214 | Yuan et al, 2021 |
| **Gallstone disease** | **Meta-analysis** | **22195** | **472022** |  | **1.21** | **1.10** | **1.34** |  | **0.96** | **0.71** | **1.31** |  |  |
| GERD | UK Biobank & Qskin Sun & Health | 71522 | 261079 | 202 | 1.33 | 1.23 | 1.45 |  | 1.12 | 0.83 | 1.51 | 0.454 | Yuan & Larsson 2022 |
| GERD | FinnGen | 16043 | 225008 | 297 | 1.25 | 1.10 | 1.41 |  | 1.05 | 0.71 | 1.56 | 0.366 | De novo MR analysis, 2022 |
| **GERD** | **Meta-analysis** | **87565** | **486087** |  | **1.31** | **1.22** | **1.40** |  | **1.09** | **0.86** | **1.39** |  |  |
| Pancreatitis, acute | UK Biobank | 1292 | 359902 | 366 | 1.31 | 0.90 | 1.92 |  | 0.68 | 0.24 | 1.96 | 0.163 | Yuan et al, 2021 |
| Pancreatitis, acute | FinnGen | 1762 | 121348 | 352 | 1.95 | 1.42 | 2.67 |  | 2.79 | 1.14 | 6.87 | 0.244 | Yuan et al, 2021 |
| **Pancreatitis, acute** | **Meta-analysis** | **3054** | **481250** |  | **1.66** | **1.30** | **2.11** |  | **1.54** | **0.78** | **3.04** |  |  |
| Pancreatitis, chronic | UK Biobank | 246 | 360948 | 366 | 1.61 | 0.75 | 3.48 |  | 0.24 | 0.03 | 2.28 | 0.056 | Yuan et al, 2021 |
| Pancreatitis, chronic | FinnGen | 914 | 121348 | 352 | 1.72 | 1.10 | 2.69 |  | 2.97 | 0.83 | 10.67 | 0.435 | Yuan et al, 2021 |
| **Pancreatitis, chronic** | **Meta-analysis** | **1160** | **482296** |  | **1.69** | **1.15** | **2.49** |  | **1.55** | **0.52** | **4.66** |  |  |
| Periodontitis, chronic | FinnGen | 14631 | 245774 | 297 | 1.37 | 1.21 | 1.56 |  | 1.39 | 0.95 | 2.02 | NA | De novo MR analysis, 2022 |
| Ulcerative colitis | GWAS meta-analysis | 12366 | 25042 | 325 | 1.08 | 0.92 | 1.27 |  | 0.70 | 0.36 | 1.37 | 0.36 | Jones et al, 2020 |
| Ulcerative colitis | FinnGen | 2768 | 249705 | 297 | 1.04 | 0.79 | 1.38 |  | 0.70 | 0.30 | 1.64 | 0.258 | De novo MR analysis, 2022 |
| **Ulcerative colitis** | **Meta-analysis** | **15134** | **274747** |  | **1.07** | **0.93** | **1.23** |  | **0.70** | **0.41** | **1.18** |  |  |
| **NERVOUS SYSTEM** |  |  |  |  |  |  |  |  |  |  |  |  |  |
| Alzheimer's disease | PGC | 71880 | 383378 | 74 | NA | NA | NA |  | NA | NA | NA | NA | Yang et al, 2020 |
| Alzheimer's disease | FinnGen | 7329 | 252879 | 297 | 1.25 | 1.03 | 1.52 |  | 1.14 | 0.65 | 1.99 | 0.741 | De novo MR analysis, 2022 |
| **Alzheimer's disease** | **Meta-analysis** | **79209** | **636257** |  | **1.25** | **1.03** | **1.52** |  | **1.14** | **0.65** | **1.99** |  |  |
| ALS | GWAS meta-analysis | 20806 | 59804 | 301 | 1.06 | 0.93 | 1.21 |  | 1.47 | 1.04 | 2.09 | 0.18 | Opie-Martin et al, 2020 |
| ALS | FinnGen | 262 | 131274 | 297 | 1.46 | 0.61 | 3.47 |  | 3.07 | 0.26 | 36.14 | 0.474 | De novo MR analysis, 2022 |
| **ALS** | **Meta-analysis** | **21068** | **191078** |  | **1.07** | **0.94** | **1.22** |  | **1.49** | **1.06** | **2.11** |  |  |
| Epilepsy | UK Biobank | 901 | 395209 | 295 | 1.77 | 1.16 | 2.71 |  | 1.38 | 0.43 | 4.46 | 0.901 | Yuan et al, 2021 |
| Epilepsy | FinnGen | 4588 | 144780 | 295 | 1.24 | 1.00 | 1.55 |  | 1.39 | 0.74 | 2.62 | 0.608 | Yuan et al, 2021 |
| **Epilepsy** | **Meta-analysis** | **5489** | **539989** |  | **1.34** | **1.10** | **1.62** |  | **1.39** | **0.80** | **2.42** |  |  |
| Multiple sclerosis | IMSGC | 14802 | 26703 | 297 | 1.12 | 0.95 | 1.32 |  | 1.10 | 0.61 | 1.97 | 0.89 | Vandebergh & Goris 2020 |
| Multiple sclerosis | FinnGen | 1364 | 258340 | 297 | 0.87 | 0.59 | 1.28 |  | 0.79 | 0.23 | 2.66 | 0.752 | De novo MR analysis, 2022 |
| **Multiple sclerosis** | **Meta-analysis** | **16166** | **285043** |  | **1.08** | **0.93** | **1.25** |  | **1.03** | **0.61** | **1.75** |  |  |
| Parkinson's disease | IPDGC | 36752 | 929806 | 365 | 0.72 | 0.58 | 0.90 |  | 0.47 | 0.24 | 0.92 | 0.16 | Sieurin et al, 2021 |
| Parkinson's disease | FinnGen | 2496 | 257909 | 297 | 0.75 | 0.56 | 1.01 |  | 0.87 | 0.37 | 2.06 | 0.909 | De novo MR analysis, 2022 |
| **Parkinson's disease** | **Meta-analysis** | **39248** | **1187715** |  | **0.73** | **0.61** | **0.87** |  | **0.59** | **0.35** | **1.01** |  |  |
| **MUSCULOSKELETAL SYSTEM** |  |  |  |  |  |  |  |  |  |  |  |  |  |
| Fracture | GEFOS | 53184 | 373611 | 377 | 1.10 | 1.04 | 1.17 |  | 1.22 | 1.00 | 1.50 | 0.269 | Yuan et al, 2019 |
| Fracture | FinnGen | 4819 | 251409 | 297 | 1.07 | 0.86 | 1.33 |  | 1.82 | 0.96 | 3.47 | 0.130 | De novo MR analysis, 2022 |
| **Fracture** | **Meta-analysis** | **58003** | **625020** |  | **1.10** | **1.04** | **1.16** |  | **1.26** | **1.04** | **1.53** |  |  |
| Gout | GUGC | 2115 | 67259 | 259 | 1.01 | 0.75 | 1.38 |  | 1.14 | 0.49 | 2.62 | 0.941 | De novo MR analysis, 2022 |
| Gout | FinnGen | 4502 | 241230 | 297 | 1.12 | 0.90 | 1.40 |  | 0.95 | 0.51 | 1.78 | 0.623 | De novo MR analysis, 2022 |
| **Gout** | **Meta-analysis** | **6617** | **308489** |  | **1.08** | **0.90** | **1.29** |  | **1.01** | **0.61** | **1.67** |  |  |
| Osteoarthritis | UK Biobank | 39427 | 378169 | 314 | 1.24 | 1.14 | 1.34 |  | 1.27 | 0.95 | 1.71 | 0.898 | De novo MR analysis, 2022 |
| Osteoarthritis | FinnGen | 45467 | 173414 | 297 | 1.28 | 1.17 | 1.41 |  | 1.61 | 1.17 | 2.20 | 0.202 | De novo MR analysis, 2022 |
| **Osteoarthritis** | **Meta-analysis** | **84894** | **551583** |  | **1.26** | **1.18** | **1.34** |  | **1.42** | **1.14** | **1.76** |  |  |
| Rheumatoid arthritis | GWAS meta-analysis | 14361 | 43923 | 367 | 1.44 | 1.21 | 1.72 |  | NA | NA | NA | 0.579 | Qian et al, 2020 |
| Rheumatoid arthritis | FinnGen | 8428 | 251977 | 297 | 1.29 | 1.08 | 1.54 |  | 1.17 | 0.67 | 2.02 | 0.503 | De novo MR analysis, 2022 |
| **Rheumatoid arthritis** | **Meta-analysis** | **22789** | **295900** |  | **1.36** | **1.20** | **1.54** |  | **1.17** | **0.67** | **2.02** |  |  |
| **ENDOCRINE & METABOLIC** |  |  |  |  |  |  |  |  |  |  |  |  |  |
| Polycystic ovary syndrome | GWAS meta-analysis | 4138 | 20129 | 360 | 1.29 | 0.96 | 1.71 |  | NA | NA | NA | NA | Tao et al, 2021 |
| Polycystic ovary syndrome | FinnGen | 797 | 140558 | 297 | 1.24 | 0.74 | 2.07 |  | 7.36 | 1.73 | 31.22 | 0.015 | De novo MR analysis, 2022 |
| **Polycystic ovary syndrome** | **Meta-analysis** | **4935** | **160687** |  | **1.28** | **0.99** | **1.64** |  | **7.36** | **1.73** | **31.22** |  |  |
| Type 2 diabetes | DIAGRAM consortium | 74124 | 824006 | 377 | NA | NA | NA |  | NA | NA | NA | NA | Yuan & Larsson, 2020 |
| Type 2 diabetes | FinnGen | 37002 | 215160 | 297 | 1.20 | 1.08 | 1.34 |  | 1.17 | 0.82 | 1.68 | 0.784 | De novo MR analysis, 2022 |
| **Type 2 diabetes** | **Meta-analysis** | **111126** | **1039166** |  | **1.20** | **1.08** | **1.34** |  | **1.17** | **0.82** | **1.68** |  |  |
| **EYE DISEASES** |  |  |  |  |  |  |  |  |  |  |  |  |  |
| AMD | IAMDGC | 16144 | 17832 | 336 | 1.20 | 1.02 | 1.40 |  | 1.40 | 0.87 | 2.25 | 0.65 | Kuan et al, 2021 |
| AMD | FinnGen | 4645 | 243951 | 297 | 1.10 | 0.87 | 1.38 |  | 1.22 | 0.62 | 2.41 | 0.731 | De novo MR analysis, 2022 |
| **AMD** | **Meta-analysis** | **20789** | **261783** |  | **1.17** | **1.02** | **1.33** |  | **1.34** | **0.91** | **1.97** |  |  |
| Primary OAG | GWAS meta-analysis | 133492 | 90939 | 314 | 0.93 | 0.84 | 1.03 |  | 0.83 | 0.58 | 1.19 | 0.519 | De novo MR analysis, 2022 |
| Primary OAG | FinnGen | 4164 | 249920 | 297 | 0.92 | 0.73 | 1.17 |  | 0.87 | 0.44 | 1.72 | 0.600 | De novo MR analysis, 2022 |
| **Primary OAG** | **Meta-analysis** | **137656** | **340859** |  | **0.93** | **0.85** | **1.02** |  | **0.84** | **0.61** | **1.15** |  |  |
| Senile cataract | UK Biobank | 26489 | 509767 | 312 | 1.13 | 0.95 | 1.35 |  | 1.42 | 0.82 | 2.46 | 0.544 | Yuan et al, 2022 |
| Senile cataract | FinnGen | 32692 | 224812 | 297 | 1.18 | 1.03 | 1.36 |  | 1.00 | 0.65 | 1.55 | 0.460 | Yuan et al, 2022 |
| **Senile cataract** | **Meta-analysis** | **59181** | **734579** |  | **1.16** | **1.04** | **1.29** |  | **1.14** | **0.81** | **1.61** |  |  |
| **NEOPLASMS** |  |  |  |  |  |  |  |  |  |  |  |  |  |
| Bladder cancer | UK Biobank | 2588 | 365055 | 361 | 1.31 | 1.03 | 1.68 |  | 1.32 | 0.65 | 2.68 | 0.86 | Larsson et al, 2020 |
| Bladder cancer | FinnGen | 1701 | 258704 | 297 | 1.66 | 1.17 | 2.35 |  | 1.25 | 0.46 | 3.39 | 0.870 | De novo MR analysis, 2022 |
| **Bladder cancer** | **Meta-analysis** | **4289** | **623759** |  | **1.42** | **1.16** | **1.73** |  | **1.30** | **0.73** | **2.31** |  |  |
| Brain cancer | UK Biobank | 810 | 366833 | 361 | 1.21 | 0.78 | 1.89 |  | 0.48 | 0.13 | 1.77 | 0.26 | Larsson et al, 2020 |
| Brain cancer | FinnGen | 615 | 259790 | 297 | 0.81 | 0.46 | 1.45 |  | 0.80 | 0.16 | 4.08 | 0.998 | De novo MR analysis, 2022 |
| **Brain cancer** | **Meta-analysis** | **1425** | **626623** |  | **1.04** | **0.73** | **1.48** |  | **0.59** | **0.21** | **1.62** |  |  |
| Breast cancer | UK Biobank | 13666 | 185172 | 361 | 0.98 | 0.87 | 1.09 |  | 0.97 | 0.69 | 1.38 | 0.99 | Larsson et al, 2020 |
| Breast cancer | BCAC | 122977 | 105974 | 351 | 1.08 | 1.01 | 1.15 |  | 1.01 | 0.77 | 1.32 | 0.76 | Larsson et al, 2020 |
| Breast cancer | FinnGen | 11573 | 135488 | 297 | 1.06 | 0.91 | 1.24 |  | 1.09 | 0.69 | 1.71 | 0.881 | De novo MR analysis, 2022 |
| **Breast cancer** | **Meta-analysis** | **148216** | **426634** |  | **1.06** | **1.00** | **1.11** |  | **1.01** | **0.83** | **1.23** |  |  |
| Cervical cancer | UK Biobank | 1928 | 196910 | 361 | 1.67 | 1.25 | 2.23 |  | 2.02 | 0.89 | 4.58 | 0.51 | Larsson et al, 2020 |
| Cervical cancer | FinnGen | 2229 | 144832 | 297 | 1.17 | 0.86 | 1.60 |  | 1.41 | 0.57 | 3.52 | 0.638 | De novo MR analysis, 2022 |
| **Cervical cancer** | **Meta-analysis** | **4157** | **341742** |  | **1.42** | **1.15** | **1.75** |  | **1.72** | **0.94** | **3.16** |  |  |
| Colorectal cancer | UK Biobank | 5486 | 362157 | 361 | 1.20 | 1.00 | 1.43 |  | 0.82 | 0.48 | 1.41 | 0.27 | Larsson et al, 2020 |
| Colorectal cancer | CORECT and GECCO | 52775 | 45940 | 112 | 0.97 | 0.83 | 1.13 |  | 1.10 | 0.67 | 1.79 | 0.63 | Dimou et al, 2021 |
| Colorectal cancer | FinnGen | 4401 | 256004 | 297 | 0.92 | 0.73 | 1.15 |  | 0.57 | 0.29 | 1.09 | 0.119 | De novo MR analysis, 2022 |
| **Colorectal cancer** | **Meta-analysis** | **62662** | **664101** |  | **1.03** | **0.93** | **1.14** |  | **0.85** | **0.62** | **1.17** |  |  |
| Esophageal cancer | UK Biobank | 843 | 366800 | 361 | 2.00 | 1.28 | 3.12 |  | 1.59 | 0.44 | 5.82 | 0.83 | Larsson et al, 2020 |
| Esophageal cancer | FinnGen | 358 | 260047 | 190 | 1.85 | 0.88 | 3.89 |  | 8.91 | 1.09 | 72.48 | 0.097 | De novo MR analysis, 2022 |
| **Esophageal cancer** | **Meta-analysis** | **1201** | **626847** |  | **1.96** | **1.34** | **2.87** |  | **2.55** | **0.85** | **7.67** |  |  |
| Head/neck cancer | UK Biobank | 1615 | 366028 | 361 | 1.35 | 0.99 | 1.85 |  | 1.02 | 0.42 | 2.50 | 0.47 | Larsson et al, 2020 |
| Head/neck cancer | GAME-ON GWAS | 6034 | 6585 | 176 | 2.72 | 1.46 | 5.05 |  | 5.34 | 0.47 | 58.7 | NA | Gormley et al, 2020 |
| Head/neck cancer | FinnGen | 208 | 260197 | 297 | 1.78 | 0.66 | 4.77 |  | 1.88 | 0.12 | 29.83 | 0.816 | De novo MR analysis, 2022 |
| **Head/neck cancer** | **Meta-analysis** | **7857** | **632810** |  | **1.57** | **1.20** | **2.06** |  | **1.29** | **0.58** | **2.87** |  |  |
| Kidney cancer | UK Biobank | 1310 | 366333 | 361 | 1.20 | 0.84 | 1.70 |  | 1.00 | 0.34 | 2.94 | 0.64 | Larsson et al, 2020 |
| Kidney cancer | FinnGen | 1393 | 259012 | 297 | 1.40 | 0.95 | 2.06 |  | 0.88 | 0.30 | 2.60 | 0.363 | De novo MR analysis, 2022 |
| **Kidney cancer** | **Meta-analysis** | **2703** | **625345** |  | **1.29** | **0.99** | **1.67** |  | **0.94** | **0.39** | **2.77** |  |  |
| Leukemia | UK Biobank | 1403 | 366240 | 361 | 1.11 | 0.79 | 1.56 |  | 1.04 | 0.39 | 2.76 | 0.97 | Larsson et al, 2020 |
| Leukemia, lymphoid | FinnGen | 808 | 259597 | 297 | 0.77 | 0.47 | 1.27 |  | 1.92 | 0.47 | 7.92 | 0.218 | De novo MR analysis, 2022 |
| Leukemia, myeloid | FinnGen | 340 | 260065 | 297 | 1.95 | 0.90 | 4.23 |  | 1.78 | 0.20 | 15.55 | 0.958 | De novo MR analysis, 2022 |
| Lung cancer | UK Biobank | 2838 | 364805 | 361 | 2.23 | 1.75 | 2.83 |  | 2.22 | 1.12 | 4.38 | 0.96 | Larsson et al, 2020 |
| Lung cancer | ILCCO | 11348 | 15861 | 346 | 1.82 | 1.53 | 2.16 |  | 2.90 | 1.76 | 4.79 | 0.05 | Larsson et al, 2020 |
| Lung cancer | FinnGen | 3061 | 257344 | 297 | 1.55 | 1.18 | 2.03 |  | 2.96 | 1.27 | 6.90 | 0.138 | De novo MR analysis, 2022 |
| **Lung cancer** | **Meta-analysis** | **17247** | **638010** |  | **1.86** | **1.64** | **2.10** |  | **2.70** | **1.87** | **3.88** |  |  |
| Melanoma | UK Biobank | 4869 | 362774 | 361 | 0.99 | 0.82 | 1.20 |  | 1.07 | 0.59 | 1.93 | 0.63 | Larsson et al, 2020 |
| Melanoma | FinnGen | 143 | 260262 | 297 | 2.13 | .65 | 6.92 |  | 3.66 | 0.12 | 110.62 | 0.634 | De novo MR analysis, 2022 |
| **Melanoma** | **Meta-analysis** | **5012** | **623036** |  | **1.01** | **0.84** | **1.22** |  | **1.10** | **0.62** | **1.99** |  |  |
| Multiple myeloma | UK Biobank | 656 | 366987 | 361 | 0.93 | 0.56 | 1.54 |  | 1.80 | 0.43 | 7.61 | 0.40 | Larsson et al, 2020 |
| Multiple myeloma | FinnGen | 693 | 259712 | 297 | 0.77 | 0.45 | 1.31 |  | 1.59 | 0.35 | 7.29 | 0.340 | De novo MR analysis, 2022 |
| **Multiple myeloma** | **Meta-analysis** | **1349** | **626699** |  | **0.85** | **0.59** | **1.23** |  | **1.69** | **0.60** | **4.82** |  |  |
| Non-Hodgkin lymphoma | UK Biobank | 2296 | 365347 | 361 | 0.79 | 0.60 | 1.03 |  | 1.28 | 0.59 | 2.80 | 0.32 | Larsson et al, 2020 |
| Non-Hodgkin lymphoma | FinnGen | 643 | 259762 | 297 | 0.73 | 0.42 | 1.27 |  | 0.26 | 0.05 | 1.28 | 0.089 | De novo MR analysis, 2022 |
| **Non-Hodgkin lymphoma** | **Meta-analysis** | **2939** | **625109** |  | **0.78** | **0.61** | **0.99** |  | **0.95** | **0.47** | **1.92** |  |  |
| Ovarian cancer | UK Biobank | 1520 | 197318 | 361 | 1.05 | 0.76 | 1.45 |  | 0.40 | 0.16 | 1.01 | 0.03 | Larsson et al, 2020 |
| Ovarian cancer | OCAC | 25509 | 40941 | 351 | 1.07 | 0.94 | 1.21 |  | 1.03 | 0.70 | 1.50 | 0.95 | Larsson et al, 2020 |
| Ovarian cancer | FinnGen | 1041 | 146020 | 297 | 0.75 | 0.48 | 1.17 |  | 1.08 | 0.31 | 3.74 | 0.711 | De novo MR analysis, 2022 |
| **Ovarian cancer** | **Meta-analysis** | **28070** | **384279** |  | **1.04** | **0.93** | **1.17** |  | **0.91** | **0.65** | **1.28** |  |  |
| Pancreatic cancer | UK Biobank | 1264 | 366379 | 361 | 1.24 | 0.87 | 1.77 |  | 1.42 | 0.51 | 4.01 | 0.67 | Larsson et al, 2020 |
| Pancreatic cancer | PanScan & PanC4 | 8769 | 7055 | 188 | 0.92 | 0.71 | 1.19 |  | 0.83 | 0.40 | 1.74 | 0.85 | Lu et al, 2020 |
| Pancreatic cancer | FinnGen | 881 | 259524 | 297 | 1.04 | 0.65 | 1.67 |  | 1.03 | 0.26 | 3.99 | 0.869 | De novo MR analysis, 2022 |
| **Pancreatic cancer** | **Meta-analysis** | **10914** | **632958** |  | **1.02** | **0.85** | **1.24** |  | **1.00** | **0.58** | **1.73** |  |  |
| Prostate cancer | UK Biobank | 7872 | 160876 | 361 | 0.95 | 0.81 | 1.11 |  | 0.66 | 0.40 | 1.10 | 0.21 | Larsson et al, 2020 |
| Prostate cancer | PRACTICAL | 79148 | 61106 | 350 | 0.90 | 0.83 | 0.97 |  | 0.64 | 0.46 | 0.88 | 0.03 | Larsson et al, 2020 |
| Prostate cancer | FinnGen | 8709 | 104635 | 297 | 0.88 | 0.73 | 1.06 |  | 0.60 | 0.34 | 1.06 | 0.106 | De novo MR analysis, 2022 |
| **Prostate cancer** | **Meta-analysis** | **95729** | **326617** |  | **0.91** | **0.85** | **0.97** |  | **0.64** | **0.50** | **0.82** |  |  |
| Stomach cancer | UK Biobank | 736 | 366907 | 361 | 1.35 | 0.85 | 2.16 |  | 4.29 | 1.09 | 16.95 | 0.11 | Larsson et al, 2020 |
| Stomach cancer | FinnGen | 889 | 259516 | 297 | 1.00 | 0.62 | 1.61 |  | 4.63 | 1.20 | 17.86 | 0.029 | De novo MR analysis, 2022 |
| **Stomach cancer** | **Meta-analysis** | **1625** | **626423** |  | **1.17** | **0.84** | **1.63** |  | **4.46** | **1.70** | **11.67** |  |  |
| Testicular cancer | UK Biobank | 735 | 168013 | 361 | 1.19 | 0.74 | 1.90 |  | 1.63 | 0.38 | 6.93 | 0.58 | Larsson et al, 2020 |
| Testicular cancer | FinnGen | 268 | 113076 | 297 | 0.60 | 0.25 | 1.43 |  | 0.09 | 0.01 | 1.02 | 0.076 | De novo MR analysis, 2022 |
| **Testicular cancer** | **Meta-analysis** | **1003** | **281089** |  | **1.02** | **0.67** | **1.54** |  | **0.72** | **0.21** | **2.46** |  |  |
| Uterine cancer | UK Biobank | 1931 | 196907 | 361 | 0.98 | 0.72 | 1.32 |  | 0.60 | 0.25 | 1.43 | 0.15 | Larsson et al, 2020 |
| Uterine cancer | FinnGen | 1430 | 145631 | 297 | 0.95 | 0.65 | 1.40 |  | 0.48 | 0.16 | 1.45 | 0.237 | De novo MR analysis, 2022 |
| **Uterine cancer** | **Meta-analysis** | **3361** | **342538** |  | **0.97** | **0.76** | **1.23** |  | **0.55** | **0.28** | **1.09** |  |  |

ALS, amyotrophic lateral sclerosis; BCAC, Breast Cancer Association Consortium; CARDIoGRAMplusC4D, Coronary ARtery DIsease Genome-wide Replication and Meta-analysis plus The Coronary Artery Disease Genetics consortium; CORECT, Colorectal Transdisciplary Study; DIAGRAM, DIAbetes Genetics Replication And Meta-analysis Consortium; GAME-ON, Genetic Associations and Mechanisms in Oncology; GECCO, Genetics and Epidemiology of Colorectal Cancer Consortium; GEFOS, GEnetic Factors for OSteoporosis Consortium; GERD, gastroesophageal reflux disease; GLIDE, Gene-Lifestyle Interactions in Dental Endpoints; GUGC, Global Urate Genetics Consortium; GWAS, genome-wide association; study; HERMES, Heart failure molecular epidemiology for therapeutic targets; ILCCO, International Lung Cancer Consortium; IMSGC, International Multiple Sclerosis Genetics Consortium; IPDGC, International Parkinson Disease Genomics Consortium; IAMDGC, International Age-related Macular Degeneration Genomics Consortium; ISGC, International Stroke Genetics Consortium; LB, lower bound of the 95% confidence interval; OCAC, Ovarian Cancer Association Consortium; NA, not available; OR, odds ratio; PGC, Psychiatric Genomics Consortium; PRACTICAL, Prostate Cancer Association Group to Investigate Cancer Associated Alterations in Genome; UB, upper bound of the 95% confidence interval.

*A *P* value <0.05 provides evidence of possible pleiotropy.

**Table S5.** Sensitivity analysis results for genetic liability to **lifetime smoking** and the studied outcomes based on the weighted median and MR-Egger methods

| **Disease** | | **Study** | **Cases** | **Noncases** | **SNPs** | **Weighted median** | | |  | **MR-Egger** | | | ***P-*value for MR-Egger intercept*** | **Reference** |
| --- | --- | --- | --- | --- | --- | --- | --- | --- | --- | --- | --- | --- | --- | --- |
| **CIRCULATORY SYSTEM** | |  |  |  |  | OR | LB | UB |  | OR | LB | UB |  |  |
| Abdominal aortic aneurysm | | UK Biobank | 1094 | 366549 | 126 | 7.75 | 3.30 | 18.2 |  | 20.6 | 2.22 | 190.8 | 0.23 | Larsson et al, 2020 |
| Aortic valve stenosis | | UK Biobank | 2244 | 365399 | 126 | 2.25 | 1.24 | 4.08 |  | 7.04 | 1.26 | 39.4 | 0.09 | Larsson et al, 2020 |
| Atrial fibrillation | | UK Biobank | 16945 | 350698 | 126 | 1.37 | 1.10 | 1.70 |  | 1.49 | 0.75 | 2.95 | 0.96 | Larsson et al, 2020 |
| Atrial fibrillation | | AF Consortium | 22346 | 132086 | 126 | 1.02 | 0.79 | 1.33 |  | 0.84 | 0.37 | 1.91 | 0.60 | Larsson et al, 2020 |
| Atrial fibrillation | | FinnGen | 28670 | 135821 | 124 | 1.64 | 1.25 | 2.17 |  | 2.03 | 0.79 | 5.23 | 0.551 | De novo MR analysis, 2022 |
| **Atrial fibrillation** | | **Meta-analysis** | **67961** | **618605** |  | **1.32** | **1.14** | **1.52** |  | **1.34** | **0.85** | **2.12** |  |  |
| Coronary artery disease | | UK Biobank | 29278 | 338365 | 126 | 1.62 | 1.37 | 1.92 |  | 1.52 | 0.87 | 2.67 | 0.66 | Larsson et al, 2020 |
| Coronary artery disease | | CARDIoGRAMplusC4D | 60801 | 123504 | 126 | 1.66 | 1.37 | 2.01 |  | 0.66 | 0.35 | 1.24 | 0.006 | Larsson et al, 2020 |
| Coronary artery disease | | FinnGen | 37854 | 222551 | 124 | 1.40 | 1.14 | 1.72 |  | 1.60 | 0.86 | 2.97 | 0.649 | De novo MR analysis, 2022 |
| **Coronary artery disease** | | **Meta-analysis** | **127933** | **684420** |  | **1.57** | **1.41** | **1.75** |  | **1.20** | **0.85** | **1.70** |  |  |
| Deep vein thrombosis | | UK Biobank | 9454 | 358189 | 126 | 1.33 | 0.98 | 1.81 |  | 1.30 | 0.58 | 2.88 | 0.92 | Larsson et al, 2020 |
| Deep vein thrombosis | | FinnGen | 5632 | 225735 | 124 | 1.64 | 1.10 | 2.45 |  | 1.28 | 0.41 | 3.97 | 0.772 | De novo MR analysis, 2022 |
| **Deep vein thrombosis** | | **Meta-analysis** | **15086** | **583924** |  | **1.44** | **1.13** | **1.83** |  | **1.29** | **0.67** | **2.49** |  |  |
| Heart failure | | UK Biobank | 6712 | 360931 | 126 | 2.99 | 2.09 | 4.28 |  | 2.87 | 1.03 | 8.04 | 0.83 | Larsson et al, 2020 |
| Heart failure | | FinnGen | 15838 | 229946 | 124 | 1.40 | 1.07 | 1.82 |  | 3.78 | 1.72 | 8.28 | 0.012 | De novo MR analysis, 2022 |
| **Heart failure** | | **Meta-analysis** | **22550** | **590877** |  | **1.83** | **1.48** | **2.27** |  | **3.42** | **1.83** | **6.37** |  |  |
| Hypertension | | UK Biobank | 125846 | 241797 | 126 | 1.40 | 1.24 | 1.58 |  | 1.11 | 0.69 | 1.77 | 0.25 | Larsson et al, 2020 |
| Hypertension | | FinnGen | 53941 | 192355 | 124 | 1.43 | 1.18 | 1.74 |  | 1.03 | 0.52 | 2.04 | 0.272 | De novo MR analysis, 2022 |
| **Hypertension** | | **Meta-analysis** | **179787** | **434152** |  | **1.41** | **1.27** | **1.56** |  | **1.08** | **0.74** | **1.60** | **0.351** |  |
| Intracerebral hemorrhage | | UK Biobank | 1064 | 366579 | 126 | 0.97 | 0.41 | 2.28 |  | 0.90 | 0.09 | 8.61 | 0.73 | Larsson et al, 2020 |
| Intracerebral hemorrhage | | ISGC | 1545 | 1481 | 109 | 0.99 | 0.32 | 3.06 |  | 0.11 | 0.004 | 2.81 | 0.13 | Larsson et al, 2019 |
| Intracerebral hemorrhage | | FinnGen | 2008 | 238850 | 124 | 1.68 | 0.89 | 3.18 |  | 1.97 | 0.32 | 12.20 | 0.866 | De novo MR analysis, 2022 |
| **Intracerebral hemorrhage** | | **Meta-analysis** | **4617** | **606910** |  | **1.31** | **0.82** | **2.08** |  | **0.97** | **0.26** | **3.57** |  |  |
| Ischemic stroke | | UK Biobank | 4602 | 363041 | 126 | 1.75 | 1.15 | 2.67 |  | 3.66 | 1.22 | 10.90 | 0.16 | Larsson et al, 2020 |
| Ischemic stroke | | MEGASTROKE | 34217 | 404630 | 126 | 1.18 | 0.97 | 1.44 |  | 0.93 | 0.54 | 1.60 | 0.32 | Larsson et al, 2019 |
| Ischemic stroke | | FinnGen | 2948 | 240124 | 124 | 1.11 | 0.84 | 1.46 |  | 0.85 | 0.37 | 1.95 | 0.351 | De novo MR analysis, 2022 |
| **Ischemic stroke** | | **Meta-analysis** | **51767** | **1007795** |  | **1.22** | **1.05** | **1.42** |  | **1.11** | **0.73** | **1.68** |  |  |
| Peripheral artery disease | | UK Biobank | 3415 | 364228 | 126 | 5.12 | 3.10 | 8.48 |  | 11.03 | 2.94 | 41.30 | 0.18 | Larsson et al, 2020 |
| Peripheral artery disease | | Million Veteran Program | 24009 | 150983 | 105 | 1.94 | 1.53 | 2.44 |  | NA | NA | NA | NA | Levin et al, 2020 |
| Peripheral artery disease | | FinnGen | 9021 | 244907 | 124 | 2.45 | 1.73 | 3.45 |  | 7.80 | 2.72 | 22.4 | 0.030 | De novo MR analysis, 2022 |
| **Peripheral artery disease** | | **Meta-analysis** | **36445** | **760118** |  | **2.34** | **1.96** | **2.81** |  | **8.93** | **3.92** | **20.35** |  |  |
| Pulmonary embolism | | UK Biobank | 6148 | 361495 | 126 | 1.68 | 1.17 | 2.40 |  | 1.14 | 0.39 | 3.33 | 0.48 | Larsson et al, 2020 |
| Pulmonary embolism | | FinnGen | 5130 | 254771 | 124 | 1.13 | 0.75 | 1.72 |  | 0.98 | 0.30 | 3.19 | 0.478 | De novo MR analysis, 2022 |
| **Pulmonary embolism** | | **Meta-analysis** | **11278** | **616266** |  | **1.42** | **1.08** | **1.86** |  | **1.06** | **0.48** | **2.36** |  |  |
| Subarachnoid hemorrhage | | UK Biobank | 1084 | 366559 | 126 | 2.09 | 0.91 | 4.82 |  | 6.59 | 0.70 | 61.79 | 0.23 | Larsson et al, 2020 |
| Subarachnoid hemorrhage | | ISGC | 4196 | 59544 | 85 | 3.33 | 1.79 | 6.22 |  | 8.05 | 0.92 | 70.43 | 0.392 | Karhunen et al, 2021 |
| Subarachnoid hemorrhage | | FinnGen | 1620 | 238926 | 124 | 0.86 | 0.42 | 1.79 |  | 1.18 | 0.14 | 9.85 | 0.932 | De novo MR analysis, 2022 |
| **Subarachnoid hemorrhage** | | **Meta-analysis** | **6900** | **665029** |  | **1.92** | **1.28** | **2.90** |  | **3.87** | **1.10** | **13.58** |  |  |
| Thoracic aortic aneurysm | | UK Biobank | 347 | 367296 | 126 | 2.62 | 0.60 | 11.40 |  | 30.89 | 0.59 | 1617.0 | 0.14 | Larsson et al, 2020 |
| Transient ischemic attack | | UK Biobank | 3962 | 363681 | 126 | 1.68 | 1.10 | 2.56 |  | 2.01 | 0.62 | 6.53 | 0.64 | Larsson et al, 2020 |
| Transient ischemic attack | | FinnGen | 10830 | 240124 | 124 | 1.40 | 1.04 | 1.89 |  | 1.49 | 0.60 | 3.72 | 0.850 | De novo MR analysis, 2022 |
| **Transient ischemic attack** | | **Meta-analysis** | **14792** | **603805** |  | **1.49** | **1.17** | **1.90** |  | **1.67** | **0.81** | **3.43** |  |  |
| Varicose veins | | UK Biobank | 8763 | 352432 | 126 | 1.26 | 0.91 | 1.75 |  | 2.27 | 0.77 | 6.69 | 0.456 | Yuan et al, 2021 |
| Varicose veins | | FinnGen | 13928 | 153951 | 126 | 1.17 | 0.86 | 1.59 |  | 0.90 | 0.33 | 2.48 | 0.610 | Yuan et al, 2021 |
| **Varicose veins** | | **Meta-analysis** | **22691** | **506383** |  | **1.21** | **0.97** | **1.52** |  | **1.38** | **0.66** | **2.89** |  |  |
| Venous thromboembolism | | UK Biobank | 14097 | 353546 | 126 | 1.49 | 1.17 | 1.91 |  | 1.09 | 0.54 | 2.18 | 0.39 | Larsson et al, 2020 |
| Venous thromboembolism | | Million Veteran Program | 8929 | 181337 | 126 | 1.33 | 0.96 | 1.84 |  | 0.81 | 0.31 | 2.14 | 0.35 | Larsson et al, 2020 |
| Venous thromboembolism | | FinnGen | 11288 | 249117 | 124 | 1.41 | 1.05 | 1.91 |  | 1.16 | 0.47 | 2.85 | 0.655 | De novo MR analysis, 2022 |
| **Venous thromboembolism** | | **Meta-analysis** | **34314** | **784000** |  | **1.42** | **1.21** | **1.68** |  | **1.03** | **0.64** | **1.67** |  |  |
| **DIGESTIVE SYSTEM** | |  |  |  |  |  |  |  |  |  |  |  |  |  |
| Crohn's disease | | GWAS meta-analysis | 12194 | 25042 | 107 | 1.63 | 0.95 | 2.78 |  | NA | NA | NA | NA | Jones et al, 2020 |
| Crohn's disease | | FinnGen | 810 | 249705 | 124 | 1.11 | 0.40 | 3.06 |  | 4.86 | 0.23 | 102.71 | 0.280 | De novo MR analysis, 2022 |
| **Crohn's disease** | | **Meta-analysis** | **13004** | **274747** |  | **1.50** | **0.93** | **2.41** |  | **4.86** | **0.23** | **102.70** |  |  |
| GERD | | FinnGen | 16043 | 225008 | 124 | 1.35 | 1.05 | 1.74 |  | 0.69 | 0.33 | 1.44 | 0.094 | De novo MR analysis, 2022 |
| Gallstone disease | | UK Biobank | 10520 | 350674 | 126 | 1.58 | 1.20 | 2.07 |  | 0.62 | 0.28 | 1.39 | 0.016 | Yuan et al, 2021 |
| Gallstone disease | | FinnGen | 11675 | 121348 | 126 | 1.14 | 0.84 | 1.55 |  | 0.29 | 0.11 | 0.77 | 0.004 | Yuan et al, 2021 |
| **Gallstone disease** | | **Meta-analysis** | **22195** | **472022** |  | **1.37** | **1.12** | **1.68** |  | **0.46** | **0.25** | **0.85** |  |  |
| Periodontitis | | GLIDE consortium | 12289 | 22326 | 109 | 1.24 | 0.91 | 1.70 |  | NA | NA | NA | NA | Baumeister et al, 2021 |
| Periodontitis | | FinnGen | 14631 | 245774 | 124 | 1.98 | 1.53 | 2.56 |  | 1.54 | 0.72 | 3.28 | 0.667 | De novo MR analysis, 2022 |
| **Periodontitis** | | **Meta-analysis** | **26920** | **268100** |  | **1.64** | **1.34** | **2.00** |  | **NA** | **NA** | **NA** |  |  |
| Ulcerative colitis | | GWAS meta-analysis | 12366 | 25042 | 107 | 0.89 | 0.62 | 1.28 |  | NA | NA | NA | NA | Jones et al, 2020 |
| Ulcerative colitis | | FinnGen | 2768 | 249705 | 124 | 0.64 | 0.36 | 1.14 |  | 0.18 | 0.03 | 1.07 | 0.104 | De novo MR analysis, 2022 |
| **Ulcerative colitis** | | **Meta-analysis** | **15134** | **274747** |  | **0.81** | **0.60** | **1.10** |  | **NA** | **NA** | **NA** |  |  |
| **NERVOUS SYSTEM** | |  |  |  |  |  |  |  |  |  |  |  |  |  |
| ALS | | GWAS meta-analysis | 20806 | 59804 | 115 | 0.81 | 0.60 | 1.08 |  | 0.47 | 0.19 | 1.12 | NA | Opie-Martin et al, 2020 |
| ALS | | FinnGen | 262 | 131274 | 124 | 0.94 | 0.16 | 5.34 |  | 0.51 | 0.001 | 71.69 | 0.893 | De novo MR analysis, 2022 |
| **ALS** | | **Meta-analysis** | **21068** | **191078** |  | **0.81** | **0.61** | **1.09** |  | **0.47** | **0.20** | **1.13** |  |  |
| Alzheimer's disease | | FinnGen | 7329 | 252879 | 124 | 0.93 | 0.63 | 1.36 |  | 0.55 | 0.19 | 1.61 | 0.241 | De novo MR analysis, 2022 |
| Multiple sclerosis | | IMSGC | 14802 | 26703 | 111 | 1.02 | 0.71 | 1.47 |  | 1.40 | 0.48 | 4.11 | NA | Vandebergh & Goris 2020 |
| Multiple sclerosis | | FinnGen | 1364 | 258340 | 124 | 1.23 | 0.57 | 2.68 |  | 1.02 | 0.11 | 9.22 | 0.854 | De novo MR analysis, 2022 |
| **Multiple sclerosis** | | **Meta-analysis** | **16166** | **285043** |  | **1.06** | **0.76** | **1.47** |  | **1.32** | **0.50** | **3.46** |  |  |
| Parkinson's disease | | FinnGen | 2496 | 257909 | 124 | 0.54 | 0.31 | 0.97 |  | 0.45 | 0.09 | 2.34 | 0.608 | De novo MR analysis, 2022 |
| **MUSCULOSKELETAL SYSTEM** | | |  |  |  |  |  |  |  |  |  |  |  |  |
| Fracture | FinnGen | | 4819 | 251409 | 124 | 1.32 | 0.86 | 2.04 |  | 1.34 | 0.37 | 4.78 | 0.867 | De novo MR analysis, 2022 |
| Gout | GUGC | | 2115 | 67259 | 91 | 1.09 | 0.51 | 2.31 |  | 1.78 | 0.22 | 14.08 | 0.783 | De novo MR analysis, 2022 |
| Gout | FinnGen | | 4502 | 241230 | 124 | 0.94 | 0.61 | 1.47 |  | 1.73 | 0.48 | 6.18 | 0.418 | De novo MR analysis, 2022 |
| **Gout** | **Meta-analysis** | | **6617** | **308489** |  | **0.98** | **0.67** | **1.43** |  | **1.74** | **0.59** | **5.18** |  |  |
| Osteoarthritis | UK Biobank & arcOGEN | | 60800 | 328251 | 126 |  |  |  |  | NA | NA | NA | NA | Gill et al, 2021 |
| Osteoarthritis | FinnGen | | 45467 | 173414 | 124 | 1.56 | 1.27 | 1.91 |  | 1.13 | 0.56 | 2.29 | 0.303 | De novo MR analysis, 2022 |
| **Osteoarthritis** | **Meta-analysis** | | **106267** | **501665** |  |  |  |  |  | **NA** | **NA** | **NA** |  |  |
| Rheumatoid arthritis | GWAS meta-analysis | | 14361 | 43923 | 367 | 1.52 | 1.02 | 2.26 |  | NA | NA | NA | 0.645 | Qian et al, 2020 |
| Rheumatoid arthritis | FinnGen | | 8428 | 251977 | 124 | 1.48 | 1.05 | 2.08 |  | 2.38 | 0.78 | 7.30 | 0.300 | De novo MR analysis, 2022 |
| **Rheumatoid arthritis** | **Meta-analysis** | | **22789** | **295900** |  | **1.50** | **1.16** | **1.94** |  | **NA** | **NA** | **NA** |  |  |
| **ENDOCRINE & METABOLIC** |  | |  |  |  |  |  |  |  |  |  |  |  |  |
| Polycystic ovary syndrome | FinnGen | | 797 | 140558 | 124 | 0.50 | 0.18 | 1.42 |  | 0.23 | 0.01 | 5.27 | 0.461 | De novo MR analysis, 2022 |
| Type 2 diabetes | FinnGen | | 11006 | 82655 | 124 | NA | NA | NA |  | NA | NA | NA | NA | Yuan & Larsson, 2020 |
| Type 2 diabetes | DIAGRAM consortium | | 74124 | 223213 | 126 | 1.52 | 1.30 | 1.78 |  | 0.62 | 0.32 | 1.20 | 0.004 | Yuan & Larsson, 2020 |
| **Type 2 diabetes** | **Meta-analysis** | | **85130** | **305868** |  | NA | NA | NA |  | NA | NA | NA |  |  |
| **EYE DISEASES** |  | |  |  |  |  |  |  |  |  |  |  |  |  |
| AMD | IAMDGC | | 16144 | 17832 | 119 | 1.42 | 1.11 | 1.81 |  | 1.87 | 0.88 | 3.99 | 0.35 | Kuan et al, 2021 |
| AMD | FinnGen | | 4645 | 243951 | 124 | 1.19 | 0.75 | 1.89 |  | 1.44 | 0.33 | 6.32 | 0.844 | De novo MR analysis, 2022 |
| **AMD** | **Meta-analysis** | | **20789** | **261783** |  | **1.36** | **1.10** | **1.70** |  | **1.77** | **0.90** | **3.47** |  |  |
| Primary OAG | GWAS meta-analysis | | 133492 | 90939 | 108 | 0.91 | 0.71 | 1.16 |  | 1.17 | 0.49 | 2.79 | 0.394 | De novo MR analysis, 2022 |
| Primary OAG | FinnGen | | 4164 | 249920 | 124 | 1.02 | 0.63 | 1.65 |  | 0.88 | 0.20 | 3.88 | 0.812 | De novo MR analysis, 2022 |
| **Primary OAG** | **Meta-analysis** | | **137656** | **340859** |  | **0.93** | **0.75** | **1.16** |  | **1.09** | **0.51** | **2.30** |  |  |
| Senile cataract | FinnGen | | 26758 | 189604 | 124 | 1.10 | 0.88 | 1.37 |  | 0.83 | 0.41 | 1.65 | 0.414 | De novo MR analysis, 2022 |
| **NEOPLASMS** |  | |  |  |  |  |  |  |  |  |  |  |  |  |
| Bladder cancer | UK Biobank | | 2588 | 365055 | 126 | 1.81 | 1.05 | 3.12 |  | 2.40 | 0.56 | 10.35 | 0.803 | De novo MR analysis, 2022 |
| Bladder cancer | FinnGen | | 1701 | 258704 | 124 | 1.98 | 0.99 | 3.96 |  | 1.07 | 0.14 | 8.54 | 0.633 | De novo MR analysis, 2022 |
| **Bladder cancer** | **Meta-analysis** | | **4289** | **623759** |  | **1.87** | **1.22** | **2.87** |  | **1.83** | **0.57** | **6.02** |  |  |
| Brain cancer | UK Biobank | | 810 | 366833 | 126 | 1.27 | 0.48 | 3.37 |  | 0.74 | 0.06 | 9.88 | 0.777 | De novo MR analysis, 2022 |
| Brain cancer | FinnGen | | 615 | 259790 | 124 | 1.31 | 0.41 | 4.17 |  | 18.53 | 0.65 | 530.08 | 0.115 | De novo MR analysis, 2022 |
| **Brain cancer** | **Meta-analysis** | | **1425** | **626623** |  | **1.29** | **0.61** | **2.71** |  | **2.41** | **0.32** | **18.38** |  |  |
| Breast cancer | UK Biobank | | 13666 | 185172 | 126 | 0.87 | 0.67 | 1.12 |  | 0.72 | 0.35 | 1.52 | 0.545 | De novo MR analysis, 2022 |
| Breast cancer | BCAC | | 122977 | 105974 | 122 | 1.16 | 1.02 | 1.33 |  | 1.40 | 0.90 | 2.19 | 0.306 | De novo MR analysis, 2022 |
| Breast cancer | FinnGen | | 11573 | 135488 | 124 | 0.98 | 0.73 | 1.34 |  | 0.96 | 0.41 | 2.24 | 0.832 | De novo MR analysis, 2022 |
| **Breast cancer** | **Meta-analysis** | | **148216** | **426634** |  | **1.08** | **0.96** | **1.20** |  | **1.13** | **0.80** | **1.60** |  |  |
| Cervical cancer | UK Biobank | | 1928 | 196910 | 126 | 2.48 | 1.33 | 4.62 |  | 1.45 | 0.27 | 7.84 | 0.566 | De novo MR analysis, 2022 |
| Cervical cancer | FinnGen | | 2229 | 144832 | 124 | 1.71 | .93 | 3.16 |  | 6.64 | 0.98 | 45.19 | 0.156 | De novo MR analysis, 2022 |
| **Cervical cancer** | **Meta-analysis** | | **4157** | **341742** |  | **2.05** | **1.33** | **3.18** |  | **2.82** | **0.80** | **9.97** |  |  |
| Colorectal cancer | UK Biobank | | 5486 | 362157 | 126 | 1.28 | 0.86 | 1.89 |  | 0.93 | 0.29 | 3.00 | 0.817 | De novo MR analysis, 2022 |
| Colorectal cancer | CORECT and GECCO | | 52775 | 45940 | 126 | 1.23 | 1.01 | 1.50 |  | 1.58 | 0.87 | 2.89 | NA | Dimou et al, 2021 |
| Colorectal cancer | FinnGen | | 4401 | 256004 | 124 | 1.11 | 0.70 | 1.75 |  | 1.28 | 0.32 | 5.12 | 0.895 | De novo MR analysis, 2022 |
| **Colorectal cancer** | **Meta-analysis** | | **62662** | **664101** |  | **1.22** | **1.04** | **1.44** |  | **1.40** | **0.85** | **2.30** |  |  |
| Esophageal cancer | UK Biobank | | 843 | 366800 | 126 | 4.11 | 1.61 | 10.55 |  | 17.50 | 1.38 | 222.30 | 0.134 | De novo MR analysis, 2022 |
| Esophageal cancer | FinnGen | | 358 | 260047 | 124 | 3.67 | 0.81 | 16.6 |  | 2.41 | 0.03 | 197.36 | 0.934 | De novo MR analysis, 2022 |
| **Esophageal cancer** | **Meta-analysis** | | **1201** | **626847** |  | **3.98** | **1.79** | **8.84** |  | **10.65** | **1.18** | **96.12** |  |  |
| Head/neck cancer | UK Biobank | | 1615 | 366028 | 126 | 3.63 | 1.80 | 7.33 |  | 2.20 | 0.33 | 14.65 | 0.588 | De novo MR analysis, 2022 |
| Head/neck cancer | FinnGen | | 208 | 260197 | 124 | 2.95 | 0.42 | 20.67 |  | 103.88 | 0.40 | 27215.06 | 0.223 | De novo MR analysis, 2022 |
| **Head/neck cancer** | **Meta-analysis** | | **1823** | **626225** |  | **3.54** | **1.83** | **6.86** |  | **3.29** | **0.55** | **19.78** |  |  |
| Kidney cancer | UK Biobank | | 1310 | 366333 | 126 | 1.42 | 0.66 | 3.04 |  | 0.57 | 0.07 | 4.34 | 0.279 | De novo MR analysis, 2022 |
| Kidney cancer | FinnGen | | 1393 | 259012 | 124 | 1.38 | 0.64 | 2.98 |  | 2.08 | 0.22 | 20.01 | 0.683 | De novo MR analysis, 2022 |
| **Kidney cancer** | **Meta-analysis** | | **2703** | **625345** |  | **1.40** | **0.81** | **2.41** |  | **1.03** | **0.22** | **4.71** |  |  |
| Leukemia, any type | UK Biobank | | 1403 | 366240 | 126 | 0.94 | 0.46 | 1.95 |  | 1.62 | 0.23 | 11.63 | 0.610 | De novo MR analysis, 2022 |
| Leukemia, lymphoid | FinnGen | | 808 | 259597 | 124 | 1.33 | 0.49 | 3.61 |  | 2.09 | 0.12 | 36.25 | 0.683 | De novo MR analysis, 2022 |
| Leukemia, myeloid | FinnGen | | 340 | 260065 | 124 | 0.84 | 0.18 | 3.84 |  | 4.86 | 0.06 | 369.13 | 0.495 | De novo MR analysis, 2022 |
| Lung cancer | UK Biobank | | 2838 | 364805 | 126 | 6.40 | 3.77 | 10.85 |  | 50.01 | 11.71 | 213.59 | 0.007 | De novo MR analysis, 2022 |
| Lung cancer | FinnGen | | 1681 | 217111 | 124 | 2.74 | 1.59 | 4.73 |  | 319.84 | 54.19 | 1887.85 | <0.001 | De novo MR analysis, 2022 |
| **Lung cancer** | **Meta-analysis** | | **4519** | **581916** |  | **4.24** | **2.90** | **6.20** |  | **105.20** | **34.19** | **323.67** |  |  |
| Melanoma | UK Biobank | | 4869 | 362774 | 126 | 0.63 | 0.42 | 0.96 |  | 0.99 | 0.21 | 4.64 | 0.712 | De novo MR analysis, 2022 |
| Melanoma | FinnGen | | 143 | 260262 | 124 | 3.51 | 0.33 | 37.1 |  | 1.66 | 0.001 | 1296.78 | 0.745 | De novo MR analysis, 2022 |
| **Melanoma** | **Meta-analysis** | | **5012** | **623036** |  | **0.66** | **0.44** | **1.00** |  | **1.01** | **0.22** | **4.60** |  |  |
| Multiple myeloma | UK Biobank | | 656 | 366987 | 126 | 0.69 | 0.23 | 2.03 |  | 0.34 | 0.02 | 6.65 | 0.491 | De novo MR analysis, 2022 |
| Multiple myeloma | FinnGen | | 693 | 259712 | 124 | 0.53 | 0.18 | 1.56 |  | 0.20 | 0.01 | 4.24 | 0.470 | De novo MR analysis, 2022 |
| **Multiple myeloma** | **Meta-analysis** | | **1349** | **626699** |  | **0.60** | **0.28** | **1.30** |  | **0.26** | **0.03** | **2.14** |  |  |
| Non-Hodgkin lymphoma | UK Biobank | | 2296 | 365347 | 126 | 0.81 | 0.45 | 1.44 |  | 2.14 | 0.46 | 10.03 | 0.391 | De novo MR analysis, 2022 |
| Non-Hodgkin lymphoma | FinnGen | | 643 | 259762 | 124 | 2.77 | 0.91 | 8.44 |  | 0.30 | 0.01 | 6.98 | 0.291 | De novo MR analysis, 2022 |
| **Non-Hodgkin lymphoma** | **Meta-analysis** | | **2939** | **625109** |  | 1.05 | **0.63** | **1.77** |  | **1.50** | **0.37** | **6.04** |  |  |
| Ovarian cancer | UK Biobank | | 1520 | 197318 | 126 | 1.03 | 0.51 | 2.10 |  | 3.80 | 0.57 | 25.32 | 0.172 | De novo MR analysis, 2022 |
| Ovarian cancer | OCAC | | 25509 | 40941 | 122 | 1.24 | 0.94 | 1.63 |  | 1.39 | 1.69 | 2.83 | 0.718 | De novo MR analysis, 2022 |
| Ovarian cancer | FinnGen | | 1041 | 146020 | 124 | 0.77 | 0.32 | 1.82 |  | 1.29 | 0.11 | 15.8 | 0.922 | De novo MR analysis, 2022 |
| **Ovarian cancer** | **Meta-analysis** | | **28070** | **384279** |  | **1.17** | **0.91** | **1.49** |  | **1.41** | **1.10** | **1.82** |  |  |
| Pancreatic cancer | UK Biobank | | 1264 | 366379 | 126 | 1.99 | 0.90 | 4.39 |  | 0.18 | 0.02 | 1.51 | 0.028 | De novo MR analysis, 2022 |
| Pancreatic cancer | FinnGen | | 881 | 259524 | 124 | 0.82 | 0.31 | 2.15 |  | 0.97 | 0.06 | 16.46 | 0.753 | De novo MR analysis, 2022 |
| **Pancreatic cancer** | **Meta-analysis** | | **2145** | **625903** |  | **1.40** | **0.76** | **2.58** |  | **0.34** | **0.061** | **1.87** |  |  |
| Prostate cancer | UK Biobank | | 7872 | 160876 | 126 | 0.98 | 0.69 | 1.39 |  | 0.40 | 0.13 | 1.18 | 0.117 | De novo MR analysis, 2022 |
| Prostate cancer | PRACTICAL | | 79148 | 61106 | 122 | 0.75 | 0.62 | 0.89 |  | 0.56 | 0.29 | 1.05 | 0.195 | De novo MR analysis, 2022 |
| Prostate cancer | FinnGen | | 8709 | 104635 | 124 | 1.01 | 0.71 | 1.45 |  | 1.35 | 0.49 | 3.74 | 0.578 | De novo MR analysis, 2022 |
| **Prostate cancer** | **Meta-analysis** | | **95729** | **326617** |  | **0.83** | **0.71** | **0.96** |  | **0.64** | **0.39** | **1.05** |  |  |
| Stomach cancer | UK Biobank | | 736 | 366907 | 126 | 2.54 | 0.91 | 7.11 |  | 35.78 | 2.30 | 557.3 | 0.049 | De novo MR analysis, 2022 |
| Stomach cancer | FinnGen | | 889 | 259516 | 124 | 0.96 | 0.37 | 2.51 |  | 9.05 | 0.60 | 135.5 | 0.071 | De novo MR analysis, 2022 |
| **Stomach cancer** | **Meta-analysis** | | **1625** | **626423** |  | **1.51** | **0.75** | **3.04** |  | **17.84** | **2.59** | **122.70** |  |  |
| Testicular cancer | UK Biobank | | 735 | 168013 | 126 | 0.64 | 0.22 | 1.81 |  | 8.36 | 0.40 | 175.33 | 0.142 | De novo MR analysis, 2022 |
| Testicular cancer | FinnGen | | 268 | 113076 | 124 | 0.41 | 0.08 | 2.21 |  | 0.02 | 0.001 | 2.61 | 0.181 | De novo MR analysis, 2022 |
| **Testicular cancer** | **Meta-analysis** | | **1003** | **281089** |  | **0.56** | **0.23** | **1.37** |  | **0.87** | **0.08** | **9.69** |  |  |
| Uterine cancer | UK Biobank | | 1931 | 196907 | 126 | 0.60 | 0.32 | 1.15 |  | 0.70 | 0.13 | 3.80 | 0.803 | De novo MR analysis, 2022 |
| Uterine cancer | FinnGen | | 1430 | 145631 | 124 | 0.69 | 0.32 | 1.52 |  | 0.15 | 0.01 | 1.52 | 0.135 | De novo MR analysis, 2022 |
| **Uterine cancer** | **Meta-analysis** | | **3361** | **342538** |  | **0.64** | **0.39** | **1.04** |  | **0.43** | **0.11** | **1.76** |  |  |

ALS, amyotrophic lateral sclerosis; BCAC, Breast Cancer Association Consortium; CARDIoGRAMplusC4D, Coronary ARtery DIsease Genome-wide Replication and Meta-analysis plus The Coronary Artery Disease Genetics consortium; CORECT, Colorectal Transdisciplary Study; DIAGRAM, DIAbetes Genetics Replication And Meta-analysis Consortium; GAME-ON, Genetic Associations and Mechanisms in Oncology; GECCO, Genetics and Epidemiology of Colorectal Cancer Consortium; GEFOS, GEnetic Factors for OSteoporosis Consortium; GERD, gastroesophageal reflux disease; GLIDE, Gene-Lifestyle Interactions in Dental Endpoints; GUGC, Global Urate Genetics Consortium; GWAS, genome-wide association; study; HERMES, Heart failure molecular epidemiology for therapeutic targets; ILCCO, International Lung Cancer Consortium; IMSGC, International Multiple Sclerosis Genetics Consortium; IPDGC, International Parkinson Disease Genomics Consortium; IAMDGC, International Age-related Macular Degeneration Genomics Consortium; ISGC, International Stroke Genetics Consortium; LB, lower bound of the 95% confidence interval; OCAC, Ovarian Cancer Association Consortium; NA, not available; OR, odds ratio; PGC, Psychiatric Genomics Consortium; PRACTICAL, Prostate Cancer Association Group to Investigate Cancer Associated Alterations in Genome; UB, upper bound of the 95% confidence interval.

*A *P* value <0.05 provides evidence of possible pleiotropy.

**Table S6.** Sensitivity analysis results for genetic liability to smoking initiation and disease risk adjusted for genetically predicted

alcohol consumption*

| **Disease** | **Study** | **Cases** | **Noncases** | **OR** | **LB** | **UB** | **Reference** |
| --- | --- | --- | --- | --- | --- | --- | --- |
| Abdominal aortic aneurysm | UK Biobank | 1094 | 366549 | 1.80 | 1.28 | 2.53 | Larsson et al, 2020 |
| Aortic valve stenosis | UK Biobank | 2244 | 365399 | 1.25 | 0.98 | 1.59 | Larsson et al, 2020 |
| Atrial fibrillation | GWAS meta-analysis | 65446 | 522744 | 1.10 | 1.02 | 1.18 | Lu et al, 2021 |
| Atrial fibrillation | FinnGen | 28670 | 135821 | 1.20 | 1.05 | 1.39 | De novo MR analysis, 2022 |
| **Atrial fibrillation** | **Meta-analysis** | **94116** | **658565** | **1.12** | **1.05** | **1.20** |  |
| Coronary artery disease | UK Biobank | 29 278 | 338365 | 1.39 | 1.27 | 1.52 | Larsson et al, 2020 |
| Coronary artery disease | CARDIoGRAMplusC4D | 60 801 | 123504 | 1.36 | 1.24 | 1.48 | Larsson et al, 2020 |
| Coronary artery disease | FinnGen | 37854 | 222551 | 1.44 | 1.31 | 1.59 | De novo MR analysis, 2022 |
| **Coronary artery disease** | **Meta-analysis** | **127 933** | **684420** | **1.39** | **1.32** | **1.47** |  |
| Deep vein thrombosis | UK Biobank | 9454 | 358189 | 1.34 | 1.18 | 1.51 | Larsson et al, 2020 |
| Deep vein thrombosis | FinnGen | 5632 | 225735 | 1.19 | 0.98 | 1.45 | De novo MR analysis, 2022 |
| **Deep vein thrombosis** | **Meta-analysis** | **15086** | **583924** | **1.30** | **1.17** | **1.44** |  |
| Heart failure | HERMES consortium | 47309 | 930014 | 1.30 | 1.18 | 1.44 | van Oort et al, 2020 |
| Heart failure | FinnGen | 15838 | 229946 | 1.22 | 1.08 | 1.37 | De novo MR analysis, 2022 |
| **Heart failure** | **Meta-analysis** | **63147** | **1159960** | **1.27** | **1.17** | **1.37** |  |
| Hypertension | UK Biobank | 125 846 | 241797 | 1.25 | 1.17 | 1.34 | Larsson et al, 2020 |
| Hypertension | FinnGen | 53941 | 192355 | 1.24 | 1.11 | 1.39 | De novo MR analysis, 2022 |
| **Hypertension** | **Meta-analysis** | **179 787** | **434152** | **1.25** | **1.18** | **1.32** |  |
| Intracerebral hemorrhage | UK Biobank | 1064 | 366579 | 1.14 | 0.81 | 1.61 | Larsson et al, 2020 |
| Intracerebral hemorrhage | FinnGen | 2008 | 238850 | 1.44 | 1.07 | 1.93 | De novo MR analysis, 2022 |
| **Intracerebral hemorrhage** | **Meta-analysis** | **3072** | **605429** | **1.30** | **1.04** | **1.63** |  |
| Ischemic stroke | UK Biobank | 4602 | 363041 | 1.30 | 1.10 | 1.53 | Larsson et al, 2020 |
| Ischemic stroke | MEGASTROKE | 34 217 | 404630 | 1.22 | 1.12 | 1.32 | Larsson et al, 2019 |
| Ischemic stroke | FinnGen | 12948 | 240124 | 1.24 | 1.09 | 1.41 | De novo MR analysis, 2022 |
| **Ischemic stroke** | **Meta-analysis** | **51767** | **1007795** | **1.24** | **1.16** | **1.32** |  |
| Peripheral artery disease | UK Biobank | 3415 | 364228 | 1.85 | 1.52 | 2.25 | Larsson et al, 2020 |
| Peripheral artery disease | FinnGen | 9021 | 244907 | 1.55 | 1.33 | 1.81 | De novo MR analysis, 2022 |
| **Peripheral artery disease** | **Meta-analysis** | **12436** | **609135** | **1.66** | **1.47** | **1.87** |  |
| Pulmonary embolism | UK Biobank | 6148 | 361495 | 1.39 | 1.19 | 1.62 | Larsson et al, 2020 |
| Pulmonary embolism | FinnGen | 5130 | 254771 | 1.20 | 1.00 | 1.45 | De novo MR analysis, 2022 |
| **Pulmonary embolism** | **Meta-analysis** | **11278** | **616266** | **1.31** | **1.16** | **1.47** |  |
| Subarachnoid hemorrhage | UK Biobank | 1084 | 366559 | 1.45 | 1.03 | 2.04 | Larsson et al, 2020 |
| Subarachnoid hemorrhage | FinnGen | 1620 | 238926 | 1.28 | 0.91 | 1.80 | De novo MR analysis, 2022 |
| **Subarachnoid hemorrhage** | **Meta-analysis** | **2704** | **605485** | **1.36** | **1.07** | **1.73** |  |
| Transient ischemic attack | UK Biobank | 3962 | 363681 | 1.36 | 1.13 | 1.65 | Larsson et al, 2020 |
| Transient ischemic attack | FinnGen | 10830 | 240124 | 1.25 | 1.08 | 1.49 | De novo MR analysis, 2022 |
| **Transient ischemic attack** | **Meta-analysis** | **14792** | **603805** | **1.30** | **1.15** | **1.46** |  |
| Venous thromboembolism | MVP | 8929 | 353546 | 1.16 | 1.01 | 1.33 | Larsson et al, 2020 |
| Venous thromboembolism | FinnGen | 11288 | 249117 | 1.55 | 1.33 | 1.81 | De novo MR analysis, 2022 |
| **Venous thromboembolism** | **Meta-analysis** | **20217** | **602663** | **1.32** | **1.19** | **1.46** |  |
| Diverticular disease | FinnGen | 17851 | 215956 | 1.19 | 1.05 | 1.35 | De novo MR analysis, 2022 |
| Gallstone disease | FinnGen | 24106 | 231644 | 1.22 | 1.09 | 1.36 | De novo MR analysis, 2022 |
| Gastroesophageal reflux disease | FinnGen | 16043 | 225008 | 1.36 | 1.20 | 1.54 | De novo MR analysis, 2022 |
| Pancreatitis, acute | FinnGen | 3716 | 231644 | 1.34 | 1.15 | 1.56 | De novo MR analysis, 2022 |
| Pancreatitis, chronic | FinnGen | 2250 | 231644 | **1.04** | **0.79** | **1.37** | De novo MR analysis, 2022 |
| Crohn's disease | FinnGen | 810 | 249705 | 1.40 | 0.86 | 2.29 | De novo MR analysis, 2022 |
| Periodontitis | FinnGen | 14631 | 245774 | 1.42 | 1.26 | 1.60 | De novo MR analysis, 2022 |
| Alzheimer's disease | FinnGen | 7329 | 252879 | 1.27 | 1.06 | 1.51 | De novo MR analysis, 2022 |
| Parkinson's disease | FinnGen | 2496 | 257909 | 0.97 | 0.74 | 1.28 | De novo MR analysis, 2022 |
| Epilepsy | FinnGen | 7224 | 208845 | 1.29 | 1.09 | 1.52 | De novo MR analysis, 2022 |
| Fracture | FinnGen | 4819 | 251409 | 1.14 | 0.93 | 1.40 | De novo MR analysis, 2022 |
| Osteoarthritis | FinnGen | 45467 | 173414 | 1.31 | 1.18 | 1.44 | De novo MR analysis, 2022 |
| Rheumatoid arthritis | GWAS meta-analysis | 14361 | 43923 | 1.37† | 1.19† | 1.58† | Qian et al, 2020 |
| Rheumatoid arthritis | FinnGen | 8428 | 251977 | 1.40 | 1.17 | 1.66 | De novo MR analysis, 2022 |
| **Rheumatoid arthritis** | **Meta-analysis** | **22789** | **295900** | **1.38** | **1.24** | **1.54** |  |
| Polycystic ovary syndrome | FinnGen | 797 | 140558 | 1.14 | 0.71 | 1.81 | De novo MR analysis, 2022 |
| Type 2 diabetes | FinnGen | 37002 | 215160 | 1.34 | 1.20 | 1.50 | De novo MR analysis, 2022 |
| Age-related macular degeneration | IAMDGC | 16144 | 17832 | 1.19 | 1.05 | 1.36 | Kuan et al, 2021 |
| Age-related macular degeneration | FinnGen | 4645 | 243951 | 1.18 | 0.95 | 1.46 | De novo MR analysis, 2022 |
| **Age-related macular degeneration** | **Meta-analysis** | **20789** | **261783** | **1.19** | **1.06** | **1.33** |  |
| Senile cataract | FinnGen | 32692 | 224812 | 1.20 | 1.07 | 1.34 | De novo MR analysis, 2022 |
| Lung cancer | UK Biobank | 2838 | 364805 | 2.37 | 1.92 | 2.92 | Larsson et al, 2020 |
| Lung cancer | ILCCO | 11 348 | 15861 | 1.77 | 1.51 | 2.06 | Larsson et al, 2020 |
| Lung cancer | FinnGen | 3061 | 257344 | 1.75 | 1.34 | 2.30 | De novo MR analysis, 2022 |
| **Lung cancer** | **Meta-analysis** | **17247** | **638010** | **1.92** | **1.72** | **2.15** |  |
| Cervical cancer | UK Biobank | 1928 | 144832 | 1.56 | 1.21 | 2.01 | Larsson et al, 2020 |
| Cervical cancer | FinnGen | 2229 | 144832 | 1.13 | 0.85 | 1.52 | De novo MR analysis, 2022 |
| **Cervical cancer** | **Meta-analysis** | **4157** | **289664** | **1.36** | **1.12** | **1.64** |  |
| Ovarian cancer | UK Biobank | 1520 | 197318 | 1.38 | 0.85 | 1.52 | Larsson et al, 2020 |
| Ovarian cancer | OCAC | 25 509 | 40941 | 1.14 | 1.01 | 1.28 | Larsson et al, 2020 |
| Ovarian cancer | FinnGen | 1041 | 146020 | 0.86 | 0.58 | 1.28 | De novo MR analysis, 2022 |
| **Ovarian cancer** | **Meta-analysis** | **28070** | **384279** | **1.15** | **1.03** | **1.27** |  |
| Prostate cancer | UK Biobank | 7872 | 160876 | 0.86 | 0.74 | 1.01 | Larsson et al, 2020 |
| Prostate cancer | PRACTICAL | 79 148 | 61106 | 0.92 | 0.83 | 1.02 | Larsson et al, 2020 |
| Prostate cancer | FinnGen | 8709 | 104635 | 0.93 | 0.77 | 1.11 | De novo MR analysis, 2022 |
| **Prostate cancer** | **Meta-analysis** | **95 729** | **326 617** | **0.91** | **0.84** | **0.98** |  |
| Bladder cancer | UK Biobank | 2588 | 365055 | 1.35 | 1.09 | 1.69 | Larsson et al, 2020 |
| Bladder cancer | FinnGen | 1701 | 258704 | 1.39 | 1.01 | 1.91 | De novo MR analysis, 2022 |
| **Bladder cancer** | **Meta-analysis** | **4289** | **623759** | **1.36** | **1.14** | **1.63** |  |
| Kidney cancer | UK Biobank | 1310 | 366333 | 1.25 | 0.69 | 1.74 | Larsson et al, 2020 |
| Kidney cancer | FinnGen | 1393 | 259012 | 1.67 | 1.18 | 2.35 | De novo MR analysis, 2022 |
| **Kidney cancer** | **Meta-analysis** | **2703** | **625345** | **1.51** | **1.14** | **1.99** |  |
| Head and neck cancer | UK Biobank | 1615 | 366028 | 1.41 | 1.07 | 1.87 | Larsson et al, 2020 |
| Head and neck cancer | FinnGen | 208 | 260197 | 1.19 | 0.49 | 2.87 | De novo MR analysis, 2022 |
| **Head and neck cancer** | **Meta-analysis** | **1823** | **626225** | **1.39** | **1.06** | **1.81** |  |
| Esophageal cancer | UK Biobank | 843 | 366800 | 1.76 | 1.18 | 2.63 | Larsson et al, 2020 |
| Esophageal cancer | FinnGen | 358 | 260047 | 1.23 | 0.63 | 2.41 | De novo MR analysis, 2022 |
| **Esophageal cancer** | **Meta-analysis** | **1201** | **626847** | **1.60** | **1.14** | **2.26** |  |
| Pancreatic cancer | UK Biobank | 1264 | 366379 | 1.10 | 0.80 | 1.51 | Larsson et al, 2020 |
| Pancreatic cancer | FinnGen | 881 | 259524 | 1.49 | 0.97 | 2.30 | De novo MR analysis, 2022 |
| **Pancreatic cancer** | **Meta-analysis** | **2145** | **625903** | **1.22** | **0.95** | **1.58** |  |
| Leukaemia | UK Biobank | 1403 | 366240 | 1.18 | 0.87 | 1.59 | Larsson et al, 2020 |
| Leukemia, myeloid | FinnGen | 340 | 260065 | 1.49 | 0.89 | 2.48 | De novo MR analysis, 2022 |

LB, lower bound of the 95% confidence interval; OR, odds ratio; UB, upper bound of the 95% confidence interval. *Results are shown for the observed smoking-disease associations (*P*<0.05) in the main (univariable inverse-variance weighted) analysis. †Adjusted for genetically predicted alcohol, education, and body mass index.
